# Genome Wide Analysis of Flowering Time Trait in Multiple Environments via High-Throughput Genotyping Technique in *Brassica napus* L.

**DOI:** 10.1371/journal.pone.0119425

**Published:** 2015-03-19

**Authors:** Lun Li, Yan Long, Libin Zhang, Jessica Dalton-Morgan, Jacqueline Batley, Longjiang Yu, Jinling Meng, Maoteng Li

**Affiliations:** 1 College of Life Science and Technology, Huazhong University of Science and Technology, Wuhan, China; 2 Hubei Bioinformatics and Molecular Imaging Key Laboratory, Huazhong University of Science and Technology, Wuhan, China; 3 National Key Lab of Crop Genetic Improvement, Huazhong Agricultural University, Wuhan, China; 4 Biotechnology Research Institute, Chinese Academy of Agricultural Sciences, Beijing, China; 5 School of Agriculture & Food Sciences, The University of Queensland, Brisbane, Australia; University of Idaho, UNITED STATES

## Abstract

The prediction of the flowering time (FT) trait in *Brassica napus* based on genome-wide markers and the detection of underlying genetic factors is important not only for oilseed producers around the world but also for the other crop industry in the rotation system in China. In previous studies the low density and mixture of biomarkers used obstructed genomic selection in *B*. *napus* and comprehensive mapping of FT related loci. In this study, a high-density genome-wide SNP set was genotyped from a double-haploid population of *B*. *napus*. We first performed genomic prediction of FT traits in *B*. *napus* using SNPs across the genome under ten environments of three geographic regions via eight existing genomic predictive models. The results showed that all the models achieved comparably high accuracies, verifying the feasibility of genomic prediction in *B*. *napus*. Next, we performed a large-scale mapping of FT related loci among three regions, and found 437 associated SNPs, some of which represented known FT genes, such as AP1 and PHYE. The genes tagged by the associated SNPs were enriched in biological processes involved in the formation of flowers. Epistasis analysis showed that significant interactions were found between detected loci, even among some known FT related genes. All the results showed that our large scale and high-density genotype data are of great practical and scientific values for *B*. *napus*. To our best knowledge, this is the first evaluation of genomic selection models in *B*. *napus* based on a high-density SNP dataset and large-scale mapping of FT loci.

## Introduction

Rapeseed (*Brassica napus*), as one of the leading sources of livestock feed, vegetable oil and biofuel, is the second most prominent oil seed crop in the world, supplying approximately 62.4 million tonnes of oilseed production per year. China is the top rapeseed oil producer in the world, yielding about 4.8 milion tonnes of oil each year (2009–2011, http://faostat.fao.org/). Rapeseed was planted mainly as a rotational crop with rice, maize, cotton or some vegetables in China [1]. The characteristic of flowering time (FT) of rapeseed is not only crucial for its own reproduction and crop yields, but also sequentially influencing the sowing time of the other crops in the crop rotation system. Therefore it’s necessary to predict the phenotypic traits for untested samples and to deploy the breeding lines with maximal benefit under given geographic conditions in China.

Recent efforts have been made in mapping genomic locations related with agronomic traits (including FT) in *B*. *napus* [2–8], which allows the breeders for the potential of marker-assisted selection (MAS) in crop breeding. Various marker systems (such as STS, SSR, etc.) were employed in most of these studies, which hindered the comparison of the marker locations that were detected in different studies [4]. Furthermore, in some work, only the markers within candidate genes were analyzed [2, 5], which led to the neglect of novel functional variants. Therefore, a comprehensive and unbiased scan of the genome is imperative.

Single nucleotide polymorphisms (SNPs) are the simplest and most prevalent type of markers across the genome. To date, with the availability of the abundance of SNPs, high-throughput technologies of simultaneously genotyping high-density have been applied to plants to unravel the genetic effects of agronomical traits and significant findings were observed, such as in *Arabidopsis thaliana* [9, 10], rice [11], maize [12] and barley [13]. However, most of these QTL mapping studies (including genome-wide association studies) used univariate approaches, which test the association of each single genotyped marker and phenotypes and selection of the markers exceeding significance levels. These univariate approaches tend to detect the common variants with large effects, filtering out the small effects due to the multiple test corrections. However, most agronomic traits are affected by a large number of variants with modest effects [14]. Moreover, the identified QTLs are reported to have low reproducibility across environments. Besides, most of the models are contingent on the additive effects, omitting epistasis effects. Therefore, the pre-identified set of identified markers has limited capacity in predicting phenotypic traits. Hence, MAS without mapping QTLs in advance is of great necessity.

To remedy the drawbacks of the conventional MAS, genomic selection (GS) was proposed by Meuwissen et al. to predict phenotypic values based on all available markers across the entire genome [15], which achieves higher accuracy by considering small effects. Since this seminal paper, a number of predictive models have been developed, including statistical models and machine learning methods[16]. According to the type of regression functions, existing statistical methods for GS mainly fall into two categories: linear and non-linear semi-parametric. For the linear model, the phenotypic data is predicted as the summation of marker effects derived from a parametric linear regression. Because the markers incorporated in the regression outnumber the sample size enormously, a shrinkage estimation procedure is needed, depending on the types of which the linear model can be further classified into penalized methods, such as the most frequently-used ridge regression best linear unbiased prediction (RR-BLUP), and the Bayesian ones, including Bayesian LASSO, BayesA and BayesB. In contrast to linear models, non-linear semi-parametric models, such as reproducing kernel Hilbert spaces (RKHS)[17], are capable of capturing non-additive effects. A number of sophisticated machine learning tools such as random forest (RF)[18] and supporting vector regression (SVR)[19] have been applied in genome-based phenotype prediction [20–22], for their ability to recognize non-linear pattern between markers and phenotypes for robust and higher performance. GS has been applied to a variety of species, including livestock, human [23–28] and plant species [29–38], and the predictability of various models on complex traits have been addressed in maize [37, 39, 40], wheat [41, 42], sugarcane[35], dairy and beef bulls [21, 43, 44], rice [45], Arabidopsis thaliana [46], pine [47] and mice [48]. However, to our best knowledge, diverse GS algorithms have never been evaluated in *B*. *napus*.

In this study, we employed an unbiased and high-density genotyping platform on a relatively large dataset (including 1674 SNPs genotyped from 190 DH lines of cross of Tapidor×Ningyou7). Various types of GS models (linear models with penalized or Bayesian shrinkage paradigm, one semiparametric model and three machine learning methods) were applied to our data to evaluate the practicability of genome prediction of FT in *B*. *napus*, and assessment of their predictive ability. RF and Multivariate Adaptive Regression Spline Models (MARS)[49] were subsequently applied to the estimated breeding values of FT to unravel the genetic basis (including epistasis) of FT. RF is robust to outliers and can handle interactions, and the SNPs mapped by RF are good candidates for epistasis detection [50]. To demonstrate the validity of the detected SNPs, SNPs that were mapped to previously discovered QTLs, along with the genes tagged by the associated SNPs, were searched and compared with curated FT genes. Finally, to comprehensively understand FT related biological processes and functions, a function analysis was performed on the candidate genes. To our best knowledge, this is the first large-scale mapping of FT related loci via high-throughput technology, and first evaluation of GS models in *B*. *napus*. These findings would facilitate the development of breeding lines with superior flowering time in Chinese ecological conditions.

## Materials and Methods

### Genotypic and phenotypic data collection

The TN DH mapping population generated from a cross of Tapidor×Ningyou7 [51] was used for genotype detection. One hundred and ninety DH lines were genotyped on an Illumina customized Infinium platform which includes 5306 probes. No specific permits were required for the described field studies. No specific permissions were required for these locations/activities, the location is not privately-owned or protected in any way, the field studies did not involve endangered or protected species. A total of 1674 polymorphic SNPs were clustered using Genomestudio software. The genotype of each SNP was scored according to inhertitence from each parent (‘A’ represents ‘Tapidor’ and ‘B’ is denoted for ‘Ningyou7’). Before genomic selection analysis, all the samples and SNPs were subjected to a series of quality control procedures. First of all, samples with at least 20% of SNP uncharacterized were eliminated from the datasets, resulting in 182 lines left. Secondly, SNPs that could not be established in more than 10% of samples were discarded. Finally, SNPs with rare alleles (minor allele frequency < 0.05) were excluded from the study, leaving 1,248 SNPs for the subsequent analysis. The phenotypic data of FT in the TN DH population was collected from 10 natural environments, at 3 different regions, Wuhan (E114°19´/N 30°5´, South), in Hubei province for 4 years, Dali (E109°3´/N 34°5´, North), in Shanxi province for 4 years (year 2002–2003, 2003–2004, 2004–2005, and 2005–2006), and Hangzhou, in Zhejiang province (E120°12´/ N30°16´, East) for 2 years (year 2006–2007), over a period of 4 years as described by Long et al. [3]. The linkage disequilibrium (r^2^) between SNPs was calculated and visualized via the R package ‘LDheatmap’.

### Genomic selection models

Eight genomic prediction models of diverse types were used and compared in *B*. *napus*, including two frequentist based methods: ridge regression best linear unbiased prediction (RR-BLUP)[34] and reproducing kernel Hilbert spaces (RKHS)[17]; three Bayesian methods: Bayesian LASSO [52], BayesA and BayesB [15]; and two machine learning methods: random forest (RF) [18] and supporting vector regression (SVR) [19]. The details of the models have been reviewed in [16]. Briefly, based on how the relationship between markers and phenotype is modeled, the statistical models can be categorized as linear and non-linear model.

For linear models, the phenotypic value of the *i* ^*th*^ line of the population in a single environment is the regression of markers across the genome below:

$$y_{i}=\mu +\sum_{j=1}^{p}\beta_{j}x_{ij}+\varepsilon_{i},$$ (1)

where y_i_ is the phenotype, μ is the intercept, x_ij_ is the genotype of the j^th^ maker of the i^th^ line (coded as −1 and 1 for genotype inherit from Tapidor or Ningyou7 respectively), β_j_ is the regression coefficient of marker *j* and ε_i_ is the error term. To estimate the parameters, the most popular way is to minimize the residual sum of squares:

$$RSS=\sum_{i=1}^{n}{\left(y_{i}-\mu -\sum_{j=1}^{p}\beta_{j}x_{ij}\right)}^{2}$$

For GS, the markers (p) usually largely outnumber the lines (n), which would bring in the curse of dimension. In RR-BLUP, an L2-norm regularization term is introduced as a tradeoff between the complexity of the model and the fitness to the training data. The loss function is denoted as:$L=RSS+\lambda \sum_{j=1}^{p}\beta_{j}^{2}$, where λ is the regularization parameter. RR-BLUP shrinks all the markers to the same extent, regardless of the effect size of the markers. In contrast, the Bayesian methods perform differential shrinkage over markers. These Bayesian shrinkage estimations methods differed in the prior distribution put on the markers. Bayesian LASSO[52] (BL) assigns a double exponential (DE) distribution conditioned on λ to all marker effects. BL is the original LASSO in Bayesian context, using an L1-norm penalty, and the loss function is:$L=RSS+\lambda \sum_{j=1}^{p}\left|\beta_{j}\right|$. Unlike RR-BLUP, BL puts large shrinkage on small effects, and small shrinkage on large effects. BayesA assumes the marker effects are sampled from a scaled t-distribution; and BayesB utilizes a mixture prior density, assuming a proportion (π) of markers have zero effects, while the rest of the markers follow the prior distribution used in BayesA. For BayesA and BL, all the markers are assumed to have some effects, a few with large effects and many with small effects; while for BayesB, many makers are presumed to have zero effects and a few markers have large effects.

Unlike multiple linear regression models, semi-parametric and non-parametric models are capable of accommodating non-additive genetic effects on phenotypes, such as epistasis interactions. RKHS[17] models non-linear relationships between markers and phenotype in a high-dimension feature space. Here we used a Gaussian kernel:$K_{ij}=exp\left[-{\left(\frac{D_{ij}}{\theta }\right)}^{2}\right]$, where D_ij_ is the Euclidean distance between line i and j, and θ controls the decay rates.

SVR can be viewed as a specific learning process of RKHS. In this study, the ‘ε-insensitive’ SVR was used, which only considers absolute values of residuals larger than ε.

RF[18] is an ensemble of classification or regression decision trees, built on randomization of the sample in the training set and each splitting node on the trees is selected from a random subset of variables. In this way, all markers and all possible interactions are taken into account, which hold the promise of capture a large number of genetic interactions [53].

All the analysis was performed in R statistical computing environment. RR-BLUP and RKHS were implemented in the ‘rrBLUP’ package [54]. ‘BGLR’ package was used to perform all three the Bayesian models[55]. RF was carried out via the ‘randomForest’ packages[56]. The supporting vector regression (SVR) was implemented in the ‘e1071’ package[57]. Two kernels were tested (Gaussian and linear). A grid search was used for tuning the parameters. Due to the computational intensity of SVR a tuning process was used.

### Predicting breeding values

To verify the feasibility of our chip data in genomic selection of candidates in *B*. *napus*, we first applied the two most frequently used methods RR-BLUP and RKHS to the FT trait in *B*. *napus*, collected from ten environments. For both models, genetic factors and errors were taken as random effects, and year-site combination as covariates. After that, Genome Breeding values under each environment were predicted and compared via all the statistical and machine-learning models mentioned above.

The performances of predictive models were evaluated using a 10-fold cross-validation (CV) scheme. Namely, the lines were divided into 10 disjoint subsets of equal sizes, and each subset was sequentially taken as a testing-set while the remaining ones were used to train the predictive model using different methods. This CV process was repeated ten times and the mean Pearson correlation between the observed and predicted trait value were calculated as the accuracy. For each run of CV, the same training and test set were used for all the models to guarantee a fair comparison.

The overall genomic estimated breeding values (GEBVs) were predicted using RKHS with the genetic effect and error as random effects and site-year combination as a covariate implemented in the R package ‘rrBLUP’. The site-specific (north, south and east) breeding values were also measured respectively, with year as a covariate included.

### Selection of Associated SNPs

Unlike conventional QTL approaches, RF scores the importance of SNPs, considering multi-loci and the interactions among them, so it’s capable of discovering SNPs with small effects and with strong epistasis effects. For each RF model, at each split one third of the SNPs were tried and 1000 trees were grown. The SNPs were ranked in descending order, based on average importance scores obtained from 20 runs. The SNPs highly related with FT genetic effects were selected in a recursive inclusion process. First, the top 5% of important SNPs were used, and another 5% SNPs were added in the model iteratively. For each model, mean square error (MSE) was recorded. This process was repeated 20 times, and the set of SNPs with minimal MSE was selected [58]. As seen in a previous study [59], random forest tends to overweigh correlated predictor variables, which is common for genotyping data. To avoid the bias towards clustered SNPs, a pruned set of SNPs (r^2^ < 0.7) was investigated.

The directions of SNPs on flowering time were then examined. The average flowering time (in days) of lines with either genotype (A or B) at every single identified SNP were calculated respectively and compared in each environment. The allelic direction of a SNP is considered consistent when the genotype representing early blossom is the same in the environments that it is significant.

### Epistasis effects mapping

Similar to the procedure used in [50], the SNPs identified based on RF were then input into a Multivariate Adaptive Regression Spline (MARS) Model to identify epistasis effects. MARS has been proven powerful in detecting SNP-SNP interactions[49], but its efficacy could be limited by a large number of irrelavent SNPs. And RF is a useful tool in selection of associated SNPs, taking the interactions among SNPs into account. Therefore, the integration of these two methods would provide more advantages[50]. We used the ‘earth’ package to apply MARS. Ten runs of 10-fold cross-validation were used to determine the interactions.

### Annotation of SNPs

Due to the lack of available annotations for the *B*. *napus* genome, we adopted the function information of the homologues from well-annotated genomes like *Arabidopsis thaliana* to better understand the associated SNPs. First of all, BLASTX analysis of the probes of the SNPs against RefSeq proteins of *Arabidopsis thaliana* was performed, and only the hits with expected values < 1×10^−6^ were retained. Among 1, 248 SNPs used in this study, 566 SNPs could be annotated in this manner. A large number of SNPs are located in the intergenic regions, so their functional information would not be identified by BLASTX against coding genes. Due to linkage disequilibrium, one SNP tags multiple genes in a region and therefore the flanking genes of a significant SNP may also be the candidates associated with FT. To further pinpoint the candidate genes, we mapped the probes to the *Brassica rapa* genome using bowtie2 [60], which is an efficient and widely used aligner to map sequencing reads to the reference genome, and has no upper limit in read length. The probes were inputted into bowtie2 as reads. The *B*. *rapa* genome sequence was downloaded from Brassica database (BRAD, http://brassicadb.org/brad/). The results showed that 70.97% of the 682 probes left map at least once on the *B*. *rapa* genome and 279 SNPs have genes in vicinity (1 kb). The corresponding orthologs were searched among RefSeq proteins *Arabidopsis thaliana* via BLASTP (expect values < 1×10^−6^). Finally, the genes represented by the rest of the 403 SNPs were searched by BLASTX to the NCBI non-redundant protein sequences (nr), and hits with expected values < 1×10^−6^ were retained. In this stage 40 SNPs were annotated.

### Known flowering time genes/proteins

The list of curated flowering time related proteins was gathered from multiple sources. We first searched the NCBI protein database with ‘flowering time’ as query. The flower gene in *B*. *rapa* was downloaded at http://brassicadb.org/brad/flowerGene.php. Genes of ‘Flowering time pathway’ were downloaded from the wikiPathways website at www.wikipathways.org (Pathway:WP2312).

### Function analysis

DAVID (http://david.abcc.ncifcrf.gov/home.jsp) was used for functional enrichment analysis. The functional clusters of significant Gene Ontology terms among candidate genes were surveyed via DAVID Functional Annotation Clustering Tools[61]. And GO terms at level 5 were used to find more specific functional annotations of candidate genes.

### Mapping detected SNPs to previously detected QTLs

Primers were designed to some SNPs, based on the resource sequences. PCR amplification was done in the TN DH mapping population, and the SNP markers re-mapped in the TNDH linkage map. QTL mapping analysis of FT traits was performed to see whether the SNP markers were located in the QTL confidence interval. The parameters were the same as in previously described papers [3].

## Results

### Evaluation of breeding values in *B*. *napus*

To verify that our genotype data is sufficient for genomic prediction of the FT trait in *B*. *napus* and to evaluate the genomic prediction models two conventional genomic prediction models RR-BLUP and RKHS were first applied to SNP chip and FT trait data collected across ten environments. The former method only considers additive genetic effects and the latter one is capable of capturing non-additive effects. In both models, the genetic effects and errors were taken as random effects with the site-year combinations as covariates. Ten runs of 10-fold cross-validation scheme were utilized to evaluate the overall performance (see methods). The accuracy is calculated as the mean Pearson correlation coefficients between the predicted trait values and average FT across all the environments. Relatively high accuracies were achieved for both methods (0.737 and 0.760 respectively), and the better performance of RKHS indicated that non-additive effects exist.

**Fig 1:**
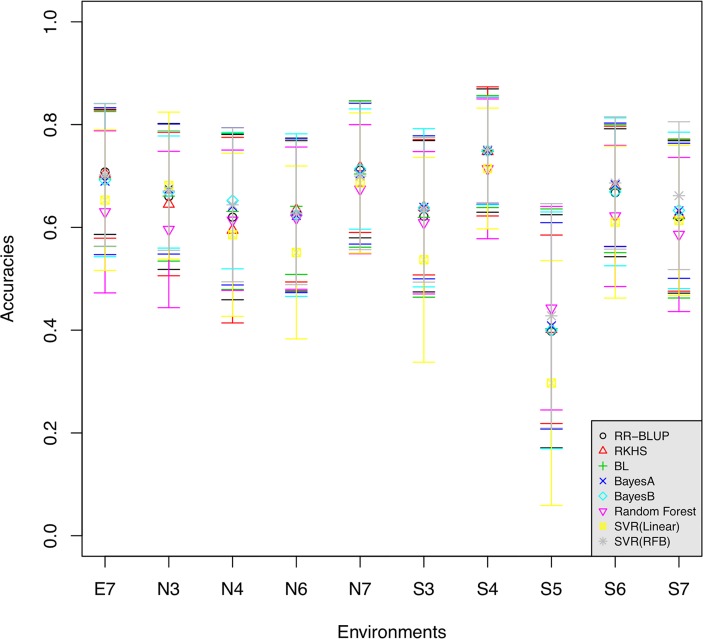
The comparison of the accuracies achieved by eight exiting genome selection models in each of the environment. Each node indicates mean accuracies of 10 runs of 10-fold cross-validation, and the ranges stand for ± standard deviation. The prediction accuracy was calculated as the Pearson correlation coefficient of predicted and observed FT.

We further made a comprehensive evaluation of eight genomic prediction models, including five statistical algorithms (including RR-BLUP, RKHS and Bayesian methods) and two powerful machine-learning methods. To simplify the comparison, all the GS models were tested in each of the environments via 10-fold cross-validation, and the mean Pearson correlation coefficients of predicted and observed FT were calculated as prediction accuracy. All of the methods achieved relatively high and comparable accuracies (0.297~0.751) (Table 1). The two frequencist methods (RR-BLUP and RKHS) performed nearly equivalently, with accuracies as 0.638 and 0.639 respectively. Among linear models, the models with Bayesian shrinkage estimation (average accuracies of 0.639, 0.645 and 0.644 respectively for BL, BayesA and BayesB) were slightly better than the penalized RR-BLUP. For machine learning methods, SVR with Gaussian kernel was somewhat superior, with an average accuracy of 0.651 and performing the best in three environments; while SVR with linear kernel was relatively inferior (0.593). It's also worth noting that the performances varied in different environments and the environments influence the GS model similarly. Namely, all the methods achieved their best accuracies in S4 (0.714~0.751) and the worst accuracies in S5 (0.297~ 0.443), and the performance in other environments altered accordingly (Fig. 1). Our results showed that no GS models fitted all the environments and each model generated the best accuracy in at least one environment, whilst SVR with Gaussian kernel was advantageous in three environments. It’s interesting to see that the inferior model SVR with linear was the optimal method in S4.

**Table 1:** The performance of various genome-based trait prediction methods applied to flowering time in multiple environments.

Environments	RR-BLUP	RKHS	Bayesian LASSO	BayesA	BayesB	Random Forest	SVM (linear kernel)	SVM(Gaussian kernel)
E7	**0.708**	0.704	0.694	0.690	0.692	0.630	0.653	0.702
N3	0.660	0.645	0.661	0.675	0.669	0.596	**0.681**	0.671
N4	0.620	0.595	0.631	0.641	**0.652**	0.614	0.586	0.644
N6	0.623	0.634	**0.641**	0.623	0.624	0.618	0.551	0.628
N7	0.711	**0.716**	0.704	0.705	0.713	0.674	0.686	0.700
S3	0.622	**0.639**	0.620	**0.639**	0.638	0.609	0.537	0.634
S4	0.750	0.748	0.748	0.749	0.749	0.714	0.715	**0.751**
S5	0.398	0.402	0.403	0.408	0.400	**0.443**	0.297	0.428
S6	0.667	0.680	0.675	0.683	0.669	0.622	0.610	**0.686**
S7	0.620	0.624	0.617	0.632	0.633	0.586	0.614	**0.662**
Average	0.638	0.639	0.639	0.645	0.644	0.611	0.593	0.651

The best prediction model for each environment in the data set is in bold. The performance was evaluated via 10 runs of 10-fold cross-validation and the prediction accuracy was the mean Pearson correlation coefficients of predicted and observed FT.

Finally, for further association study, genomic estimated breeding values (GEBVs) were predicted for each geographic region using RKHS with year as a covariate, respectively. RKHS implemented in the R package ‘rrBLUP’ is capable of handling multiple environments.

### SNPs associated with Flowering time

**Fig 2:**
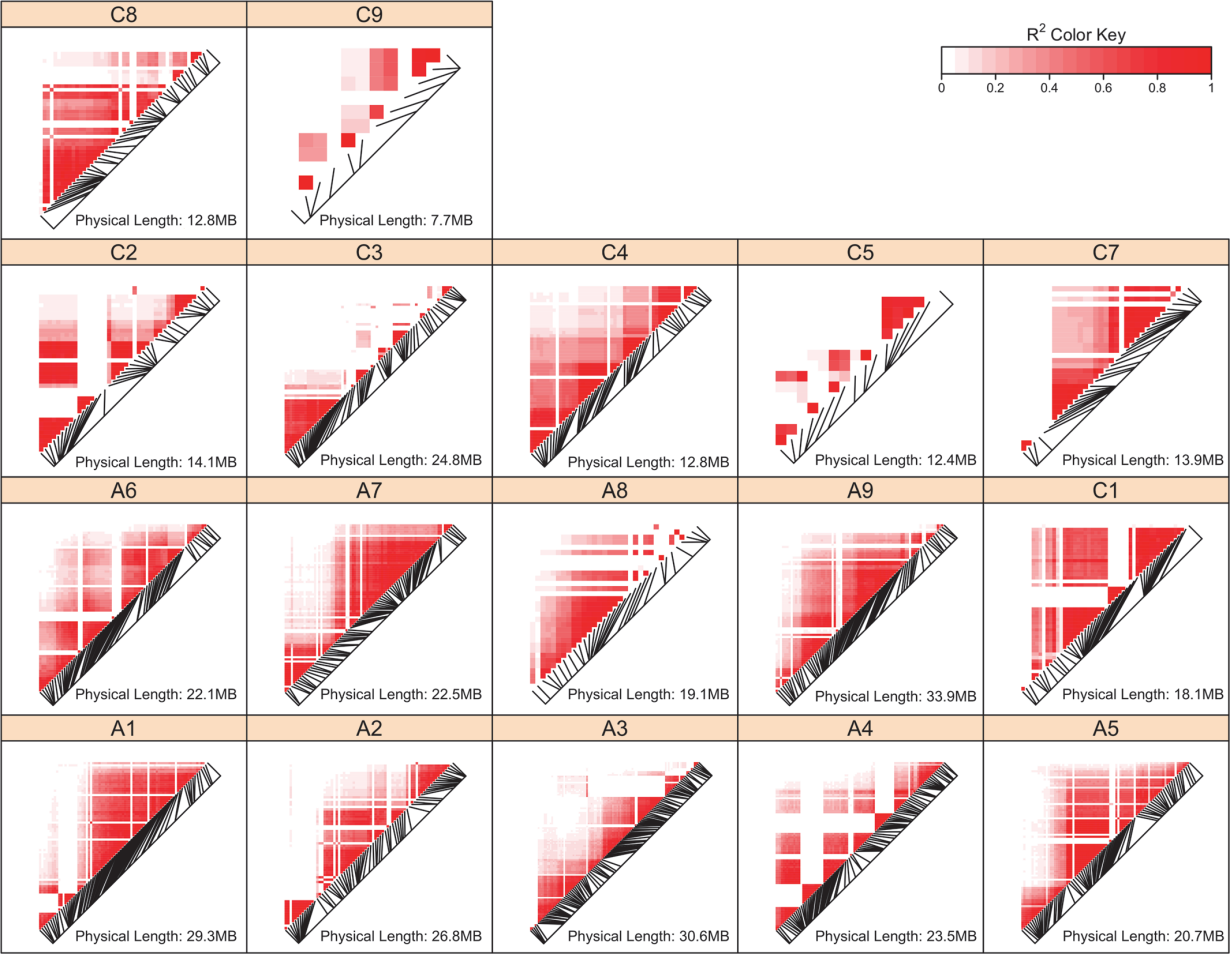
Pairwise linkage disequilibrium (r2) of genomic markers in each chromosome.

SNPs that contribute to the GEBVs of FT trait in *B*. *napus* were detected by random forest, which is capable of capturing interactions and scoring the importance of the SNPs and has been used in feature selections. To reduce spurious associations due to accidental factors, the associations of SNPs with estimated breeding values (EBVs) derived from each geographic site trait values were tested respectively, instead of FT trait values of each site-year combination. The observed strong correlations among SNPs (Fig. 2) would result in preference over correlated SNPs. Thus, to eliminate this bias, a set of pruned SNPs was studied. In total, 437 SNPs represented by 47 tag SNPs in the pruned SNP set were detected across three geographic sites (S1 Table), and the number of associated SNPs varied in different sites, for example, 184 SNPs in north, 279 in south and 344 in east, surrogated by 24, 32 and 32 tag SNPs respectively (S2 Table).

A large number of SNPs detected in one site were found to be significant in another site. As shown in S1 Fig., about 29.5% of the detected SNPs were found to be replicable across all sites, while 44.6% of SNPs were identified in only in one geographic condition, which indicated that the environmental conditions could influence FT traits. Among the shared SNPs, UQnapus0669 was the most prominent one, ranking the first in all three sites. Searching the genes bearing or surrounding UQnapus0669 and SNPs in its proxy found two curated FT genes. UQnapus0669 was located in a gene region with similar sequence with *CAM4* ‘calmodullin 4’, involved in the flowering time pathway (wikiPathway: WP2312) and UQnapus0104 represented by UQnapus0669 resided in a homologue of another known *FT* gene, *AP1* ‘Floral homeotic protein’. Some other FT genes were also found tagged by the detected SNPs (Table 2). For instance, *PHYE* ‘phytochrome E’ (a homolog of ‘phytochrome B’ in *Aquilegia formosa*), *AT1G68920* ‘transcription factor bHLH49’ (a homolog of established FT gene *CIB5*) and *GRF8* ‘14-3-3-like protein GF14 kappa’ (participating in flowering time pathway) were tagged by three site sharing SNPs (UQnapus4804, UQnapus1445 and UQnapus5584 respectively). It’s interesting to see that two known FT genes, *AGL24* ‘MADS-box protein’ and *AT2G01820* ‘receptor-like kinase TMK3’ (a homolog of flowering time protein CAM31941 in *Lolium perenne*) were only tagged by two eastern site-specific SNPs.

**Table 2:** Associated SNPs tagging known FT genes.

SNP	Chromosome	Coordinate	Homologs	Comments
**UQnapus0104**	C5	7700765	ref|NP_177074.1| Floral homeotic protein APETALA 1 [Arabidopsis thaliana]	*AP1*, Known FT gene, contain MADS-box, shared in all sites
**UQnapus0669**	unassigned C genome	270314394	ref|NP_176814.1| calmodulin 4 [Arabidopsis thaliana]	*CAM4*, involved in flowering time pathway, (WikiPathways: WP2312), shared in all sites
**UQnapus4804**	A1	7194790	ref|NP_193547.4| phytochrome E [Arabidopsis thaliana]	*PHYE*, a homolog of a flowering time gene ‘phytochrome B’ in *Aquilegia formosa*, shared in all sites
**UQnapus1109**	A1	4708098	ref|NP_195034.2| AGC (cAMP-dependent, cGMP-dependent and protein kinase C) kinase family protein [Arabidopsis thaliana]	*AT4G33080*, homolog of flowering locus ‘AT2G20470-like kinase’ in *Capsella bursa-pastoris*
**UQnapus1445**	A2	17249324	ref|NP_177058.1| transcription factor bHLH49 [Arabidopsis thaliana]	*AT1G68920*, also a homolog of known FT gene *CIB5* in *Arabidopsis thaliana*, shared in all sites
**UQnapus5584**	A3	29135636	ref|NP_001190621.1| 14-3-3-like protein GF14 kappa [Arabidopsis thaliana]	*GRF8*, involved in flowering time pathway, (WikiPathways: WP2312), shared in all sites
**UQnapus0974**	unassigned C genome	349256190	ref|NP_568567.1| Dof zinc finger protein DOF5.2 [Arabidopsis thaliana]	*CDF2*, involved in flowering time pathway, (WikiPathways: WP2312)
**UQnapus4390**	C7	18411249	ref|NP_194185.1| MADS-box protein AGL24 [Arabidopsis thaliana]	*AGL24*, involved in floral whorl development, only in east
**UQnapus0057**	A3	20996981	-	Located near *Bra000393*, a homolog of *AGL20*, which is a flower gene
**UQnapus0054**	A1	2984710	-	Located near *Bra029305*, a homolog of *LFY*, which is a flower gene
**UQnapus5172**	A6	22522149	ref|NP_178291.1| receptor-like kinase TMK3 [Arabidopsis thaliana]	*AT2G01820*, a homolog of flowering time protein CAM31941 in *Lolium perenne*, only detected in east

**Fig 3:**
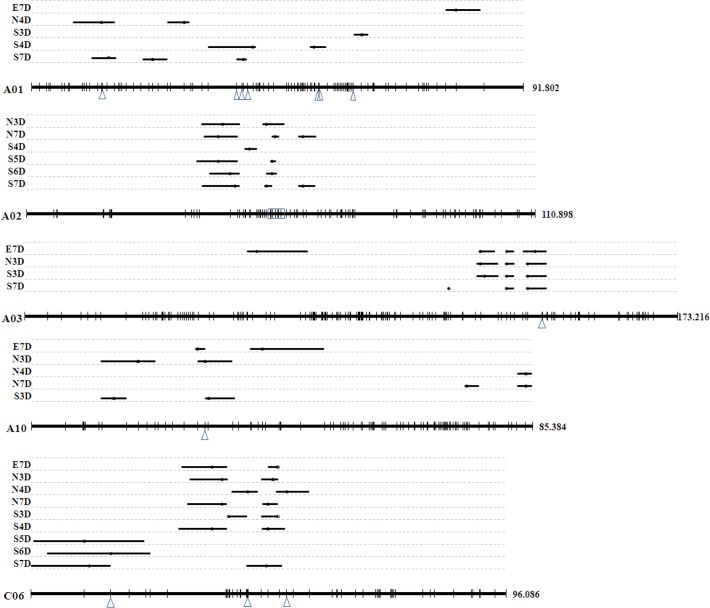
SNPs located in previously found QTLs. Five linkage groups were showed with the lines, and the black short lines represented the QTLs. The blue triangles showed the SNPs located in the confidence interval of QTLs.

Besides association analysis, the 1674 polymorphic SNP markers were combined with some common SSR markers to construct a linkage map (S3 Table), and FT associated SNPs were included in the linkage map. It was found that 31 significant SNPs were mapped in the linkage map. Further QTL mapping showed that there were 19 flowering time related QTL detected. Among the QTLs, 6 QTLs were detected for North environment, 12 QTLs were detected for South environment, and 1 for East environment. Comparison of the FT associated SNPs with mapping QTLs, found that 23 SNPs could be detected in both QTL mapping and our method (Fig. 3), which meant that these SNPs were real genetic loci controlling flowering time.

**Fig 4:**
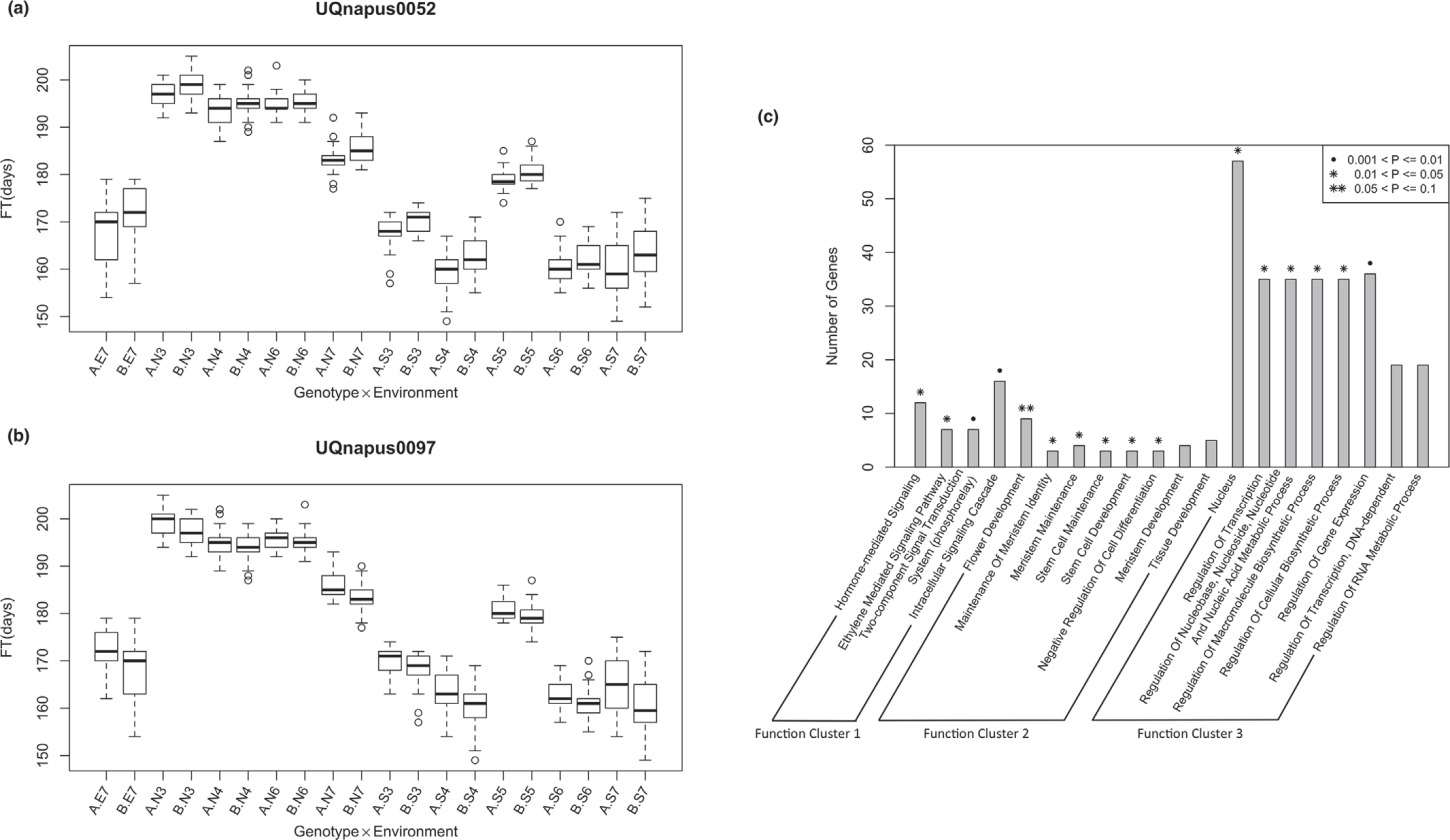
Illustration of genotype effects of associated SNPs on FT and functional clusters of genes tagged by detected SNPs. (**a)** Samples with allele ‘A’ at UQnapus0052 tend to blossom earlier than the ones with allele ‘B’ (with *t test* P-value from 3.47 ×10^−9^ to 3.95 ×10^−1^). (**b)** while at UQnapus0097, lines with allele ‘B’ are more likely to flower sooner (with *t test* P-value from 1.23 ×10^−7^ to 1.44 ×10^−1^). **(c)** Functional clusters with enrichment score > 1.3 (corresponding to p value of 0.05)

We then examined whether the genotypes of the associated SNPs show the same allelic direction across environments, i.e. lines that flower earlier have the same allele at a specific SNP under all the environmental conditions. Among 437 identified SNPs, 72.5% (317 SNPs) have the consistent early blossom genotypes. For instance, samples that had inherited the allele from ‘Tapidor’ at UQnapus0052 tend to blossom earlier than the ones with allele from ‘Ningyou7’ in all ten environments (Fig. 4A), which is opposed to UQnapus0097 (Fig. 4B). For the majority (259) of the consistent SNPs, the allele ‘B’ from Ningyou7 is associated with early flowering.

### Functional enrichment analysis for candidate genes

To further dissect the function of the significant SNPs, we obtained the genes bearing or near the associated SNPs by searching for the probes’ homolog or the genes within a 1 Kb window around the mapped probe on *B*. *rapa* genome as candidate genes (see material and methods). In total, 285 candidate genes were observed. The functional enrichment analysis on the candidate genes was performed via DAVID functional annotation clustering tool[61]. Three functional groups were found significant with enrichment scores > 1.3 (equivalent to p-value 0.05) (Fig. 4C, for more details see S4 Table). The result shows that, the genes are mainly involved in signal transduction, such as hormone-mediated signaling (GO:0009755, fold-enrichment of 2.27, and Fisher’s exact test P = 1.45×10^−2^); tissue developments, especially flower development (GO:0009908, fold-enrichment of 3.16, P = 7.45×10^−3^); and regulation of expression (such as GO:0045449~regulation of transcription, fold-enrichment of 1.41 and P = 2.79×10^−2^); and these three functional clusters comprise about 5.6%, 3.5% and 21.8% of the candidate genes respectively. To further characterize the functional differences between the SNPs detected common to all geographic sites and specific to only one or two regions, we further checked the function annotation of genes tagged by SNPs detected in all three regions and the ones only reproducible in one or two regions respectively by using DAVID Functional Annotation Table Tool, and again performed function enrichment analysis on these two sets of genes (S5 Table). For region-common genes, three of them (*AP1* tagged by UQnapus0104, *AT5G10510* represented by UQnapus1789, *AT4G29010* tagged by UQnapus5033) were annotated with GO term GO:0009908~flower development. The top two groups, involved in mRNA processing and regulation of transcriptions, however neither of these functional clusters were found significant (enrichment score > 1.3). As for region-specific candidate genes, genes of leaf morphogenesis and phyllome development are enriched (with enrichment score of 1.45).

### Epistasis effects on FT

The epistasis interactions between SNPs associated with flowering time were detected for each geographic site by MARS[50]. To facilitate computation, only tag markers in the pruned set that were identified in the previous session were analyzed and only 2-order interactions were examined. In total, 9, 17 and 16 pairs of SNPs were detected with epistasis effects on FT in north, south and east environment, respectively, and SNPs tagging known FT genes were detected interacting with each other (S6 Table). Contrary to the extensive overlap among the markers detected in each site, none of SNP pairs were shared in all of the sites, and only one pair ‘UQnapus0669 × UQnapus1545’ was replicated in two sites (north and south). As shown in the previous section, UQnapus0669 is the most significant marker for all three sites representing two known FT genes, while UQnapus1545 and its representing the markers were tagging *SKIP1* ‘F-box protein SKIP1’ and *AT1G53100* ‘core-2/I-branching beta-1,6-N-acetylglucosaminyltransferase-like protein’. UQnapus0669 was revealed to interact with SNPs that were also in/near known FT genes. For instance, the interaction of UQnapus0669 and UQnapus3878 was found in the north, the latter of which represented Dof zinc finger protein *DOF5*.*2*; UQnapus0669 also interacted with UQnapus5033, a proxy for *PHYE* ‘phytochrome E’; the epistasis effects of UQnapus0669 and UQnapus4810 was identified in the east, representing *AGL24* ‘MADS-box protein’. Two other pairs of known FT genes showed epistasis effects in east, such as *AT1G68920* ‘transcription factor bHLH49’ and *PHYE* ‘phytochrome E’, tagged by UQnapus1450 and UQnapus5033, and *LFY* and *GRF8* ‘14-3-3-like protein GF14 kappa’, represented by UQnapus0238 and UQnapus1789.

## Discussion

We assessed the predictability of various types of GS models, including statistical models (linear models with penalized or Bayesian estimation), semi-parametric model and machine learning methods. Our results showed that no apparent divergence of accuracies was observed among these GS models, which agreed with previous studies [21, 35, 46]; while the SVR with Gaussian kernel performed better to some extent, confirming the previous conclusions [21, 22]. Among the linear models, the models with Bayesian shrinkage estimation (BL, BayesA and BayesB) were better than penalized regression RR-BLUP, accordant with [62]. RR-BLUP shrinks all the marker effects homogeneously; while the Bayesian methods allow different levels of shrinkage over marker effects by allowing variance of its own, which is more realistic. Although BL was supposed to outperform RR-BLUP, for our results, no increase of accuracy was observed in our results, probably due to the large LD span in the *B*. *napus* genome. Semi-parametric model RKHS were presumed to perform better by taking cryptic non-additive genetic effects in consideration; however, in our results RKHS did not outperform linear models. The possible reason is that the marker density is relatively low, despite of high accuracies achieved. With a higher density panel, the semi-parametric methods are more accurate [31]. SVR with Gaussian kernel seemed more appealing with the highest average accuracy, as in [21].

Previous studies showed genetics and environment interaction took up a large proportion of phenotypic variation [3]. And the large influence was observed on the predictability of GS models, and the patterns of accuracies were similar for different models. None of the models perform the best across all the environments, except the SVR with Gaussian kernel was somewhat superior (performing the best in multiple environments). It’s interesting to see that RF has the best predictability in S4. It’s probably because the extreme climate condition leads to a large number and even high order of marker interactions, which can be captured by RF.

To enhance the GS accuracies, a higher marker density would be used. Although it would bring troubles to linear models, due to the colinearity among markers, it would be beneficial for non-linear models [37]. Another possible improvement would be using whole genome sequence data. Recent studies showed that whole genome sequence data holds the promise to improve the genomic prediction [63, 64], for including causal variants. As shown in the results, the performance of GS models varied across the environments, therefore borrowing information from similar environment is another potential improvement [37].

QTL mapping based on the EBVs derived from the replicates would remove some spurious signals due to accidental factors. As showed in the results, there were a large proportion of overlaps among three sites, while among the SNPs detected in each environment by univariate method only ten were common for all ten environments (data not shown). Compared with 46 SNP loci detected by our method, the efficiency of QTLs mapping was lower[3]. Some known FT genes were tagged by our detected SNPs, demonstrating the practicability of SNP genotyping data in QTL mapping. In fact, we used three mapped SNPs to do confirmation work by transferring them to common markers. The result showed that the transferred SNP markers, UQnapus5530, UQnapus1399 in A2 linkage group and UQnapus5751 in A10 linkage group could be re-mapped in the linkage map and located in the QTL confident interval (S2 Fig.). That mean the SNPs screened by our method were real ones. Due to the lack of thorough annotation of *B*. *napus*, we selected the FT candidate genes by mapping the probes to well-annotated genomes such as *A*. *thaliana* and *B*. *rapa*. And among these candidate genes, some genes are established FT genes (such as *AP1*, *CAM4 and GRF8*) and the others are the homologs of reported FT genes in other species (like *PHYE*, *AT2G01820 and AT4G33080*), and the results implicated these genes’ involvement in flowering process. It’s intriguing to see that now all the curated FT genes (or homologs) are reproducible under all environmental conditions, such as *LFY* and *CDF2*. Especially, *AGL24* and *AT2G01820* are solely detected in east site, indicating flowering is susceptible to geographic and climate conditions. And according to functional analysis, three functional clusters were found significantly enriched among candidate genes. Although, a number of candidate genes would be missed since only small surrounding regions of SNPs were considered, the well-established FT related biological processes (such as flower development and meristem development) were reproduced, indicating our method works for the unannotated genome. And among these functional groups, transcription regulation took up a largest amount of detected candidate genes (28.1%), showing the association of essential function for maintenance with flowering. Moreover, the overrepresented signaling transduction annotation among candidate genes implies flowering is influenced by multiple factors. We tried to address different characteristics between region-common and region-specific SNPs. Although no significant functional clusters were found among region-common genes, the top two groups, involved in mRNA processing and regulation of transcriptions, somewhat suggest that the common genes mainly exert essential function for maintenance. On the contrary, region-specific candidate genes are enriched for genes of phyllome development significantly and flower development marginally, implying that the blossom process is more likely affected by environment. Almost no overlap was found among epistasis interactions among three regions, indicating epistasis effects are more prone to be impacted by environments.

## Supporting Information

S1 Fig Comparison of associated SNPs across three geographic sites. (TIF)

S2 Fig The graph of QTL mapping results of A2 and A10 linkage group. The arrows showed that the three re-mapped SNP markers in the linkage groups.
(TIF)

S1 Table Annotations of SNPs associated with the flowering time trait. (DOCX)

S2 Table The associated tag SNPs and their representing SNPs in each of the geographic sites. (DOCX)

S3 Table SNPs in the known FT related QTLs. (DOCX)

S4 Table Functional clusters of genes tagged by associated SNPs with enrichment score > 1.3 (corresponding to p value of 0.05). (DOCX)

S5 Table Top three functional groups of genes represented by SNPs common to all three geographic sites and specific to one or two regions respectively. (DOCX)

S6 Table All the pair of interacting SNPs detected in the epistasis analysis. (DOCX)
