# Supplementary figures and images for: Genome Wide Analysis of Flowering Time Trait in Multiple Environments via High-Throughput Genotyping Technique in Brassica napus L
Source: PLoS One. 2015 Mar 19;10(3):e0119425. doi: 10.1371/journal.pone.0119425 (PMC4366152; doi:10.1371/journal.pone.0119425)

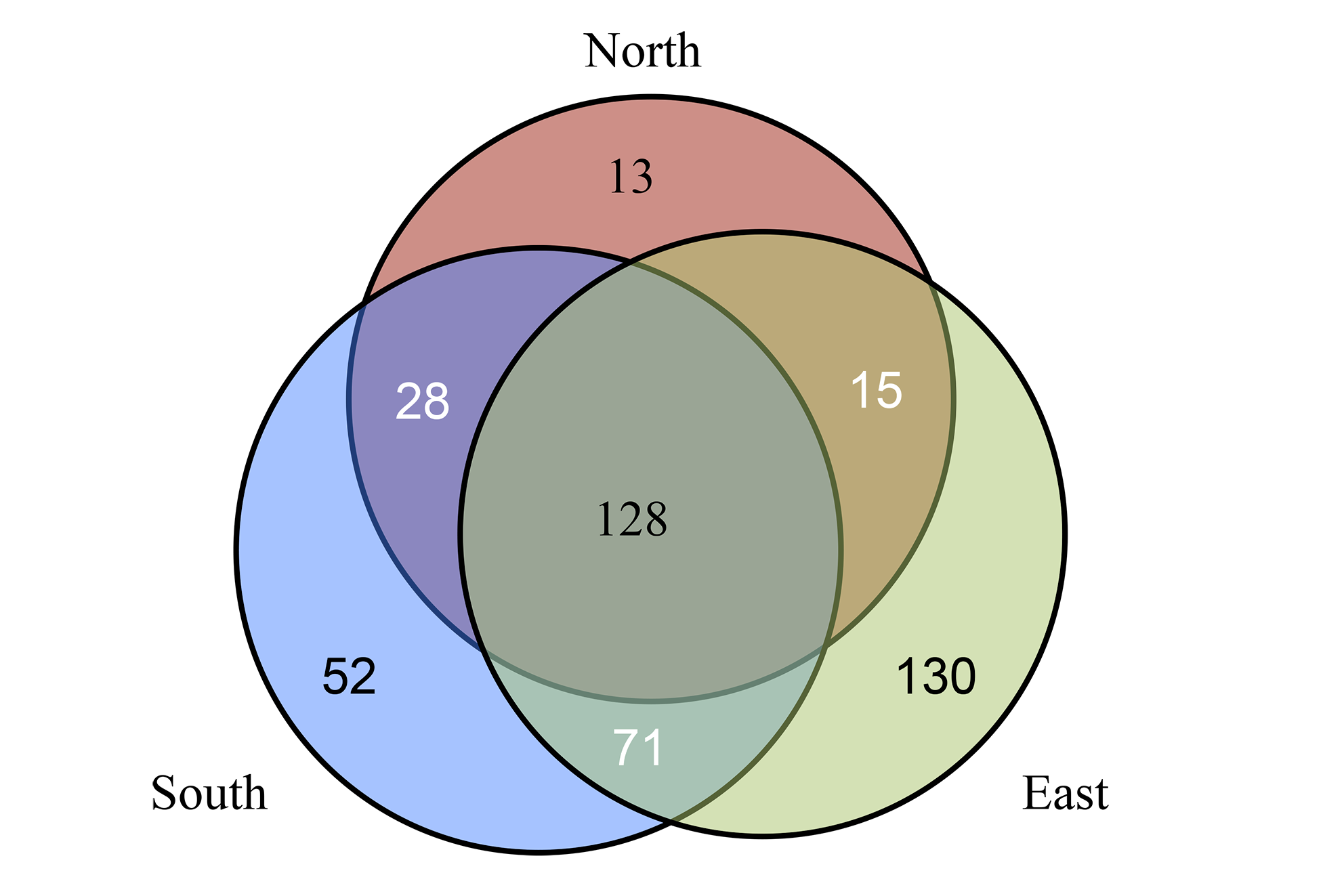

Supplement: S1 Fig — (TIF) [file pone.0119425.s001.tif]

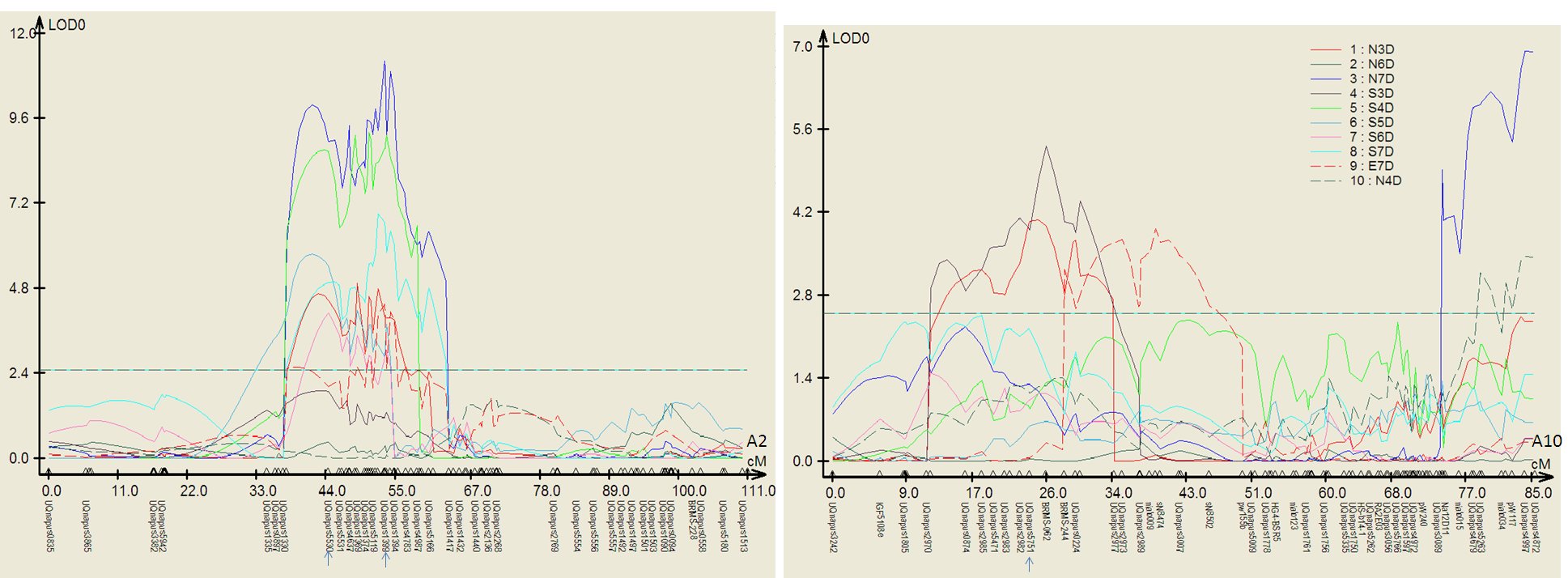

Supplement: S2 Fig — The arrows showed that the three re-mapped SNP markers in the linkage groups. (TIF) [file pone.0119425.s002.tif]
