# Supplementary material for: Genome Wide Analysis of Flowering Time Trait in Multiple Environments via High-Throughput Genotyping Technique in Brassica napus L
Source: PLoS One. 2015 Mar 19;10(3):e0119425. doi: 10.1371/journal.pone.0119425 (PMC4366152; doi:10.1371/journal.pone.0119425)
Supplement: S1 Table — (DOCX) [file pone.0119425.s003.docx]

**S1 Table .** Annotation of SNPs associated with the flowering time trait

| Locus Name | Chromosome | Coordinate | Neighboring Gene |
| --- | --- | --- | --- |
| UQnapus0052 | unassigned C genome | 161593040 | AT1G68030 |
| UQnapus0054 | 1 | 2984710 | AT5G61850 |
| UQnapus0057 | 3 | 20996981 | AT2G45660 |
| UQnapus0092 | 4 | 2764678 | AT2G36910 |
| UQnapus0097 | 2 | 10360194 |  |
| UQnapus0098 | 4 | 2767319 | AT2G36910 |
| UQnapus0104 | 15 | 7700765 | AT1G69120 |
| UQnapus0125 | 2 | 10366409 | AT5G52300 |
| UQnapus0144 | 13 | 13662850 |  |
| UQnapus0203 | unassigned A genome | 2638123 |  |
| UQnapus0224 | unassigned A genome | 3882168 | AT5G08110 |
| UQnapus0237 | unassigned A genome | 5483248 | AT3G08850 |
| UQnapus0238 | unassigned A genome | 5550956 | AT4G00040 |
| UQnapus0249 | unassigned A genome | 7289351 |  |
| UQnapus0250 | unassigned A genome | 7347985 |  |
| UQnapus0293 | unassigned A genome | 14757173 | AT1G68520 |
| UQnapus0304 | unassigned A genome | 15849982 |  |
| UQnapus0309 | unassigned A genome | 16371883 | AT4G02260 |
| UQnapus0330 | unassigned A genome | 19439477 |  |
| UQnapus0332 | unassigned A genome | 19735172 | AT4G05095 |
| UQnapus0333 | unassigned A genome | 19812258 | AT3G53630 |
| UQnapus0337 | unassigned A genome | 20261867 | AT4G11740 |
| UQnapus0341 | unassigned A genome | 21046042 | AT4G04910 |
| UQnapus0351 | unassigned A genome | 23038547 | AT1G43760 |
| UQnapus0390 | unassigned A genome | 31508049 | AT3G54710 |
| UQnapus0394 | unassigned C genome | 35766769 | AT2G02080 |
| UQnapus0406 | unassigned C genome | 46008512 |  |
| UQnapus0439 | unassigned C genome | 79573534 | AT5G52050 |
| UQnapus0532 | unassigned C genome | 160585989 |  |
| UQnapus0535 | unassigned C genome | 162567210 |  |
| UQnapus0553 | unassigned C genome | 181063042 | AT3G02850 |
| UQnapus0574 | unassigned C genome | 204740992 | AT3G21290 |
| UQnapus0594 | unassigned C genome | 222354334 |  |
| UQnapus0604 | unassigned C genome | 229573398 |  |
| UQnapus0605 | unassigned C genome | 230000907 |  |
| UQnapus0619 | unassigned C genome | 240517963 |  |
| UQnapus0624 | unassigned C genome | 245353341 |  |
| UQnapus0665 | unassigned C genome | 267078239 | AT3G47780 |
| UQnapus0669 | unassigned C genome | 270314394 | AT5G21274 |
| UQnapus0689 | unassigned C genome | 278466856 |  |
| UQnapus0701 | unassigned C genome | 284774117 |  |
| UQnapus0712 | unassigned C genome | 289949194 | AT4G29100 |
| UQnapus0743 | unassigned C genome | 302843520 | AT1G49920 |
| UQnapus0772 | unassigned C genome | 312650134 | AT3G15590 |
| UQnapus0776 | unassigned C genome | 314085828 |  |
| UQnapus0792 | unassigned C genome | 318573521 |  |
| UQnapus0806 | unassigned C genome | 322014719 |  |
| UQnapus0816 | unassigned C genome | 324547579 | AT1G43760 |
| UQnapus0827 | unassigned C genome | 327587297 | AT2G30800 |
| UQnapus0832 | unassigned C genome | 328617600 |  |
| UQnapus0860 | unassigned C genome | 334435878 |  |
| UQnapus0863 | unassigned C genome | 334923538 |  |
| UQnapus0874 | unassigned C genome | 336839575 | AT5G04990 |
| UQnapus0888 | unassigned C genome | 339107307 | AT1G48930 |
| UQnapus0895 | unassigned C genome | 340131095 | AT3G01940 |
| UQnapus0896 | unassigned C genome | 340248580 |  |
| UQnapus0907 | unassigned C genome | 342123222 | AT1G75370 |
| UQnapus0911 | unassigned C genome | 342450300 |  |
| UQnapus0920 | unassigned C genome | 343229824 |  |
| UQnapus0927 | unassigned C genome | 344061326 | AT3G45560 |
| UQnapus0969 | unassigned C genome | 348782790 | AT4G28400, AT4G28380 |
| UQnapus0974 | unassigned C genome | 349256190 | AT5G39660 |
| UQnapus0976 | unassigned C genome | 349377056 | AT4G29090 |
| UQnapus0988 | unassigned C genome | 350371317 |  |
| UQnapus1022 | unassigned C genome | 352191081 |  |
| UQnapus1026 | unassigned C genome | 352280892 | AT5G53290 |
| UQnapus1032 | unassigned C genome | 352508530 | AT1G43760 |
| UQnapus1046 | unassigned C genome | 353189826 | AT2G26570 |
| UQnapus1060 | 1 | 367907 | AT4G39780 |
| UQnapus1065 | 1 | 1032242 | AT4G36870 |
| UQnapus1070 | 1 | 1234013 | AT4G36360 |
| UQnapus1073 | 1 | 1411952 | AT4G35840 |
| UQnapus1097 | 1 | 3502786 | AT4G34660 |
| UQnapus1098 | 1 | 3549164 | AT4G34530 |
| UQnapus1106 | 1 | 4493357 | AT4G33740 |
| UQnapus1109 | 1 | 4708098 | AT4G33080 |
| UQnapus1122 | 1 | 6019824 |  |
| UQnapus1123 | 1 | 6089956 | AT4G29940 |
| UQnapus1128 | 1 | 6703711 | AT1G28020 |
| UQnapus1131 | 1 | 7097896 | AT4G17890 |
| UQnapus1137 | 1 | 7904025 | AT1G80070 |
| UQnapus1138 | 1 | 7940857 | AT4G19990 |
| UQnapus1145 | 1 | 8730254 | AT4G21895 |
| UQnapus1147 | 1 | 8866691 | AT4G22120 |
| UQnapus1151 | 1 | 9294398 | AT4G23190 |
| UQnapus1154 | 1 | 9680163 | AT4G23750 |
| UQnapus1155 | 1 | 9695191 | AT2G19170 |
| UQnapus1156 | 1 | 9746222 | AT4G23910 |
| UQnapus1158 | 1 | 10008608 | AT4G24380 |
| UQnapus1161 | 1 | 10276692 |  |
| UQnapus1166 | 1 | 10901850 | AT4G25720 |
| UQnapus1172 | 1 | 12192217 | AT4G28290 |
| UQnapus1177 | 1 | 13096092 | AT4G26600 |
| UQnapus1180 | 1 | 13238656 | AT4G26340 |
| UQnapus1186 | 1 | 14313207 | AT4G14790 |
| UQnapus1197 | 1 | 15645317 |  |
| UQnapus1198 | 1 | 15820384 | AT3G51440 |
| UQnapus1202 | 1 | 16733831 | AT3G48660 |
| UQnapus1205 | 1 | 16909575 | AT5G23740 |
| UQnapus1214 | 1 | 18465038 | AT1G21280 |
| UQnapus1222 | 1 | 19744909 | AT3G18400 |
| UQnapus1234 | 1 | 21551086 |  |
| UQnapus1243 | 1 | 24130772 | AT3G18030 |
| UQnapus1244 | 1 | 24427094 |  |
| UQnapus1245 | 1 | 24547404 | AT4G23160 |
| UQnapus1248 | 1 | 24876562 | AT3G16490 |
| UQnapus1250 | 1 | 24923148 |  |
| UQnapus1279 | 1 | 27570079 | AT3G12280 |
| UQnapus1298 | 1 | 29476782 | AT3G06070 |
| UQnapus1378 | 2 | 9049689 | AT5G55230 |
| UQnapus1388 | 2 | 9798312 |  |
| UQnapus1394 | 2 | 10334430 |  |
| UQnapus1395 | 2 | 10354797 | AT5G52390 |
| UQnapus1398 | 2 | 10578792 | AT5G51660 |
| UQnapus1399 | 2 | 10584203 | AT5G51660 |
| UQnapus1405 | 2 | 11221181 | AT1G70000 |
| UQnapus1424 | 2 | 13359293 | AT1G15460 |
| UQnapus1425 | 2 | 13428341 | AT1G75190 |
| UQnapus1427 | 2 | 13804505 | AT1G76010 |
| UQnapus1441 | 2 | 16652204 | AT1G67360 |
| UQnapus1442 | 2 | 16980226 | AT1G66970 |
| UQnapus1445 | 2 | 17249324 | AT1G68920 |
| UQnapus1450 | 2 | 17657373 | AT1G66070 |
| UQnapus1451 | 2 | 17926405 |  |
| UQnapus1470 | 2 | 21929017 |  |
| UQnapus1472 | 2 | 23179346 |  |
| UQnapus1487 | 2 | 25767207 | AT3G07010 |
| UQnapus1490 | 2 | 26211052 | AT5G27970 |
| UQnapus1533 | 3 | 3740504 | AT4G33200 |
| UQnapus1534 | 3 | 3807872 |  |
| UQnapus1536 | 3 | 3981770 |  |
| UQnapus1537 | 3 | 4148214 |  |
| UQnapus1540 | 3 | 4591468 | AT5G57900 |
| UQnapus1545 | 3 | 4987292 | AT4G30110 |
| UQnapus1546 | 3 | 5037821 |  |
| UQnapus1547 | 3 | 5164429 | AT5G39560 |
| UQnapus1548 | 3 | 5171899 | AT4G29790 |
| UQnapus1549 | 3 | 5238201 | AT4G29740 |
| UQnapus1553 | 3 | 5873396 | AT2G44210, AT4G27640 |
| UQnapus1554 | 3 | 5917408 | AT1G53100 |
| UQnapus1570 | 3 | 7154202 |  |
| UQnapus1642 | 3 | 16967056 | AT3G05690 |
| UQnapus1643 | 3 | 17017848 | AT3G05625 |
| UQnapus1644 | 3 | 17152509 | AT3G04910 |
| UQnapus1647 | 3 | 17600294 | AT3G02150 |
| UQnapus1652 | 3 | 18046738 | AT4G01010 |
| UQnapus1653 | 3 | 18072760 | AT4G00990 |
| UQnapus1664 | 3 | 18852080 | AT4G05410 |
| UQnapus1665 | 3 | 18908242 | AT4G04750 |
| UQnapus1667 | 3 | 18999222 | AT4G04460 |
| UQnapus1672 | 3 | 19644763 | AT4G09160 |
| UQnapus1676 | 3 | 20267830 | AT3G20770 |
| UQnapus1677 | 3 | 20321010 | AT2G27330 |
| UQnapus1680 | 3 | 20504072 |  |
| UQnapus1681 | 3 | 20512344 | AT5G54550 |
| UQnapus1693 | 3 | 21386277 | AT2G43820 |
| UQnapus1700 | 3 | 22101745 | AT2G36010 |
| UQnapus1702 | 3 | 22195259 | AT2G35795 |
| UQnapus1704 | 3 | 22278329 | AT4G38220 |
| UQnapus1705 | 3 | 22315523 | AT2G35190 |
| UQnapus1709 | 3 | 22748421 |  |
| UQnapus1710 | 3 | 22836153 | AT2G32750 |
| UQnapus1718 | 3 | 23497828 | AT2G30250 |
| UQnapus1722 | 3 | 23791031 | AT4G06634 |
| UQnapus1725 | 3 | 24132988 | AT5G52400 |
| UQnapus1726 | 3 | 24242461 | AT5G53635 |
| UQnapus1729 | 3 | 24456351 | AT5G53540 |
| UQnapus1730 | 3 | 24568308 | AT5G54170 |
| UQnapus1737 | 3 | 25134197 | AT3G50710 |
| UQnapus1738 | 3 | 25157083 |  |
| UQnapus1744 | 3 | 25601731 | AT5G58330 |
| UQnapus1789 | 3 | 29102871 | AT5G10510 |
| UQnapus1796 | 3 | 29936216 | AT3G26230 |
| UQnapus1819 | 4 | 1370620 | AT3G59820 |
| UQnapus1822 | 4 | 1651913 | AT3G58660 |
| UQnapus1827 | 4 | 1894331 | AT3G57700, AT2G41905 |
| UQnapus1828 | 4 | 1951210 | AT3G57610 |
| UQnapus1830 | 4 | 2147008 | AT3G57260 |
| UQnapus1831 | 4 | 2217306 | AT3G57070 |
| UQnapus1832 | 4 | 2265931 |  |
| UQnapus1833 | 4 | 2332844 | AT3G56810 |
| UQnapus1839 | 4 | 2937840 | AT2G37530 |
| UQnapus1843 | 4 | 3107814 | AT2G38040 |
| UQnapus1845 | 4 | 3330637 |  |
| UQnapus1846 | 4 | 3392923 | AT3G54620 |
| UQnapus1851 | 4 | 3759527 |  |
| UQnapus1853 | 4 | 4001630 | AT2G40815 |
| UQnapus1858 | 4 | 4521562 |  |
| UQnapus1860 | 4 | 4625353 | AT3G55610 |
| UQnapus1863 | 4 | 4823012 | AT3G55340 |
| UQnapus1866 | 4 | 5086383 | AT3G54840 |
| UQnapus1868 | 4 | 5408831 | AT3G54470 |
| UQnapus1869 | 4 | 5610471 | AT3G54140 |
| UQnapus1871 | 4 | 5714375 | AT3G53940 |
| UQnapus1876 | 4 | 6551420 | AT3G52200 |
| UQnapus1878 | 4 | 6823156 |  |
| UQnapus1884 | 4 | 8056604 |  |
| UQnapus1888 | 4 | 8714797 | AT2G05260 |
| UQnapus1903 | 4 | 11638847 | AT5G41610 |
| UQnapus1933 | 4 | 15938061 | AT1G61790 |
| UQnapus1938 | 4 | 16566695 |  |
| UQnapus1939 | 4 | 16689941 | AT1G60980 |
| UQnapus1940 | 4 | 16790832 | AT1G05530 |
| UQnapus1946 | 4 | 17342334 | AT4G20280 |
| UQnapus1947 | 4 | 17377025 | AT1G59910 |
| UQnapus1948 | 4 | 17384979 |  |
| UQnapus1949 | 4 | 17435363 | AT1G59820 |
| UQnapus1952 | 4 | 17613556 | AT1G59520 |
| UQnapus1956 | 4 | 18138153 |  |
| UQnapus1958 | 4 | 18379191 | AT3G47090 |
| UQnapus1959 | 4 | 18450870 | AT2G28040, AT2G28440 |
| UQnapus1960 | 4 | 18483351 | AT2G28360 |
| UQnapus1966 | 4 | 18839247 | AT5G03495 |
| UQnapus1970 | 4 | 19194405 | AT1G06200 |
| UQnapus1972 | 4 | 19616259 | AT5G65850 |
| UQnapus1984 | 4 | 20785543 | AT2G29580 |
| UQnapus2134 | 5 | 11503954 | AT4G02460 |
| UQnapus2241 | 6 | 3665238 |  |
| UQnapus2342 | 6 | 20912986 | AT3G28470 |
| UQnapus2346 | 6 | 21752978 | AT3G26130 |
| UQnapus2347 | 6 | 22252121 | AT2G02570 |
| UQnapus2348 | 6 | 22322501 | AT2G02230 |
| UQnapus2479 | 7 | 12029455 | AT4G09580 |
| UQnapus2480 | 7 | 12364106 |  |
| UQnapus2719 | 8 | 14518407 | AT5G51010 |
| UQnapus2769 | 8 | 19732881 | AT3G26050 |
| UQnapus2839 | 9 | 9512902 | AT4G16750, AT2G35700 |
| UQnapus2850 | 9 | 10782486 | AT3G51310 |
| UQnapus2851 | 9 | 10846636 | AT3G51150 |
| UQnapus2855 | 9 | 11541918 |  |
| UQnapus2856 | 9 | 11753932 |  |
| UQnapus2858 | 9 | 11847423 | AT5G02500 |
| UQnapus2859 | 9 | 12122883 | AT1G27045 |
| UQnapus2861 | 9 | 12324185 | AT5G53230 |
| UQnapus2862 | 9 | 12329652 | AT1G24000 |
| UQnapus2865 | 9 | 12906074 | AT1G23480 |
| UQnapus2869 | 9 | 13853333 |  |
| UQnapus2870 | 9 | 14014370 | AT1G30260 |
| UQnapus2873 | 9 | 16052823 |  |
| UQnapus2874 | 9 | 16282836 | AT1G32950 |
| UQnapus2876 | 9 | 16545089 | AT1G35340 |
| UQnapus2877 | 9 | 16851256 |  |
| UQnapus2880 | 9 | 16945793 |  |
| UQnapus2881 | 9 | 16984519 |  |
| UQnapus2883 | 9 | 17345939 |  |
| UQnapus2884 | 9 | 17521704 | AT1G33770 |
| UQnapus2906 | 9 | 23394996 |  |
| UQnapus2909 | 9 | 23863948 | AT3G56520 |
| UQnapus2970 | 1unassigned A genome | 527285 | AT5G04140 |
| UQnapus2973 | 1unassigned A genome | 946852 |  |
| UQnapus2977 | 1unassigned A genome | 1096474 | AT5G08560 |
| UQnapus2980 | 1unassigned A genome | 1321785 | AT5G07670 |
| UQnapus2983 | 1unassigned A genome | 1713261 | AT5G05980 |
| UQnapus2985 | 1unassigned A genome | 1913326 | AT5G05360 |
| UQnapus2987 | 1unassigned A genome | 2011057 | AT5G09850 |
| UQnapus2989 | 1unassigned A genome | 2149251 | AT5G10200 |
| UQnapus2993 | 1unassigned A genome | 2340831 | AT4G00670 |
| UQnapus3003 | 1unassigned A genome | 2795983 | AT3G11010, AT5G11880 |
| UQnapus3004 | 1unassigned A genome | 2804360 | AT5G11900 |
| UQnapus3007 | 1unassigned A genome | 2935126 |  |
| UQnapus3149 | 11 | 506300 |  |
| UQnapus3186 | 11 | 4566544 |  |
| UQnapus3241 | 11 | 7251507 | AT4G27420 |
| UQnapus3243 | 11 | 7772507 | AT4G27680 |
| UQnapus3310 | 11 | 11964959 | AT3G48810 |
| UQnapus3312 | 11 | 12019769 |  |
| UQnapus3316 | 11 | 12060395 | AT3G48740 |
| UQnapus3319 | 11 | 12127409 |  |
| UQnapus3320 | 11 | 12143414 |  |
| UQnapus3324 | 11 | 12212495 | AT3G48610 |
| UQnapus3325 | 11 | 12221499 |  |
| UQnapus3327 | 11 | 12255449 |  |
| UQnapus3328 | 11 | 12275741 |  |
| UQnapus3335 | 11 | 12479678 |  |
| UQnapus3342 | 11 | 13626543 |  |
| UQnapus3407 | 12 | 1511298 |  |
| UQnapus3513 | 12 | 8621063 | AT5G52140 |
| UQnapus3572 | 12 | 13780262 | AT1G76680 |
| UQnapus3669 | 13 | 6303582 | AT4G04650 |
| UQnapus3732 | 13 | 9940428 | AT5G19290 |
| UQnapus3760 | 13 | 12832924 |  |
| UQnapus3764 | 13 | 13371673 |  |
| UQnapus3765 | 13 | 13383794 |  |
| UQnapus3767 | 13 | 13521802 | AT2G20625 |
| UQnapus3833 | 13 | 19930807 |  |
| UQnapus3837 | 13 | 20131440 | AT4G37040 |
| UQnapus3842 | 13 | 20743627 |  |
| UQnapus3853 | 13 | 21475108 | AT1G48950 |
| UQnapus3875 | 13 | 24675538 | AT5G53350 |
| UQnapus3876 | 13 | 24700477 | AT3G02010 |
| UQnapus3877 | 13 | 24841058 | AT5G40700 |
| UQnapus3878 | 13 | 24880690 | AT1G40390 |
| UQnapus4000 | 14 | 8575749 |  |
| UQnapus4009 | 14 | 10328142 |  |
| UQnapus4011 | 14 | 10609935 | AT2G34140 |
| UQnapus4012 | 14 | 10690541 | AT2G34440 |
| UQnapus4014 | 14 | 10777975 | AT2G34610 |
| UQnapus4016 | 14 | 10877895 |  |
| UQnapus4018 | 14 | 11037989 | AT2G35250 |
| UQnapus4021 | 14 | 11327312 |  |
| UQnapus4032 | 14 | 12532385 | AT2G38070 |
| UQnapus4033 | 14 | 12567523 | AT2G38110 |
| UQnapus4039 | 14 | 12827812 |  |
| UQnapus4042 | 14 | 12944225 | AT4G24250 |
| UQnapus4044 | 14 | 13011587 | AT2G39340 |
| UQnapus4058 | 15 | 1418021 |  |
| UQnapus4059 | 15 | 1434237 |  |
| UQnapus4065 | 15 | 2247663 |  |
| UQnapus4075 | 15 | 3227039 | AT1G10417 |
| UQnapus4096 | 15 | 5288875 | AT1G05805 |
| UQnapus4099 | 15 | 6043418 | AT1G16130 |
| UQnapus4100 | 15 | 6137099 | AT1G15825 |
| UQnapus4105 | 15 | 7721304 | AT1G69160 |
| UQnapus4108 | 15 | 7914039 | AT1G69500 |
| UQnapus4115 | 15 | 8766609 | AT1G70430 |
| UQnapus4153 | 16 | 2276051 | AT3G59920 |
| UQnapus4218 | 17 | 4820620 |  |
| UQnapus4219 | 17 | 4846357 |  |
| UQnapus4221 | 17 | 4983859 |  |
| UQnapus4278 | 17 | 9785789 |  |
| UQnapus4366 | 17 | 17474299 | AT3G24255 |
| UQnapus4367 | 17 | 17479612 |  |
| UQnapus4371 | 17 | 17550106 | AT4G18710 |
| UQnapus4372 | 17 | 17569470 |  |
| UQnapus4373 | 17 | 17595362 | AT1G43760 |
| UQnapus4375 | 17 | 17728521 | AT3G14420 |
| UQnapus4377 | 17 | 17787581 |  |
| UQnapus4379 | 17 | 17820559 |  |
| UQnapus4380 | 17 | 17828554 | AT5G46370, AT4G18160 |
| UQnapus4390 | 17 | 18411249 | AT4G24540 |
| UQnapus4397 | 17 | 18672302 | AT4G23790 |
| UQnapus4401 | 18 | 312952 |  |
| UQnapus4502 | 18 | 8622160 | AT2G06845 |
| UQnapus4537 | 18 | 11301021 |  |
| UQnapus4606 | 19 | 6863450 |  |
| UQnapus4610 | 19 | 7452033 | AT5G60660 |
| UQnapus4622 | 19 | 8866492 |  |
| UQnapus4623 | 19 | 9006084 | AT5G17410 |
| UQnapus4624 | 19 | 9006101 | AT5G17410 |
| UQnapus4633 | 1 | 9599506 |  |
| UQnapus4650 | 5 | 6214158 | AT2G01820 |
| UQnapus4674 | 9 | 17434381 | AT3G55080 |
| UQnapus4686 | 1 | 26010734 |  |
| UQnapus4687 | 3 | 20995020 |  |
| UQnapus4711 | 13 | 13319267 |  |
| UQnapus4718 | 14 | 9049140 |  |
| UQnapus4804 | 1 | 7194790 | AT4G18130 |
| UQnapus4807 | 15 | 985209 | AT1G04540 |
| UQnapus4810 | unassigned C genome | 30711285 | AT4G09490 |
| UQnapus4816 | unassigned A genome | 23860537 | AT4G34220 |
| UQnapus4817 | 13 | 24772351 |  |
| UQnapus4818 | 17 | 14438269 | AT4G15393 |
| UQnapus4825 | 1 | 6511156 |  |
| UQnapus4827 | 1 | 9061023 |  |
| UQnapus4845 | 4 | 2445176 |  |
| UQnapus4885 | 12 | 14207820 |  |
| UQnapus4890 | 13 | 19782357 | AT4G36380 |
| UQnapus4901 | 15 | 7693708 | AT1G69080 |
| UQnapus4934 | unassigned C genome | 283157102 |  |
| UQnapus4987 | 3 | 21986397 |  |
| UQnapus5033 | 1 | 6568009 | AT4G29010, AT4G29000 |
| UQnapus5037 | 3 | 17600294 | AT3G02150 |
| UQnapus5038 | 3 | 17795636 |  |
| UQnapus5051 | 8 | 12083647 |  |
| UQnapus5055 | 9 | 9512902 | AT4G16750, AT2G35700 |
| UQnapus5067 | 18 | 8622160 | AT2G06845 |
| UQnapus5081 | 3 | 27900030 |  |
| UQnapus5084 | 4 | 3235219 | AT2G38370 |
| UQnapus5086 | 4 | 9568415 |  |
| UQnapus5111 | 1 | 15820384 | AT3G51440 |
| UQnapus5166 | 2 | 17456430 | AT1G68660 |
| UQnapus5172 | 6 | 22522149 | AT2G01820 |
| UQnapus5183 | 1 | 7952655 | AT4G20000 |
| UQnapus5184 | 1 | 24431616 |  |
| UQnapus5196 | 4 | 4917155 |  |
| UQnapus5268 | 1 | 9294398 | AT4G23190 |
| UQnapus5269 | 1 | 9760306 | AT4G23950 |
| UQnapus5278 | 3 | 5223360 |  |
| UQnapus5281 | 3 | 22195259 | AT2G35795 |
| UQnapus5307 | 9 | 11714623 | AT5G16990 |
| UQnapus5331 | 1 | 16849128 | AT3G48810 |
| UQnapus5337 | 4 | 4193693 | AT2G41630 |
| UQnapus5342 | 6 | 3716229 |  |
| UQnapus5363 | 1 | 1434461 | AT4G35800 |
| UQnapus5365 | 1 | 9050498 | AT4G22540 |
| UQnapus5374 | 3 | 22579671 | AT2G33830 |
| UQnapus5379 | 4 | 5736202 | AT3G53830 |
| UQnapus5381 | 4 | 16904460 |  |
| UQnapus5383 | 4 | 18674116 | AT2G30230 |
| UQnapus5384 | 4 | 18839247 | AT5G03495 |
| UQnapus5385 | 4 | 20755535 |  |
| UQnapus5437 | 11 | 12342816 |  |
| UQnapus5441 | 2 | 5541126 | AT5G17850 |
| UQnapus5448 | 3 | 23889207 | AT5G51545 |
| UQnapus5469 | 9 | 18919574 | AT4G05140 |
| UQnapus5471 | 1unassigned A genome | 1859000 | AT5G05670 |
| UQnapus5486 | 1 | 200737 | AT5G09870, AT5G64740, AT2G21770, AT4G39350 |
| UQnapus5487 | 1 | 451622 | AT4G40030 |
| UQnapus5488 | 1 | 964842 | AT4G37080 |
| UQnapus5494 | 1 | 5448592 | AT4G31540 |
| UQnapus5495 | 1 | 6708268 | AT4G28710 |
| UQnapus5497 | 1 | 7492027 | AT4G18600 |
| UQnapus5500 | 1 | 11641609 |  |
| UQnapus5510 | 1 | 21539695 | AT1G43760 |
| UQnapus5541 | 2 | 14161545 | AT1G76900 |
| UQnapus5543 | 2 | 15399836 | AT2G26000 |
| UQnapus5552 | 2 | 23179346 |  |
| UQnapus5554 | 2 | 24033303 |  |
| UQnapus5556 | 2 | 26143787 |  |
| UQnapus5561 | 3 | 4591468 | AT5G57900 |
| UQnapus5575 | 3 | 20562081 |  |
| UQnapus5578 | 3 | 21768043 | AT4G39550 |
| UQnapus5584 | 3 | 29135636 | AT5G65430 |
| UQnapus5588 | 4 | 2225189 | AT3G57060 |
| UQnapus5590 | 4 | 3774042 | AT2G40030 |
| UQnapus5593 | 4 | 4705719 |  |
| UQnapus5604 | 4 | 16715646 | AT1G60900 |
| UQnapus5627 | 5 | 8960210 |  |
| UQnapus5656 | 6 | 6452814 | AT3G13410 |
| UQnapus5708 | 8 | 14518407 | AT5G51010 |
| UQnapus5739 | 9 | 24439446 | AT3G22950 |
| UQnapus5749 | 1unassigned A genome | 946852 |  |
| UQnapus5750 | 1unassigned A genome | 1096474 | AT5G08560 |
| UQnapus5751 | 1unassigned A genome | 1321785 | AT5G07670 |
| UQnapus5758 | 1unassigned A genome | 2795983 | AT3G11010, AT5G11880 |
| UQnapus5819 | 11 | 1802110 |  |
| UQnapus5841 | 11 | 12236791 |  |
| UQnapus5852 | 2 | 12204997 | AT1G72230 |
| UQnapus5861 | 13 | 9915591 | AT5G19260 |
| UQnapus5879 | 11 | 13638645 |  |
| UQnapus5902 | 1 | 394626 | AT4G39840 |
| UQnapus5903 | 3 | 18342052 |  |
| UQnapus5913 | 4 | 2409021 |  |
| UQnapus5914 | 4 | 3176283 |  |
| UQnapus5983 | unassigned C genome | 33478479 | AT4G18350 |
| UQnapus5988 | unassigned C genome | 313798054 |  |
| UQnapus5991 | unassigned C genome | 348705209 | AT4G33400 |
