# Supplementary material for: Genome Wide Analysis of Flowering Time Trait in Multiple Environments via High-Throughput Genotyping Technique in Brassica napus L
Source: PLoS One. 2015 Mar 19;10(3):e0119425. doi: 10.1371/journal.pone.0119425 (PMC4366152; doi:10.1371/journal.pone.0119425)
Supplement: S2 Table — (DOCX) [file pone.0119425.s004.docx]

**S2 Table**. The associated tag SNPs and their representing SNPs in each of the geographic sites.

| Geographic sites | Score | SNP | Represented SNP | R2 |
| --- | --- | --- | --- | --- |
| North | 20.24 | UQnapus0669 | UQnapus0669 | 1.00 |
|  | 20.24 | UQnapus0669 | UQnapus0816 | 0.91 |
|  | 20.24 | UQnapus0669 | UQnapus4901 | 0.89 |
|  | 20.24 | UQnapus0669 | UQnapus0104 | 0.88 |
|  | 20.24 | UQnapus0669 | UQnapus4105 | 0.89 |
|  | 20.24 | UQnapus0669 | UQnapus4108 | 0.89 |
|  | 20.24 | UQnapus0669 | UQnapus4115 | 1.00 |
|  | 20.24 | UQnapus0669 | UQnapus4278 | 0.80 |
|  | 20.24 | UQnapus0669 | UQnapus0052 | 0.89 |
|  | 13.31 | UQnapus4622 | UQnapus4622 | 1.00 |
|  | 13.31 | UQnapus4622 | UQnapus4623 | 0.98 |
|  | 13.31 | UQnapus4622 | UQnapus4624 | 0.98 |
|  | 13.31 | UQnapus4622 | UQnapus5081 | 0.98 |
|  | 13.31 | UQnapus4622 | UQnapus5861 | 0.72 |
|  | 13.31 | UQnapus4622 | UQnapus3732 | 0.72 |
|  | 12.93 | UQnapus4153 | UQnapus4153 | 1.00 |
|  | 12.30 | UQnapus0907 | UQnapus0907 | 1.00 |
|  | 12.30 | UQnapus0907 | UQnapus4278 | 0.71 |
|  | 10.04 | UQnapus5033 | UQnapus5033 | 1.00 |
|  | 10.04 | UQnapus5033 | UQnapus1128 | 0.80 |
|  | 10.04 | UQnapus5033 | UQnapus5495 | 0.80 |
|  | 10.04 | UQnapus5033 | UQnapus1131 | 0.86 |
|  | 10.04 | UQnapus5033 | UQnapus4804 | 0.78 |
|  | 10.04 | UQnapus5033 | UQnapus5497 | 0.78 |
|  | 10.04 | UQnapus5033 | UQnapus1145 | 0.72 |
|  | 10.04 | UQnapus5033 | UQnapus5983 | 0.80 |
|  | 10.04 | UQnapus5033 | UQnapus1122 | 0.82 |
|  | 10.04 | UQnapus5033 | UQnapus1123 | 0.83 |
|  | 10.04 | UQnapus5033 | UQnapus4825 | 0.79 |
|  | 9.80 | UQnapus4219 | UQnapus4219 | 1.00 |
|  | 9.80 | UQnapus4219 | UQnapus4221 | 1.00 |
|  | 9.80 | UQnapus4219 | UQnapus4401 | 0.73 |
|  | 9.80 | UQnapus4219 | UQnapus4711 | 0.83 |
|  | 9.80 | UQnapus4219 | UQnapus3764 | 0.84 |
|  | 9.80 | UQnapus4219 | UQnapus3765 | 0.84 |
|  | 9.80 | UQnapus4219 | UQnapus3767 | 0.84 |
|  | 9.80 | UQnapus4219 | UQnapus0144 | 0.85 |
|  | 9.80 | UQnapus4219 | UQnapus4218 | 1.00 |
|  | 9.40 | UQnapus1545 | UQnapus1545 | 1.00 |
|  | 9.40 | UQnapus1545 | UQnapus1546 | 0.95 |
|  | 9.40 | UQnapus1545 | UQnapus1547 | 0.88 |
|  | 9.40 | UQnapus1545 | UQnapus1548 | 0.84 |
|  | 9.40 | UQnapus1545 | UQnapus5278 | 0.91 |
|  | 9.40 | UQnapus1545 | UQnapus1549 | 0.91 |
|  | 9.40 | UQnapus1545 | UQnapus1553 | 0.86 |
|  | 9.40 | UQnapus1545 | UQnapus1554 | 0.72 |
|  | 9.40 | UQnapus1545 | UQnapus4650 | 0.76 |
|  | 9.40 | UQnapus1545 | UQnapus1533 | 0.72 |
|  | 9.40 | UQnapus1545 | UQnapus1534 | 0.78 |
|  | 9.40 | UQnapus1545 | UQnapus1536 | 0.76 |
|  | 9.40 | UQnapus1545 | UQnapus1537 | 0.77 |
|  | 9.40 | UQnapus1545 | UQnapus1540 | 0.89 |
|  | 9.40 | UQnapus1545 | UQnapus5561 | 0.89 |
|  | 8.81 | UQnapus0863 | UQnapus0863 | 1.00 |
|  | 8.81 | UQnapus0863 | UQnapus0712 | 0.93 |
|  | 8.51 | UQnapus0394 | UQnapus0394 | 1.00 |
|  | 8.34 | UQnapus1298 | UQnapus1298 | 1.00 |
|  | 8.31 | UQnapus1022 | UQnapus1022 | 1.00 |
|  | 8.31 | UQnapus1022 | UQnapus5494 | 0.86 |
|  | 7.60 | UQnapus3669 | UQnapus3669 | 1.00 |
|  | 7.47 | UQnapus0743 | UQnapus0743 | 1.00 |
|  | 7.47 | UQnapus0743 | UQnapus0054 | 0.83 |
|  | 7.30 | UQnapus1106 | UQnapus1106 | 1.00 |
|  | 7.30 | UQnapus1106 | UQnapus1109 | 0.83 |
|  | 7.30 | UQnapus1106 | UQnapus4816 | 0.87 |
|  | 7.30 | UQnapus1106 | UQnapus1070 | 0.76 |
|  | 7.30 | UQnapus1106 | UQnapus1073 | 0.83 |
|  | 7.30 | UQnapus1106 | UQnapus5363 | 0.70 |
|  | 7.30 | UQnapus1106 | UQnapus1097 | 0.84 |
|  | 7.30 | UQnapus1106 | UQnapus1098 | 0.82 |
|  | 7.15 | UQnapus1789 | UQnapus1789 | 1.00 |
|  | 7.15 | UQnapus1789 | UQnapus5584 | 0.95 |
|  | 7.15 | UQnapus1789 | UQnapus1796 | 0.76 |
|  | 7.15 | UQnapus1789 | UQnapus0203 | 0.73 |
|  | 6.86 | UQnapus1166 | UQnapus1166 | 1.00 |
|  | 6.86 | UQnapus1166 | UQnapus5500 | 0.77 |
|  | 6.86 | UQnapus1166 | UQnapus1172 | 0.83 |
|  | 6.86 | UQnapus1166 | UQnapus1177 | 0.95 |
|  | 6.86 | UQnapus1166 | UQnapus1180 | 0.98 |
|  | 6.86 | UQnapus1166 | UQnapus1186 | 0.73 |
|  | 6.86 | UQnapus1166 | UQnapus1197 | 0.73 |
|  | 6.86 | UQnapus1166 | UQnapus1198 | 0.73 |
|  | 6.86 | UQnapus1166 | UQnapus5111 | 0.73 |
|  | 6.86 | UQnapus1166 | UQnapus1202 | 0.71 |
|  | 6.86 | UQnapus1166 | UQnapus5331 | 0.73 |
|  | 6.86 | UQnapus1166 | UQnapus1205 | 0.73 |
|  | 6.86 | UQnapus1166 | UQnapus5510 | 0.75 |
|  | 6.86 | UQnapus1166 | UQnapus1234 | 0.77 |
|  | 6.86 | UQnapus1166 | UQnapus1570 | 0.73 |
|  | 6.86 | UQnapus1166 | UQnapus1933 | 0.77 |
|  | 6.86 | UQnapus1166 | UQnapus1938 | 0.75 |
|  | 6.86 | UQnapus1166 | UQnapus1939 | 0.77 |
|  | 6.86 | UQnapus1166 | UQnapus5604 | 0.73 |
|  | 6.86 | UQnapus1166 | UQnapus1940 | 0.74 |
|  | 6.86 | UQnapus1166 | UQnapus5381 | 0.73 |
|  | 6.86 | UQnapus1166 | UQnapus1946 | 0.73 |
|  | 6.86 | UQnapus1166 | UQnapus1947 | 0.73 |
|  | 6.86 | UQnapus1166 | UQnapus1948 | 0.73 |
|  | 6.86 | UQnapus1166 | UQnapus1949 | 0.73 |
|  | 6.86 | UQnapus1166 | UQnapus1952 | 0.73 |
|  | 6.86 | UQnapus1166 | UQnapus5739 | 0.73 |
|  | 6.86 | UQnapus1166 | UQnapus5497 | 0.73 |
|  | 6.86 | UQnapus1166 | UQnapus1137 | 0.75 |
|  | 6.86 | UQnapus1166 | UQnapus1138 | 0.80 |
|  | 6.86 | UQnapus1166 | UQnapus5183 | 0.80 |
|  | 6.86 | UQnapus1166 | UQnapus1145 | 0.79 |
|  | 6.86 | UQnapus1166 | UQnapus1147 | 0.85 |
|  | 6.86 | UQnapus1166 | UQnapus5365 | 0.85 |
|  | 6.86 | UQnapus1166 | UQnapus4827 | 0.87 |
|  | 6.86 | UQnapus1166 | UQnapus1151 | 0.87 |
|  | 6.86 | UQnapus1166 | UQnapus5268 | 0.87 |
|  | 6.86 | UQnapus1166 | UQnapus4633 | 0.86 |
|  | 6.86 | UQnapus1166 | UQnapus1154 | 0.83 |
|  | 6.86 | UQnapus1166 | UQnapus1155 | 0.83 |
|  | 6.86 | UQnapus1166 | UQnapus1156 | 0.83 |
|  | 6.86 | UQnapus1166 | UQnapus5269 | 0.87 |
|  | 6.86 | UQnapus1166 | UQnapus1158 | 0.87 |
|  | 6.86 | UQnapus1166 | UQnapus1161 | 0.83 |
|  | 6.77 | UQnapus5471 | UQnapus5471 | 1.00 |
|  | 6.77 | UQnapus5471 | UQnapus2985 | 0.87 |
|  | 6.77 | UQnapus5471 | UQnapus0874 | 0.83 |
|  | 6.77 | UQnapus5471 | UQnapus0224 | 0.72 |
|  | 6.77 | UQnapus5471 | UQnapus2970 | 0.72 |
|  | 6.77 | UQnapus5471 | UQnapus2980 | 0.83 |
|  | 6.77 | UQnapus5471 | UQnapus5751 | 0.83 |
|  | 6.77 | UQnapus5471 | UQnapus2983 | 0.91 |
|  | 6.37 | UQnapus4075 | UQnapus4075 | 1.00 |
|  | 6.37 | UQnapus4075 | UQnapus0406 | 0.91 |
|  | 6.37 | UQnapus4075 | UQnapus4065 | 0.85 |
|  | 6.22 | UQnapus0689 | UQnapus0689 | 1.00 |
|  | 6.22 | UQnapus0689 | UQnapus0927 | 0.79 |
|  | 6.22 | UQnapus0689 | UQnapus1046 | 0.74 |
|  | 6.22 | UQnapus0689 | UQnapus4000 | 0.75 |
|  | 6.22 | UQnapus0689 | UQnapus4718 | 0.76 |
|  | 6.22 | UQnapus0689 | UQnapus4009 | 0.89 |
|  | 6.22 | UQnapus0689 | UQnapus4011 | 0.87 |
|  | 6.22 | UQnapus0689 | UQnapus4012 | 0.89 |
|  | 6.22 | UQnapus0689 | UQnapus4014 | 0.85 |
|  | 6.22 | UQnapus0689 | UQnapus4016 | 0.85 |
|  | 6.22 | UQnapus0689 | UQnapus4018 | 0.87 |
|  | 6.22 | UQnapus0689 | UQnapus4021 | 0.85 |
|  | 6.22 | UQnapus0689 | UQnapus4058 | 0.96 |
|  | 6.22 | UQnapus0689 | UQnapus4059 | 0.96 |
|  | 5.78 | UQnapus1450 | UQnapus1450 | 1.00 |
|  | 5.78 | UQnapus1450 | UQnapus1451 | 0.95 |
|  | 5.78 | UQnapus1450 | UQnapus5578 | 0.80 |
|  | 5.78 | UQnapus1450 | UQnapus4987 | 1.00 |
|  | 5.78 | UQnapus1450 | UQnapus3513 | 0.76 |
|  | 5.78 | UQnapus1450 | UQnapus0293 | 0.81 |
|  | 5.78 | UQnapus1450 | UQnapus1378 | 0.73 |
|  | 5.78 | UQnapus1450 | UQnapus1388 | 0.72 |
|  | 5.78 | UQnapus1450 | UQnapus1394 | 0.75 |
|  | 5.78 | UQnapus1450 | UQnapus1395 | 0.74 |
|  | 5.78 | UQnapus1450 | UQnapus0097 | 0.74 |
|  | 5.78 | UQnapus1450 | UQnapus0125 | 0.76 |
|  | 5.78 | UQnapus1450 | UQnapus1398 | 0.76 |
|  | 5.78 | UQnapus1450 | UQnapus1399 | 0.74 |
|  | 5.78 | UQnapus1450 | UQnapus1405 | 0.75 |
|  | 5.78 | UQnapus1450 | UQnapus5852 | 0.78 |
|  | 5.78 | UQnapus1450 | UQnapus1424 | 0.72 |
|  | 5.78 | UQnapus1450 | UQnapus1427 | 0.70 |
|  | 5.78 | UQnapus1450 | UQnapus1441 | 0.84 |
|  | 5.78 | UQnapus1450 | UQnapus1442 | 0.86 |
|  | 5.78 | UQnapus1450 | UQnapus1445 | 0.89 |
|  | 5.78 | UQnapus1450 | UQnapus5166 | 0.82 |
|  | 5.40 | UQnapus4096 | UQnapus4096 | 1.00 |
|  | 5.40 | UQnapus4096 | UQnapus4807 | 0.83 |
|  | 5.39 | UQnapus5988 | UQnapus5988 | 1.00 |
|  | 5.30 | UQnapus3878 | UQnapus3878 | 1.00 |
|  | 5.30 | UQnapus3878 | UQnapus0594 | 0.87 |
|  | 5.30 | UQnapus3878 | UQnapus0604 | 0.85 |
|  | 5.30 | UQnapus3878 | UQnapus0806 | 0.87 |
|  | 5.30 | UQnapus3878 | UQnapus0896 | 0.87 |
|  | 5.30 | UQnapus3878 | UQnapus0911 | 0.85 |
|  | 5.30 | UQnapus3878 | UQnapus0974 | 0.97 |
|  | 5.30 | UQnapus3878 | UQnapus0351 | 1.00 |
|  | 5.30 | UQnapus3878 | UQnapus3149 | 0.87 |
|  | 5.30 | UQnapus3878 | UQnapus3875 | 1.00 |
|  | 5.30 | UQnapus3878 | UQnapus3876 | 1.00 |
|  | 5.30 | UQnapus3878 | UQnapus4817 | 1.00 |
|  | 5.30 | UQnapus3878 | UQnapus3877 | 1.00 |
|  | 5.21 | UQnapus1026 | UQnapus1026 | 1.00 |
|  | 5.21 | UQnapus1026 | UQnapus4606 | 0.98 |
|  | 5.21 | UQnapus1026 | UQnapus4610 | 0.73 |
|  | 5.21 | UQnapus1026 | UQnapus0439 | 1.00 |
|  | 5.21 | UQnapus1026 | UQnapus0535 | 0.98 |
|  | 5.21 | UQnapus1026 | UQnapus0976 | 0.85 |
| South | 23.74 | UQnapus0669 | UQnapus0669 | 1.00 |
|  | 23.74 | UQnapus0669 | UQnapus0816 | 0.91 |
|  | 23.74 | UQnapus0669 | UQnapus4901 | 0.89 |
|  | 23.74 | UQnapus0669 | UQnapus0104 | 0.88 |
|  | 23.74 | UQnapus0669 | UQnapus4105 | 0.89 |
|  | 23.74 | UQnapus0669 | UQnapus4108 | 0.89 |
|  | 23.74 | UQnapus0669 | UQnapus4115 | 1.00 |
|  | 23.74 | UQnapus0669 | UQnapus4278 | 0.80 |
|  | 23.74 | UQnapus0669 | UQnapus0052 | 0.89 |
|  | 12.78 | UQnapus4153 | UQnapus4153 | 1.00 |
|  | 11.00 | UQnapus1450 | UQnapus1450 | 1.00 |
|  | 11.00 | UQnapus1450 | UQnapus1451 | 0.95 |
|  | 11.00 | UQnapus1450 | UQnapus5578 | 0.80 |
|  | 11.00 | UQnapus1450 | UQnapus4987 | 1.00 |
|  | 11.00 | UQnapus1450 | UQnapus3513 | 0.76 |
|  | 11.00 | UQnapus1450 | UQnapus0293 | 0.81 |
|  | 11.00 | UQnapus1450 | UQnapus1378 | 0.73 |
|  | 11.00 | UQnapus1450 | UQnapus1388 | 0.72 |
|  | 11.00 | UQnapus1450 | UQnapus1394 | 0.75 |
|  | 11.00 | UQnapus1450 | UQnapus1395 | 0.74 |
|  | 11.00 | UQnapus1450 | UQnapus0097 | 0.74 |
|  | 11.00 | UQnapus1450 | UQnapus0125 | 0.76 |
|  | 11.00 | UQnapus1450 | UQnapus1398 | 0.76 |
|  | 11.00 | UQnapus1450 | UQnapus1399 | 0.74 |
|  | 11.00 | UQnapus1450 | UQnapus1405 | 0.75 |
|  | 11.00 | UQnapus1450 | UQnapus5852 | 0.78 |
|  | 11.00 | UQnapus1450 | UQnapus1424 | 0.72 |
|  | 11.00 | UQnapus1450 | UQnapus1427 | 0.70 |
|  | 11.00 | UQnapus1450 | UQnapus1441 | 0.84 |
|  | 11.00 | UQnapus1450 | UQnapus1442 | 0.86 |
|  | 11.00 | UQnapus1450 | UQnapus1445 | 0.89 |
|  | 11.00 | UQnapus1450 | UQnapus5166 | 0.82 |
|  | 10.96 | UQnapus0394 | UQnapus0394 | 1.00 |
|  | 10.83 | UQnapus1106 | UQnapus1106 | 1.00 |
|  | 10.83 | UQnapus1106 | UQnapus1109 | 0.83 |
|  | 10.83 | UQnapus1106 | UQnapus4816 | 0.87 |
|  | 10.83 | UQnapus1106 | UQnapus1070 | 0.76 |
|  | 10.83 | UQnapus1106 | UQnapus1073 | 0.83 |
|  | 10.83 | UQnapus1106 | UQnapus5363 | 0.70 |
|  | 10.83 | UQnapus1106 | UQnapus1097 | 0.84 |
|  | 10.83 | UQnapus1106 | UQnapus1098 | 0.82 |
|  | 10.76 | UQnapus5033 | UQnapus5033 | 1.00 |
|  | 10.76 | UQnapus5033 | UQnapus1128 | 0.80 |
|  | 10.76 | UQnapus5033 | UQnapus5495 | 0.80 |
|  | 10.76 | UQnapus5033 | UQnapus1131 | 0.86 |
|  | 10.76 | UQnapus5033 | UQnapus4804 | 0.78 |
|  | 10.76 | UQnapus5033 | UQnapus5497 | 0.78 |
|  | 10.76 | UQnapus5033 | UQnapus1145 | 0.72 |
|  | 10.76 | UQnapus5033 | UQnapus5983 | 0.80 |
|  | 10.76 | UQnapus5033 | UQnapus1122 | 0.82 |
|  | 10.76 | UQnapus5033 | UQnapus1123 | 0.83 |
|  | 10.76 | UQnapus5033 | UQnapus4825 | 0.79 |
|  | 10.33 | UQnapus4219 | UQnapus4219 | 1.00 |
|  | 10.33 | UQnapus4219 | UQnapus4221 | 1.00 |
|  | 10.33 | UQnapus4219 | UQnapus4401 | 0.73 |
|  | 10.33 | UQnapus4219 | UQnapus4711 | 0.83 |
|  | 10.33 | UQnapus4219 | UQnapus3764 | 0.84 |
|  | 10.33 | UQnapus4219 | UQnapus3765 | 0.84 |
|  | 10.33 | UQnapus4219 | UQnapus3767 | 0.84 |
|  | 10.33 | UQnapus4219 | UQnapus0144 | 0.85 |
|  | 10.33 | UQnapus4219 | UQnapus4218 | 1.00 |
|  | 9.98 | UQnapus0863 | UQnapus0863 | 1.00 |
|  | 9.98 | UQnapus0863 | UQnapus0712 | 0.93 |
|  | 9.72 | UQnapus1166 | UQnapus1166 | 1.00 |
|  | 9.72 | UQnapus1166 | UQnapus5500 | 0.77 |
|  | 9.72 | UQnapus1166 | UQnapus1172 | 0.83 |
|  | 9.72 | UQnapus1166 | UQnapus1177 | 0.95 |
|  | 9.72 | UQnapus1166 | UQnapus1180 | 0.98 |
|  | 9.72 | UQnapus1166 | UQnapus1186 | 0.73 |
|  | 9.72 | UQnapus1166 | UQnapus1197 | 0.73 |
|  | 9.72 | UQnapus1166 | UQnapus1198 | 0.73 |
|  | 9.72 | UQnapus1166 | UQnapus5111 | 0.73 |
|  | 9.72 | UQnapus1166 | UQnapus1202 | 0.71 |
|  | 9.72 | UQnapus1166 | UQnapus5331 | 0.73 |
|  | 9.72 | UQnapus1166 | UQnapus1205 | 0.73 |
|  | 9.72 | UQnapus1166 | UQnapus5510 | 0.75 |
|  | 9.72 | UQnapus1166 | UQnapus1234 | 0.77 |
|  | 9.72 | UQnapus1166 | UQnapus1570 | 0.73 |
|  | 9.72 | UQnapus1166 | UQnapus1933 | 0.77 |
|  | 9.72 | UQnapus1166 | UQnapus1938 | 0.75 |
|  | 9.72 | UQnapus1166 | UQnapus1939 | 0.77 |
|  | 9.72 | UQnapus1166 | UQnapus5604 | 0.73 |
|  | 9.72 | UQnapus1166 | UQnapus1940 | 0.74 |
|  | 9.72 | UQnapus1166 | UQnapus5381 | 0.73 |
|  | 9.72 | UQnapus1166 | UQnapus1946 | 0.73 |
|  | 9.72 | UQnapus1166 | UQnapus1947 | 0.73 |
|  | 9.72 | UQnapus1166 | UQnapus1948 | 0.73 |
|  | 9.72 | UQnapus1166 | UQnapus1949 | 0.73 |
|  | 9.72 | UQnapus1166 | UQnapus1952 | 0.73 |
|  | 9.72 | UQnapus1166 | UQnapus5739 | 0.73 |
|  | 9.72 | UQnapus1166 | UQnapus5497 | 0.73 |
|  | 9.72 | UQnapus1166 | UQnapus1137 | 0.75 |
|  | 9.72 | UQnapus1166 | UQnapus1138 | 0.80 |
|  | 9.72 | UQnapus1166 | UQnapus5183 | 0.80 |
|  | 9.72 | UQnapus1166 | UQnapus1145 | 0.79 |
|  | 9.72 | UQnapus1166 | UQnapus1147 | 0.85 |
|  | 9.72 | UQnapus1166 | UQnapus5365 | 0.85 |
|  | 9.72 | UQnapus1166 | UQnapus4827 | 0.87 |
|  | 9.72 | UQnapus1166 | UQnapus1151 | 0.87 |
|  | 9.72 | UQnapus1166 | UQnapus5268 | 0.87 |
|  | 9.72 | UQnapus1166 | UQnapus4633 | 0.86 |
|  | 9.72 | UQnapus1166 | UQnapus1154 | 0.83 |
|  | 9.72 | UQnapus1166 | UQnapus1155 | 0.83 |
|  | 9.72 | UQnapus1166 | UQnapus1156 | 0.83 |
|  | 9.72 | UQnapus1166 | UQnapus5269 | 0.87 |
|  | 9.72 | UQnapus1166 | UQnapus1158 | 0.87 |
|  | 9.72 | UQnapus1166 | UQnapus1161 | 0.83 |
|  | 8.80 | UQnapus0689 | UQnapus0689 | 1.00 |
|  | 8.80 | UQnapus0689 | UQnapus0927 | 0.79 |
|  | 8.80 | UQnapus0689 | UQnapus1046 | 0.74 |
|  | 8.80 | UQnapus0689 | UQnapus4000 | 0.75 |
|  | 8.80 | UQnapus0689 | UQnapus4718 | 0.76 |
|  | 8.80 | UQnapus0689 | UQnapus4009 | 0.89 |
|  | 8.80 | UQnapus0689 | UQnapus4011 | 0.87 |
|  | 8.80 | UQnapus0689 | UQnapus4012 | 0.89 |
|  | 8.80 | UQnapus0689 | UQnapus4014 | 0.85 |
|  | 8.80 | UQnapus0689 | UQnapus4016 | 0.85 |
|  | 8.80 | UQnapus0689 | UQnapus4018 | 0.87 |
|  | 8.80 | UQnapus0689 | UQnapus4021 | 0.85 |
|  | 8.80 | UQnapus0689 | UQnapus4058 | 0.96 |
|  | 8.80 | UQnapus0689 | UQnapus4059 | 0.96 |
|  | 8.48 | UQnapus1022 | UQnapus1022 | 1.00 |
|  | 8.48 | UQnapus1022 | UQnapus5494 | 0.86 |
|  | 8.38 | UQnapus0907 | UQnapus0907 | 1.00 |
|  | 8.38 | UQnapus0907 | UQnapus4278 | 0.71 |
|  | 8.22 | UQnapus4075 | UQnapus4075 | 1.00 |
|  | 8.22 | UQnapus4075 | UQnapus0406 | 0.91 |
|  | 8.22 | UQnapus4075 | UQnapus4065 | 0.85 |
|  | 8.10 | UQnapus1644 | UQnapus1644 | 1.00 |
|  | 8.10 | UQnapus1644 | UQnapus1647 | 0.90 |
|  | 8.10 | UQnapus1644 | UQnapus5037 | 0.90 |
|  | 8.10 | UQnapus1644 | UQnapus5038 | 0.90 |
|  | 8.10 | UQnapus1644 | UQnapus1652 | 0.77 |
|  | 8.10 | UQnapus1644 | UQnapus1653 | 0.90 |
|  | 8.10 | UQnapus1644 | UQnapus5903 | 0.92 |
|  | 8.10 | UQnapus1644 | UQnapus1664 | 0.71 |
|  | 8.10 | UQnapus1644 | UQnapus1665 | 0.81 |
|  | 8.10 | UQnapus1644 | UQnapus1667 | 0.71 |
|  | 8.10 | UQnapus1644 | UQnapus1672 | 0.81 |
|  | 8.10 | UQnapus1644 | UQnapus0237 | 0.80 |
|  | 8.10 | UQnapus1644 | UQnapus2993 | 0.88 |
|  | 8.10 | UQnapus1644 | UQnapus1642 | 0.88 |
|  | 8.10 | UQnapus1644 | UQnapus1643 | 0.90 |
|  | 7.91 | UQnapus1709 | UQnapus1709 | 1.00 |
|  | 7.91 | UQnapus1709 | UQnapus1710 | 0.97 |
|  | 7.91 | UQnapus1709 | UQnapus1718 | 0.88 |
|  | 7.91 | UQnapus1709 | UQnapus1722 | 0.86 |
|  | 7.91 | UQnapus1709 | UQnapus5448 | 0.85 |
|  | 7.91 | UQnapus1709 | UQnapus1725 | 0.83 |
|  | 7.91 | UQnapus1709 | UQnapus1726 | 0.83 |
|  | 7.91 | UQnapus1709 | UQnapus1729 | 0.74 |
|  | 7.91 | UQnapus1709 | UQnapus1730 | 0.74 |
|  | 7.91 | UQnapus1709 | UQnapus1737 | 0.72 |
|  | 7.91 | UQnapus1709 | UQnapus1738 | 0.79 |
|  | 7.91 | UQnapus1709 | UQnapus1744 | 0.81 |
|  | 7.91 | UQnapus1709 | UQnapus0092 | 0.88 |
|  | 7.91 | UQnapus1709 | UQnapus0098 | 0.86 |
|  | 7.91 | UQnapus1709 | UQnapus1839 | 0.88 |
|  | 7.91 | UQnapus1709 | UQnapus1843 | 0.83 |
|  | 7.91 | UQnapus1709 | UQnapus5914 | 0.83 |
|  | 7.91 | UQnapus1709 | UQnapus5084 | 0.83 |
|  | 7.91 | UQnapus1709 | UQnapus1845 | 0.81 |
|  | 7.91 | UQnapus1709 | UQnapus1846 | 0.83 |
|  | 7.91 | UQnapus1709 | UQnapus1851 | 0.76 |
|  | 7.91 | UQnapus1709 | UQnapus5590 | 0.79 |
|  | 7.91 | UQnapus1709 | UQnapus1853 | 0.79 |
|  | 7.91 | UQnapus1709 | UQnapus5337 | 0.74 |
|  | 7.91 | UQnapus1709 | UQnapus2719 | 0.86 |
|  | 7.91 | UQnapus1709 | UQnapus5708 | 0.86 |
|  | 7.91 | UQnapus1709 | UQnapus4687 | 0.74 |
|  | 7.91 | UQnapus1709 | UQnapus1693 | 0.74 |
|  | 7.91 | UQnapus1709 | UQnapus1700 | 0.88 |
|  | 7.91 | UQnapus1709 | UQnapus1702 | 0.88 |
|  | 7.91 | UQnapus1709 | UQnapus5281 | 0.88 |
|  | 7.91 | UQnapus1709 | UQnapus1704 | 0.88 |
|  | 7.91 | UQnapus1709 | UQnapus1705 | 0.88 |
|  | 7.91 | UQnapus1709 | UQnapus5374 | 0.93 |
|  | 7.32 | UQnapus1298 | UQnapus1298 | 1.00 |
|  | 7.30 | UQnapus4099 | UQnapus4099 | 1.00 |
|  | 7.30 | UQnapus4099 | UQnapus4100 | 0.96 |
|  | 7.30 | UQnapus4099 | UQnapus0624 | 0.77 |
|  | 7.02 | UQnapus0772 | UQnapus0772 | 1.00 |
|  | 6.62 | UQnapus1789 | UQnapus1789 | 1.00 |
|  | 6.62 | UQnapus1789 | UQnapus5584 | 0.95 |
|  | 6.62 | UQnapus1789 | UQnapus1796 | 0.76 |
|  | 6.62 | UQnapus1789 | UQnapus0203 | 0.73 |
|  | 6.25 | UQnapus5819 | UQnapus5819 | 1.00 |
|  | 6.25 | UQnapus5819 | UQnapus1109 | 0.81 |
|  | 5.95 | UQnapus0238 | UQnapus0238 | 1.00 |
|  | 5.95 | UQnapus0238 | UQnapus2993 | 0.76 |
|  | 5.95 | UQnapus0238 | UQnapus1647 | 0.77 |
|  | 5.95 | UQnapus0238 | UQnapus5037 | 0.77 |
|  | 5.95 | UQnapus0238 | UQnapus5038 | 0.76 |
|  | 5.95 | UQnapus0238 | UQnapus1653 | 0.75 |
|  | 5.95 | UQnapus0238 | UQnapus5903 | 0.75 |
|  | 5.95 | UQnapus0238 | UQnapus1664 | 0.76 |
|  | 5.95 | UQnapus0238 | UQnapus1665 | 0.84 |
|  | 5.95 | UQnapus0238 | UQnapus1667 | 0.76 |
|  | 5.95 | UQnapus0238 | UQnapus1672 | 0.88 |
|  | 5.95 | UQnapus0238 | UQnapus1676 | 0.80 |
|  | 5.95 | UQnapus0238 | UQnapus1677 | 0.77 |
|  | 5.95 | UQnapus0238 | UQnapus1680 | 0.78 |
|  | 5.95 | UQnapus0238 | UQnapus1681 | 0.76 |
|  | 5.95 | UQnapus0238 | UQnapus5575 | 0.71 |
|  | 5.95 | UQnapus0238 | UQnapus4687 | 0.75 |
|  | 5.95 | UQnapus0238 | UQnapus0057 | 0.75 |
|  | 5.95 | UQnapus0238 | UQnapus1693 | 0.73 |
|  | 5.87 | UQnapus3837 | UQnapus3837 | 1.00 |
|  | 5.87 | UQnapus3837 | UQnapus4537 | 0.83 |
|  | 5.87 | UQnapus3837 | UQnapus3186 | 0.81 |
|  | 5.87 | UQnapus3837 | UQnapus4890 | 0.85 |
|  | 5.87 | UQnapus3837 | UQnapus3833 | 0.89 |
|  | 5.80 | UQnapus5471 | UQnapus5471 | 1.00 |
|  | 5.80 | UQnapus5471 | UQnapus2985 | 0.87 |
|  | 5.80 | UQnapus5471 | UQnapus0874 | 0.83 |
|  | 5.80 | UQnapus5471 | UQnapus0224 | 0.72 |
|  | 5.80 | UQnapus5471 | UQnapus2970 | 0.72 |
|  | 5.80 | UQnapus5471 | UQnapus2980 | 0.83 |
|  | 5.80 | UQnapus5471 | UQnapus5751 | 0.83 |
|  | 5.80 | UQnapus5471 | UQnapus2983 | 0.91 |
|  | 5.73 | UQnapus5554 | UQnapus5554 | 1.00 |
|  | 5.73 | UQnapus5554 | UQnapus1487 | 0.79 |
|  | 5.73 | UQnapus5554 | UQnapus5556 | 0.76 |
|  | 5.73 | UQnapus5554 | UQnapus1490 | 0.83 |
|  | 5.73 | UQnapus5554 | UQnapus2769 | 0.79 |
|  | 5.73 | UQnapus5554 | UQnapus1472 | 0.87 |
|  | 5.73 | UQnapus5554 | UQnapus5552 | 0.87 |
|  | 5.70 | UQnapus3842 | UQnapus3842 | 1.00 |
|  | 5.70 | UQnapus3842 | UQnapus3853 | 0.93 |
|  | 5.70 | UQnapus3842 | UQnapus4502 | 1.00 |
|  | 5.70 | UQnapus3842 | UQnapus5067 | 1.00 |
|  | 5.70 | UQnapus3842 | UQnapus0888 | 0.93 |
|  | 5.45 | UQnapus1545 | UQnapus1545 | 1.00 |
|  | 5.45 | UQnapus1545 | UQnapus1546 | 0.95 |
|  | 5.45 | UQnapus1545 | UQnapus1547 | 0.88 |
|  | 5.45 | UQnapus1545 | UQnapus1548 | 0.84 |
|  | 5.45 | UQnapus1545 | UQnapus5278 | 0.91 |
|  | 5.45 | UQnapus1545 | UQnapus1549 | 0.91 |
|  | 5.45 | UQnapus1545 | UQnapus1553 | 0.86 |
|  | 5.45 | UQnapus1545 | UQnapus1554 | 0.72 |
|  | 5.45 | UQnapus1545 | UQnapus4650 | 0.76 |
|  | 5.45 | UQnapus1545 | UQnapus1533 | 0.72 |
|  | 5.45 | UQnapus1545 | UQnapus1534 | 0.78 |
|  | 5.45 | UQnapus1545 | UQnapus1536 | 0.76 |
|  | 5.45 | UQnapus1545 | UQnapus1537 | 0.77 |
|  | 5.45 | UQnapus1545 | UQnapus1540 | 0.89 |
|  | 5.45 | UQnapus1545 | UQnapus5561 | 0.89 |
|  | 5.36 | UQnapus4044 | UQnapus4044 | 1.00 |
|  | 5.36 | UQnapus4044 | UQnapus4009 | 0.72 |
|  | 5.36 | UQnapus4044 | UQnapus4011 | 0.74 |
|  | 5.36 | UQnapus4044 | UQnapus4012 | 0.74 |
|  | 5.36 | UQnapus4044 | UQnapus4014 | 0.76 |
|  | 5.36 | UQnapus4044 | UQnapus4016 | 0.76 |
|  | 5.36 | UQnapus4044 | UQnapus4018 | 0.76 |
|  | 5.36 | UQnapus4044 | UQnapus4021 | 0.76 |
|  | 5.36 | UQnapus4044 | UQnapus4032 | 0.93 |
|  | 5.36 | UQnapus4044 | UQnapus4033 | 0.93 |
|  | 5.36 | UQnapus4044 | UQnapus4039 | 0.93 |
|  | 5.36 | UQnapus4044 | UQnapus4042 | 1.00 |
|  | 5.26 | UQnapus5307 | UQnapus5307 | 1.00 |
|  | 5.26 | UQnapus5307 | UQnapus2856 | 1.00 |
|  | 5.26 | UQnapus5307 | UQnapus2858 | 0.95 |
|  | 5.26 | UQnapus5307 | UQnapus2859 | 0.93 |
|  | 5.26 | UQnapus5307 | UQnapus2861 | 0.93 |
|  | 5.26 | UQnapus5307 | UQnapus2862 | 0.94 |
|  | 5.26 | UQnapus5307 | UQnapus2865 | 0.91 |
|  | 5.26 | UQnapus5307 | UQnapus2869 | 0.87 |
|  | 5.26 | UQnapus5307 | UQnapus2870 | 0.85 |
|  | 5.26 | UQnapus5307 | UQnapus2873 | 0.93 |
|  | 5.26 | UQnapus5307 | UQnapus2874 | 0.85 |
|  | 5.26 | UQnapus5307 | UQnapus2876 | 0.85 |
|  | 5.26 | UQnapus5307 | UQnapus2877 | 0.86 |
|  | 5.26 | UQnapus5307 | UQnapus2880 | 0.85 |
|  | 5.26 | UQnapus5307 | UQnapus2881 | 0.85 |
|  | 5.26 | UQnapus5307 | UQnapus2883 | 0.91 |
|  | 5.26 | UQnapus5307 | UQnapus4674 | 0.91 |
|  | 5.26 | UQnapus5307 | UQnapus2884 | 0.91 |
|  | 5.26 | UQnapus5307 | UQnapus5469 | 0.89 |
|  | 5.26 | UQnapus5307 | UQnapus2906 | 0.85 |
|  | 5.26 | UQnapus5307 | UQnapus2909 | 0.85 |
|  | 5.26 | UQnapus5307 | UQnapus0249 | 0.93 |
|  | 5.26 | UQnapus5307 | UQnapus0250 | 0.93 |
|  | 5.26 | UQnapus5307 | UQnapus0304 | 0.87 |
|  | 5.26 | UQnapus5307 | UQnapus0337 | 0.85 |
|  | 5.26 | UQnapus5307 | UQnapus0341 | 0.96 |
|  | 5.26 | UQnapus5307 | UQnapus2479 | 0.94 |
|  | 5.26 | UQnapus5307 | UQnapus2480 | 0.85 |
|  | 5.26 | UQnapus5307 | UQnapus2850 | 0.72 |
|  | 5.26 | UQnapus5307 | UQnapus2851 | 0.76 |
|  | 5.26 | UQnapus5307 | UQnapus2855 | 0.98 |
|  | 5.22 | UQnapus0743 | UQnapus0743 | 1.00 |
|  | 5.22 | UQnapus0743 | UQnapus0054 | 0.83 |
|  | 5.22 | UQnapus5902 | UQnapus5902 | 1.00 |
|  | 5.22 | UQnapus5902 | UQnapus5487 | 0.91 |
|  | 5.22 | UQnapus5902 | UQnapus5488 | 0.87 |
|  | 5.22 | UQnapus5902 | UQnapus1065 | 0.85 |
|  | 5.22 | UQnapus5902 | UQnapus1070 | 0.73 |
|  | 5.22 | UQnapus5902 | UQnapus1073 | 0.71 |
|  | 5.22 | UQnapus5902 | UQnapus5486 | 0.98 |
|  | 5.22 | UQnapus5902 | UQnapus1060 | 0.88 |
|  | 5.20 | UQnapus5988 | UQnapus5988 | 1.00 |
|  | 5.11 | UQnapus1279 | UQnapus1279 | 1.00 |
|  | 5.11 | UQnapus1279 | UQnapus5656 | 0.85 |
|  | 5.11 | UQnapus1279 | UQnapus0776 | 0.83 |
| East | 21.21 | UQnapus0669 | UQnapus0669 | 1.00 |
|  | 21.21 | UQnapus0669 | UQnapus0816 | 0.91 |
|  | 21.21 | UQnapus0669 | UQnapus4901 | 0.89 |
|  | 21.21 | UQnapus0669 | UQnapus0104 | 0.88 |
|  | 21.21 | UQnapus0669 | UQnapus4105 | 0.89 |
|  | 21.21 | UQnapus0669 | UQnapus4108 | 0.89 |
|  | 21.21 | UQnapus0669 | UQnapus4115 | 1.00 |
|  | 21.21 | UQnapus0669 | UQnapus4278 | 0.80 |
|  | 21.21 | UQnapus0669 | UQnapus0052 | 0.89 |
|  | 15.28 | UQnapus0863 | UQnapus0863 | 1.00 |
|  | 15.28 | UQnapus0863 | UQnapus0712 | 0.93 |
|  | 14.97 | UQnapus4153 | UQnapus4153 | 1.00 |
|  | 12.49 | UQnapus1644 | UQnapus1644 | 1.00 |
|  | 12.49 | UQnapus1644 | UQnapus1647 | 0.90 |
|  | 12.49 | UQnapus1644 | UQnapus5037 | 0.90 |
|  | 12.49 | UQnapus1644 | UQnapus5038 | 0.90 |
|  | 12.49 | UQnapus1644 | UQnapus1652 | 0.77 |
|  | 12.49 | UQnapus1644 | UQnapus1653 | 0.90 |
|  | 12.49 | UQnapus1644 | UQnapus5903 | 0.92 |
|  | 12.49 | UQnapus1644 | UQnapus1664 | 0.71 |
|  | 12.49 | UQnapus1644 | UQnapus1665 | 0.81 |
|  | 12.49 | UQnapus1644 | UQnapus1667 | 0.71 |
|  | 12.49 | UQnapus1644 | UQnapus1672 | 0.81 |
|  | 12.49 | UQnapus1644 | UQnapus0237 | 0.80 |
|  | 12.49 | UQnapus1644 | UQnapus2993 | 0.88 |
|  | 12.49 | UQnapus1644 | UQnapus1642 | 0.88 |
|  | 12.49 | UQnapus1644 | UQnapus1643 | 0.90 |
|  | 11.49 | UQnapus1709 | UQnapus1709 | 1.00 |
|  | 11.49 | UQnapus1709 | UQnapus1710 | 0.97 |
|  | 11.49 | UQnapus1709 | UQnapus1718 | 0.88 |
|  | 11.49 | UQnapus1709 | UQnapus1722 | 0.86 |
|  | 11.49 | UQnapus1709 | UQnapus5448 | 0.85 |
|  | 11.49 | UQnapus1709 | UQnapus1725 | 0.83 |
|  | 11.49 | UQnapus1709 | UQnapus1726 | 0.83 |
|  | 11.49 | UQnapus1709 | UQnapus1729 | 0.74 |
|  | 11.49 | UQnapus1709 | UQnapus1730 | 0.74 |
|  | 11.49 | UQnapus1709 | UQnapus1737 | 0.72 |
|  | 11.49 | UQnapus1709 | UQnapus1738 | 0.79 |
|  | 11.49 | UQnapus1709 | UQnapus1744 | 0.81 |
|  | 11.49 | UQnapus1709 | UQnapus0092 | 0.88 |
|  | 11.49 | UQnapus1709 | UQnapus0098 | 0.86 |
|  | 11.49 | UQnapus1709 | UQnapus1839 | 0.88 |
|  | 11.49 | UQnapus1709 | UQnapus1843 | 0.83 |
|  | 11.49 | UQnapus1709 | UQnapus5914 | 0.83 |
|  | 11.49 | UQnapus1709 | UQnapus5084 | 0.83 |
|  | 11.49 | UQnapus1709 | UQnapus1845 | 0.81 |
|  | 11.49 | UQnapus1709 | UQnapus1846 | 0.83 |
|  | 11.49 | UQnapus1709 | UQnapus1851 | 0.76 |
|  | 11.49 | UQnapus1709 | UQnapus5590 | 0.79 |
|  | 11.49 | UQnapus1709 | UQnapus1853 | 0.79 |
|  | 11.49 | UQnapus1709 | UQnapus5337 | 0.74 |
|  | 11.49 | UQnapus1709 | UQnapus2719 | 0.86 |
|  | 11.49 | UQnapus1709 | UQnapus5708 | 0.86 |
|  | 11.49 | UQnapus1709 | UQnapus4687 | 0.74 |
|  | 11.49 | UQnapus1709 | UQnapus1693 | 0.74 |
|  | 11.49 | UQnapus1709 | UQnapus1700 | 0.88 |
|  | 11.49 | UQnapus1709 | UQnapus1702 | 0.88 |
|  | 11.49 | UQnapus1709 | UQnapus5281 | 0.88 |
|  | 11.49 | UQnapus1709 | UQnapus1704 | 0.88 |
|  | 11.49 | UQnapus1709 | UQnapus1705 | 0.88 |
|  | 11.49 | UQnapus1709 | UQnapus5374 | 0.93 |
|  | 11.11 | UQnapus0238 | UQnapus0238 | 1.00 |
|  | 11.11 | UQnapus0238 | UQnapus2993 | 0.76 |
|  | 11.11 | UQnapus0238 | UQnapus1647 | 0.77 |
|  | 11.11 | UQnapus0238 | UQnapus5037 | 0.77 |
|  | 11.11 | UQnapus0238 | UQnapus5038 | 0.76 |
|  | 11.11 | UQnapus0238 | UQnapus1653 | 0.75 |
|  | 11.11 | UQnapus0238 | UQnapus5903 | 0.75 |
|  | 11.11 | UQnapus0238 | UQnapus1664 | 0.76 |
|  | 11.11 | UQnapus0238 | UQnapus1665 | 0.84 |
|  | 11.11 | UQnapus0238 | UQnapus1667 | 0.76 |
|  | 11.11 | UQnapus0238 | UQnapus1672 | 0.88 |
|  | 11.11 | UQnapus0238 | UQnapus1676 | 0.80 |
|  | 11.11 | UQnapus0238 | UQnapus1677 | 0.77 |
|  | 11.11 | UQnapus0238 | UQnapus1680 | 0.78 |
|  | 11.11 | UQnapus0238 | UQnapus1681 | 0.76 |
|  | 11.11 | UQnapus0238 | UQnapus5575 | 0.71 |
|  | 11.11 | UQnapus0238 | UQnapus4687 | 0.75 |
|  | 11.11 | UQnapus0238 | UQnapus0057 | 0.75 |
|  | 11.11 | UQnapus0238 | UQnapus1693 | 0.73 |
|  | 11.00 | UQnapus0907 | UQnapus0907 | 1.00 |
|  | 11.00 | UQnapus0907 | UQnapus4278 | 0.71 |
|  | 10.24 | UQnapus4219 | UQnapus4219 | 1.00 |
|  | 10.24 | UQnapus4219 | UQnapus4221 | 1.00 |
|  | 10.24 | UQnapus4219 | UQnapus4401 | 0.73 |
|  | 10.24 | UQnapus4219 | UQnapus4711 | 0.83 |
|  | 10.24 | UQnapus4219 | UQnapus3764 | 0.84 |
|  | 10.24 | UQnapus4219 | UQnapus3765 | 0.84 |
|  | 10.24 | UQnapus4219 | UQnapus3767 | 0.84 |
|  | 10.24 | UQnapus4219 | UQnapus0144 | 0.85 |
|  | 10.24 | UQnapus4219 | UQnapus4218 | 1.00 |
|  | 9.42 | UQnapus1298 | UQnapus1298 | 1.00 |
|  | 8.29 | UQnapus1789 | UQnapus1789 | 1.00 |
|  | 8.29 | UQnapus1789 | UQnapus5584 | 0.95 |
|  | 8.29 | UQnapus1789 | UQnapus1796 | 0.76 |
|  | 8.29 | UQnapus1789 | UQnapus0203 | 0.73 |
|  | 8.15 | UQnapus5471 | UQnapus5471 | 1.00 |
|  | 8.15 | UQnapus5471 | UQnapus2985 | 0.87 |
|  | 8.15 | UQnapus5471 | UQnapus0874 | 0.83 |
|  | 8.15 | UQnapus5471 | UQnapus0224 | 0.72 |
|  | 8.15 | UQnapus5471 | UQnapus2970 | 0.72 |
|  | 8.15 | UQnapus5471 | UQnapus2980 | 0.83 |
|  | 8.15 | UQnapus5471 | UQnapus5751 | 0.83 |
|  | 8.15 | UQnapus5471 | UQnapus2983 | 0.91 |
|  | 7.89 | UQnapus1970 | UQnapus1970 | 1.00 |
|  | 7.89 | UQnapus1970 | UQnapus1972 | 0.75 |
|  | 7.89 | UQnapus1970 | UQnapus5385 | 0.87 |
|  | 7.89 | UQnapus1970 | UQnapus1984 | 0.87 |
|  | 7.89 | UQnapus1970 | UQnapus0827 | 0.87 |
|  | 7.89 | UQnapus1970 | UQnapus1956 | 0.78 |
|  | 7.89 | UQnapus1970 | UQnapus1958 | 0.76 |
|  | 7.89 | UQnapus1970 | UQnapus1959 | 0.85 |
|  | 7.89 | UQnapus1970 | UQnapus1960 | 0.85 |
|  | 7.89 | UQnapus1970 | UQnapus5383 | 0.89 |
|  | 7.89 | UQnapus1970 | UQnapus1966 | 0.91 |
|  | 7.89 | UQnapus1970 | UQnapus5384 | 0.91 |
|  | 7.86 | UQnapus1166 | UQnapus1166 | 1.00 |
|  | 7.86 | UQnapus1166 | UQnapus5500 | 0.77 |
|  | 7.86 | UQnapus1166 | UQnapus1172 | 0.83 |
|  | 7.86 | UQnapus1166 | UQnapus1177 | 0.95 |
|  | 7.86 | UQnapus1166 | UQnapus1180 | 0.98 |
|  | 7.86 | UQnapus1166 | UQnapus1186 | 0.73 |
|  | 7.86 | UQnapus1166 | UQnapus1197 | 0.73 |
|  | 7.86 | UQnapus1166 | UQnapus1198 | 0.73 |
|  | 7.86 | UQnapus1166 | UQnapus5111 | 0.73 |
|  | 7.86 | UQnapus1166 | UQnapus1202 | 0.71 |
|  | 7.86 | UQnapus1166 | UQnapus5331 | 0.73 |
|  | 7.86 | UQnapus1166 | UQnapus1205 | 0.73 |
|  | 7.86 | UQnapus1166 | UQnapus5510 | 0.75 |
|  | 7.86 | UQnapus1166 | UQnapus1234 | 0.77 |
|  | 7.86 | UQnapus1166 | UQnapus1570 | 0.73 |
|  | 7.86 | UQnapus1166 | UQnapus1933 | 0.77 |
|  | 7.86 | UQnapus1166 | UQnapus1938 | 0.75 |
|  | 7.86 | UQnapus1166 | UQnapus1939 | 0.77 |
|  | 7.86 | UQnapus1166 | UQnapus5604 | 0.73 |
|  | 7.86 | UQnapus1166 | UQnapus1940 | 0.74 |
|  | 7.86 | UQnapus1166 | UQnapus5381 | 0.73 |
|  | 7.86 | UQnapus1166 | UQnapus1946 | 0.73 |
|  | 7.86 | UQnapus1166 | UQnapus1947 | 0.73 |
|  | 7.86 | UQnapus1166 | UQnapus1948 | 0.73 |
|  | 7.86 | UQnapus1166 | UQnapus1949 | 0.73 |
|  | 7.86 | UQnapus1166 | UQnapus1952 | 0.73 |
|  | 7.86 | UQnapus1166 | UQnapus5739 | 0.73 |
|  | 7.86 | UQnapus1166 | UQnapus5497 | 0.73 |
|  | 7.86 | UQnapus1166 | UQnapus1137 | 0.75 |
|  | 7.86 | UQnapus1166 | UQnapus1138 | 0.80 |
|  | 7.86 | UQnapus1166 | UQnapus5183 | 0.80 |
|  | 7.86 | UQnapus1166 | UQnapus1145 | 0.79 |
|  | 7.86 | UQnapus1166 | UQnapus1147 | 0.85 |
|  | 7.86 | UQnapus1166 | UQnapus5365 | 0.85 |
|  | 7.86 | UQnapus1166 | UQnapus4827 | 0.87 |
|  | 7.86 | UQnapus1166 | UQnapus1151 | 0.87 |
|  | 7.86 | UQnapus1166 | UQnapus5268 | 0.87 |
|  | 7.86 | UQnapus1166 | UQnapus4633 | 0.86 |
|  | 7.86 | UQnapus1166 | UQnapus1154 | 0.83 |
|  | 7.86 | UQnapus1166 | UQnapus1155 | 0.83 |
|  | 7.86 | UQnapus1166 | UQnapus1156 | 0.83 |
|  | 7.86 | UQnapus1166 | UQnapus5269 | 0.87 |
|  | 7.86 | UQnapus1166 | UQnapus1158 | 0.87 |
|  | 7.86 | UQnapus1166 | UQnapus1161 | 0.83 |
|  | 7.42 | UQnapus1279 | UQnapus1279 | 1.00 |
|  | 7.42 | UQnapus1279 | UQnapus5656 | 0.85 |
|  | 7.42 | UQnapus1279 | UQnapus0776 | 0.83 |
|  | 7.38 | UQnapus5441 | UQnapus5441 | 1.00 |
|  | 7.38 | UQnapus5441 | UQnapus5086 | 0.95 |
|  | 7.04 | UQnapus4686 | UQnapus4686 | 1.00 |
|  | 7.04 | UQnapus4686 | UQnapus5656 | 0.83 |
|  | 7.04 | UQnapus4686 | UQnapus0776 | 0.85 |
|  | 7.04 | UQnapus4686 | UQnapus1222 | 0.71 |
|  | 7.04 | UQnapus4686 | UQnapus1243 | 0.71 |
|  | 7.04 | UQnapus4686 | UQnapus1244 | 0.81 |
|  | 7.04 | UQnapus4686 | UQnapus5184 | 0.80 |
|  | 7.04 | UQnapus4686 | UQnapus1245 | 0.78 |
|  | 7.04 | UQnapus4686 | UQnapus1248 | 0.79 |
|  | 7.04 | UQnapus4686 | UQnapus1250 | 0.78 |
|  | 7.01 | UQnapus0895 | UQnapus0895 | 1.00 |
|  | 7.01 | UQnapus0895 | UQnapus5991 | 0.85 |
|  | 7.01 | UQnapus0895 | UQnapus1214 | 0.81 |
|  | 7.01 | UQnapus0895 | UQnapus1470 | 0.87 |
|  | 7.01 | UQnapus0895 | UQnapus0860 | 0.89 |
|  | 6.59 | UQnapus0394 | UQnapus0394 | 1.00 |
|  | 6.46 | UQnapus3319 | UQnapus3319 | 1.00 |
|  | 6.46 | UQnapus3319 | UQnapus3320 | 1.00 |
|  | 6.46 | UQnapus3319 | UQnapus3324 | 1.00 |
|  | 6.46 | UQnapus3319 | UQnapus3325 | 1.00 |
|  | 6.46 | UQnapus3319 | UQnapus5841 | 1.00 |
|  | 6.46 | UQnapus3319 | UQnapus3327 | 1.00 |
|  | 6.46 | UQnapus3319 | UQnapus3328 | 1.00 |
|  | 6.46 | UQnapus3319 | UQnapus5437 | 1.00 |
|  | 6.46 | UQnapus3319 | UQnapus3335 | 1.00 |
|  | 6.46 | UQnapus3319 | UQnapus3342 | 0.85 |
|  | 6.46 | UQnapus3319 | UQnapus5879 | 0.88 |
|  | 6.46 | UQnapus3319 | UQnapus3407 | 0.90 |
|  | 6.46 | UQnapus3319 | UQnapus3760 | 0.79 |
|  | 6.46 | UQnapus3319 | UQnapus0532 | 1.00 |
|  | 6.46 | UQnapus3319 | UQnapus0553 | 0.77 |
|  | 6.46 | UQnapus3319 | UQnapus0574 | 0.85 |
|  | 6.46 | UQnapus3319 | UQnapus0605 | 0.79 |
|  | 6.46 | UQnapus3319 | UQnapus0665 | 1.00 |
|  | 6.46 | UQnapus3319 | UQnapus4934 | 0.90 |
|  | 6.46 | UQnapus3319 | UQnapus0701 | 1.00 |
|  | 6.46 | UQnapus3319 | UQnapus0792 | 0.84 |
|  | 6.46 | UQnapus3319 | UQnapus0832 | 1.00 |
|  | 6.46 | UQnapus3319 | UQnapus0920 | 0.86 |
|  | 6.46 | UQnapus3319 | UQnapus0969 | 0.81 |
|  | 6.46 | UQnapus3319 | UQnapus0988 | 0.74 |
|  | 6.46 | UQnapus3319 | UQnapus1032 | 0.86 |
|  | 6.46 | UQnapus3319 | UQnapus3241 | 0.78 |
|  | 6.46 | UQnapus3319 | UQnapus3243 | 0.86 |
|  | 6.46 | UQnapus3319 | UQnapus3310 | 1.00 |
|  | 6.46 | UQnapus3319 | UQnapus3312 | 1.00 |
|  | 6.46 | UQnapus3319 | UQnapus3316 | 1.00 |
|  | 6.41 | UQnapus4810 | UQnapus4810 | 1.00 |
|  | 6.41 | UQnapus4810 | UQnapus4818 | 0.72 |
|  | 6.41 | UQnapus4810 | UQnapus4366 | 0.93 |
|  | 6.41 | UQnapus4810 | UQnapus4367 | 0.93 |
|  | 6.41 | UQnapus4810 | UQnapus4371 | 0.91 |
|  | 6.41 | UQnapus4810 | UQnapus4372 | 0.91 |
|  | 6.41 | UQnapus4810 | UQnapus4373 | 0.91 |
|  | 6.41 | UQnapus4810 | UQnapus4375 | 0.88 |
|  | 6.41 | UQnapus4810 | UQnapus4377 | 0.87 |
|  | 6.41 | UQnapus4810 | UQnapus4379 | 0.83 |
|  | 6.41 | UQnapus4810 | UQnapus4380 | 0.83 |
|  | 6.41 | UQnapus4810 | UQnapus4390 | 0.79 |
|  | 6.41 | UQnapus4810 | UQnapus4397 | 0.80 |
|  | 6.16 | UQnapus2347 | UQnapus2347 | 1.00 |
|  | 6.16 | UQnapus2347 | UQnapus2348 | 0.86 |
|  | 6.16 | UQnapus2347 | UQnapus5172 | 0.77 |
|  | 6.16 | UQnapus2347 | UQnapus2342 | 0.77 |
|  | 6.16 | UQnapus2347 | UQnapus2346 | 0.84 |
|  | 6.03 | UQnapus3842 | UQnapus3842 | 1.00 |
|  | 6.03 | UQnapus3842 | UQnapus3853 | 0.93 |
|  | 6.03 | UQnapus3842 | UQnapus4502 | 1.00 |
|  | 6.03 | UQnapus3842 | UQnapus5067 | 1.00 |
|  | 6.03 | UQnapus3842 | UQnapus0888 | 0.93 |
|  | 6.00 | UQnapus0689 | UQnapus0689 | 1.00 |
|  | 6.00 | UQnapus0689 | UQnapus0927 | 0.79 |
|  | 6.00 | UQnapus0689 | UQnapus1046 | 0.74 |
|  | 6.00 | UQnapus0689 | UQnapus4000 | 0.75 |
|  | 6.00 | UQnapus0689 | UQnapus4718 | 0.76 |
|  | 6.00 | UQnapus0689 | UQnapus4009 | 0.89 |
|  | 6.00 | UQnapus0689 | UQnapus4011 | 0.87 |
|  | 6.00 | UQnapus0689 | UQnapus4012 | 0.89 |
|  | 6.00 | UQnapus0689 | UQnapus4014 | 0.85 |
|  | 6.00 | UQnapus0689 | UQnapus4016 | 0.85 |
|  | 6.00 | UQnapus0689 | UQnapus4018 | 0.87 |
|  | 6.00 | UQnapus0689 | UQnapus4021 | 0.85 |
|  | 6.00 | UQnapus0689 | UQnapus4058 | 0.96 |
|  | 6.00 | UQnapus0689 | UQnapus4059 | 0.96 |
|  | 5.99 | UQnapus4075 | UQnapus4075 | 1.00 |
|  | 5.99 | UQnapus4075 | UQnapus0406 | 0.91 |
|  | 5.99 | UQnapus4075 | UQnapus4065 | 0.85 |
|  | 5.71 | UQnapus3878 | UQnapus3878 | 1.00 |
|  | 5.71 | UQnapus3878 | UQnapus0594 | 0.87 |
|  | 5.71 | UQnapus3878 | UQnapus0604 | 0.85 |
|  | 5.71 | UQnapus3878 | UQnapus0806 | 0.87 |
|  | 5.71 | UQnapus3878 | UQnapus0896 | 0.87 |
|  | 5.71 | UQnapus3878 | UQnapus0911 | 0.85 |
|  | 5.71 | UQnapus3878 | UQnapus0974 | 0.97 |
|  | 5.71 | UQnapus3878 | UQnapus0351 | 1.00 |
|  | 5.71 | UQnapus3878 | UQnapus3149 | 0.87 |
|  | 5.71 | UQnapus3878 | UQnapus3875 | 1.00 |
|  | 5.71 | UQnapus3878 | UQnapus3876 | 1.00 |
|  | 5.71 | UQnapus3878 | UQnapus4817 | 1.00 |
|  | 5.71 | UQnapus3878 | UQnapus3877 | 1.00 |
|  | 5.58 | UQnapus1427 | UQnapus1427 | 1.00 |
|  | 5.58 | UQnapus1427 | UQnapus5541 | 0.98 |
|  | 5.58 | UQnapus1427 | UQnapus5543 | 0.88 |
|  | 5.58 | UQnapus1427 | UQnapus1445 | 0.76 |
|  | 5.58 | UQnapus1427 | UQnapus1450 | 0.70 |
|  | 5.58 | UQnapus1427 | UQnapus1451 | 0.70 |
|  | 5.58 | UQnapus1427 | UQnapus5627 | 0.70 |
|  | 5.58 | UQnapus1427 | UQnapus2134 | 0.73 |
|  | 5.58 | UQnapus1427 | UQnapus5051 | 0.74 |
|  | 5.58 | UQnapus1427 | UQnapus2839 | 0.83 |
|  | 5.58 | UQnapus1427 | UQnapus5055 | 0.83 |
|  | 5.58 | UQnapus1427 | UQnapus3572 | 0.95 |
|  | 5.58 | UQnapus1427 | UQnapus4885 | 1.00 |
|  | 5.58 | UQnapus1427 | UQnapus0619 | 0.74 |
|  | 5.58 | UQnapus1427 | UQnapus0309 | 0.78 |
|  | 5.58 | UQnapus1427 | UQnapus1405 | 0.70 |
|  | 5.58 | UQnapus1427 | UQnapus5852 | 0.91 |
|  | 5.58 | UQnapus1427 | UQnapus1424 | 0.93 |
|  | 5.58 | UQnapus1427 | UQnapus1425 | 0.91 |
|  | 5.47 | UQnapus1450 | UQnapus1450 | 1.00 |
|  | 5.47 | UQnapus1450 | UQnapus1451 | 0.95 |
|  | 5.47 | UQnapus1450 | UQnapus5578 | 0.80 |
|  | 5.47 | UQnapus1450 | UQnapus4987 | 1.00 |
|  | 5.47 | UQnapus1450 | UQnapus3513 | 0.76 |
|  | 5.47 | UQnapus1450 | UQnapus0293 | 0.81 |
|  | 5.47 | UQnapus1450 | UQnapus1378 | 0.73 |
|  | 5.47 | UQnapus1450 | UQnapus1388 | 0.72 |
|  | 5.47 | UQnapus1450 | UQnapus1394 | 0.75 |
|  | 5.47 | UQnapus1450 | UQnapus1395 | 0.74 |
|  | 5.47 | UQnapus1450 | UQnapus0097 | 0.74 |
|  | 5.47 | UQnapus1450 | UQnapus0125 | 0.76 |
|  | 5.47 | UQnapus1450 | UQnapus1398 | 0.76 |
|  | 5.47 | UQnapus1450 | UQnapus1399 | 0.74 |
|  | 5.47 | UQnapus1450 | UQnapus1405 | 0.75 |
|  | 5.47 | UQnapus1450 | UQnapus5852 | 0.78 |
|  | 5.47 | UQnapus1450 | UQnapus1424 | 0.72 |
|  | 5.47 | UQnapus1450 | UQnapus1427 | 0.70 |
|  | 5.47 | UQnapus1450 | UQnapus1441 | 0.84 |
|  | 5.47 | UQnapus1450 | UQnapus1442 | 0.86 |
|  | 5.47 | UQnapus1450 | UQnapus1445 | 0.89 |
|  | 5.47 | UQnapus1450 | UQnapus5166 | 0.82 |
|  | 5.44 | UQnapus2989 | UQnapus2989 | 1.00 |
|  | 5.44 | UQnapus2989 | UQnapus3003 | 0.78 |
|  | 5.44 | UQnapus2989 | UQnapus5758 | 0.78 |
|  | 5.44 | UQnapus2989 | UQnapus3004 | 0.77 |
|  | 5.44 | UQnapus2989 | UQnapus3007 | 0.82 |
|  | 5.44 | UQnapus2989 | UQnapus2973 | 0.89 |
|  | 5.44 | UQnapus2989 | UQnapus5749 | 0.89 |
|  | 5.44 | UQnapus2989 | UQnapus2977 | 0.84 |
|  | 5.44 | UQnapus2989 | UQnapus5750 | 0.84 |
|  | 5.44 | UQnapus2989 | UQnapus2987 | 0.91 |
|  | 5.41 | UQnapus0390 | UQnapus0390 | 1.00 |
|  | 5.41 | UQnapus0390 | UQnapus1819 | 0.79 |
|  | 5.41 | UQnapus0390 | UQnapus1822 | 0.89 |
|  | 5.41 | UQnapus0390 | UQnapus1827 | 0.89 |
|  | 5.41 | UQnapus0390 | UQnapus1828 | 0.82 |
|  | 5.41 | UQnapus0390 | UQnapus1830 | 0.93 |
|  | 5.41 | UQnapus0390 | UQnapus1831 | 0.94 |
|  | 5.41 | UQnapus0390 | UQnapus5588 | 0.96 |
|  | 5.41 | UQnapus0390 | UQnapus1832 | 0.83 |
|  | 5.41 | UQnapus0390 | UQnapus1833 | 0.96 |
|  | 5.41 | UQnapus0390 | UQnapus5913 | 0.91 |
|  | 5.41 | UQnapus0390 | UQnapus4845 | 0.96 |
|  | 5.41 | UQnapus0390 | UQnapus1858 | 0.91 |
|  | 5.41 | UQnapus0390 | UQnapus1860 | 0.96 |
|  | 5.41 | UQnapus0390 | UQnapus5593 | 0.94 |
|  | 5.41 | UQnapus0390 | UQnapus1863 | 0.88 |
|  | 5.41 | UQnapus0390 | UQnapus5196 | 0.94 |
|  | 5.41 | UQnapus0390 | UQnapus1866 | 0.87 |
|  | 5.41 | UQnapus0390 | UQnapus1868 | 0.94 |
|  | 5.41 | UQnapus0390 | UQnapus1869 | 0.93 |
|  | 5.41 | UQnapus0390 | UQnapus1871 | 0.92 |
|  | 5.41 | UQnapus0390 | UQnapus5379 | 0.81 |
|  | 5.41 | UQnapus0390 | UQnapus1876 | 0.81 |
|  | 5.41 | UQnapus0390 | UQnapus1878 | 0.87 |
|  | 5.41 | UQnapus0390 | UQnapus1884 | 0.79 |
|  | 5.41 | UQnapus0390 | UQnapus1888 | 0.78 |
|  | 5.41 | UQnapus0390 | UQnapus1903 | 0.75 |
|  | 5.41 | UQnapus0390 | UQnapus2241 | 0.83 |
|  | 5.41 | UQnapus0390 | UQnapus5342 | 0.74 |
|  | 5.41 | UQnapus0390 | UQnapus0330 | 0.83 |
|  | 5.41 | UQnapus0390 | UQnapus0332 | 0.83 |
|  | 5.41 | UQnapus0390 | UQnapus0333 | 0.82 |
|  | 5.33 | UQnapus4096 | UQnapus4096 | 1.00 |
|  | 5.33 | UQnapus4096 | UQnapus4807 | 0.83 |
|  | 5.21 | UQnapus5033 | UQnapus5033 | 1.00 |
|  | 5.21 | UQnapus5033 | UQnapus1128 | 0.80 |
|  | 5.21 | UQnapus5033 | UQnapus5495 | 0.80 |
|  | 5.21 | UQnapus5033 | UQnapus1131 | 0.86 |
|  | 5.21 | UQnapus5033 | UQnapus4804 | 0.78 |
|  | 5.21 | UQnapus5033 | UQnapus5497 | 0.78 |
|  | 5.21 | UQnapus5033 | UQnapus1145 | 0.72 |
|  | 5.21 | UQnapus5033 | UQnapus5983 | 0.80 |
|  | 5.21 | UQnapus5033 | UQnapus1122 | 0.82 |
|  | 5.21 | UQnapus5033 | UQnapus1123 | 0.83 |
|  | 5.21 | UQnapus5033 | UQnapus4825 | 0.79 |
|  | 5.09 | UQnapus5554 | UQnapus5554 | 1.00 |
|  | 5.09 | UQnapus5554 | UQnapus1487 | 0.79 |
|  | 5.09 | UQnapus5554 | UQnapus5556 | 0.76 |
|  | 5.09 | UQnapus5554 | UQnapus1490 | 0.83 |
|  | 5.09 | UQnapus5554 | UQnapus2769 | 0.79 |
|  | 5.09 | UQnapus5554 | UQnapus1472 | 0.87 |
|  | 5.09 | UQnapus5554 | UQnapus5552 | 0.87 |
