# Supplementary material for: Genome Wide Analysis of Flowering Time Trait in Multiple Environments via High-Throughput Genotyping Technique in Brassica napus L
Source: PLoS One. 2015 Mar 19;10(3):e0119425. doi: 10.1371/journal.pone.0119425 (PMC4366152; doi:10.1371/journal.pone.0119425)
Supplement: S3 Table — (DOCX) [file pone.0119425.s005.docx]

**S3 Table.** SNPs in the known FT related QTLs

| Linkage group | Marker | Position |
| --- | --- | --- |
| A1 | CB10081 | 0.00 |
| A1 | niab113 | 1.54 |
| A1 | HBr003 | 2.81 |
| A1 | IGF3222b | 3.24 |
| A1 | sN11641 | 3.48 |
| A1 | UQnapus1060 | 5.66 |
| A1 | UQnapus5487 | 6.07 |
| A1 | UQnapus5486 | 6.91 |
| A1 | UQnapus5902 | 6.99 |
| A1 | UQnapus5488 | 9.43 |
| A1 | UQnapus1065 | 9.83 |
| A1 | UQnapus1067 | 10.23 |
| A1 | UQnapus5005 | 10.88 |
| A1 | UQnapus1070 | 12.33 |
| A1 | znS06M34-70 | 12.99 |
| A1 | UQnapus1073 | 13.23 |
| A1 | UQnapus5363 | 14.06 |
| A1 | pW157 | 15.46 |
| A1 | UQnapus1097 | 16.23 |
| A1 | UQnapus1098 | 16.48 |
| A1 | UQnapus1096 | 16.86 |
| A1 | CNU142 | 17.69 |
| A1 | UQnapus4816 | 19.01 |
| A1 | UQnapus1106 | 20.35 |
| A1 | CB10097 | 21.95 |
| A1 | CNU139 | 22.56 |
| A1 | UQnapus1109 | 23.60 |
| A1 | UQnapus5819 | 25.39 |
| A1 | niab096 | 25.89 |
| A1 | HBr006 | 28.45 |
| A1 | JICB0455 | 29.75 |
| A1 | pW145 | 30.08 |
| A1 | UQnapus1022 | 31.80 |
| A1 | UQnapus5494 | 33.00 |
| A1 | UQnapus1122 | 38.24 |
| A1 | UQnapus4825 | 39.29 |
| A1 | UQnapus1123 | 39.54 |
| A1 | UQnapus1128 | 40.37 |
| A1 | UQnapus5983 | 41.32 |
| A1 | UQnapus4804 | 41.90 |
| A1 | UQnapus1129 | 42.26 |
| A1 | UQnapus1131 | 42.51 |
| A1 | UQnapus3176 | 42.68 |
| A1 | UQnapus5497 | 43.28 |
| A1 | UQnapus1137 | 44.25 |
| A1 | UQnapus1138 | 45.15 |
| A1 | UQnapus5183 | 45.17 |
| A1 | UQnapus1145 | 47.58 |
| A1 | UQnapus1147 | 47.93 |
| A1 | UQnapus5365 | 48.57 |
| A1 | UQnapus4827 | 49.08 |
| A1 | UQnapus1151 | 49.71 |
| A1 | UQnapus5268 | 49.78 |
| A1 | IGF9014a | 50.26 |
| A1 | UQnapus1156 | 50.43 |
| A1 | UQnapus5269 | 50.60 |
| A1 | UQnapus4633 | 50.81 |
| A1 | Ol12F11 | 51.02 |
| A1 | UQnapus1161 | 51.47 |
| A1 | IGF0557c | 52.04 |
| A1 | UQnapus1166 | 52.37 |
| A1 | UQnapus1180 | 52.72 |
| A1 | BN35D | 52.92 |
| A1 | BRAS078 | 53.57 |
| A1 | BRAS084 | 53.74 |
| A1 | BRMS-245 | 55.15 |
| A1 | SA63 | 56.47 |
| A1 | BRMS-287 | 56.86 |
| A1 | HR-C004-A1 | 57.13 |
| A1 | UQnapus5501 | 57.41 |
| A1 | UQnapus5381 | 57.78 |
| A1 | UQnapus1234 | 57.97 |
| A1 | UQnapus1933 | 58.31 |
| A1 | UQnapus5739 | 58.68 |
| A1 | UQnapus1938 | 58.87 |
| A1 | UQnapus1946 | 58.97 |
| A1 | UQnapus1952 | 59.06 |
| A1 | UQnapus5604 | 59.09 |
| A1 | UQnapus1948 | 59.10 |
| A1 | UQnapus1949 | 59.12 |
| A1 | UQnapus1940 | 59.13 |
| A1 | UQnapus5331 | 59.42 |
| A1 | UQnapus1197 | 59.42 |
| A1 | UQnapus5500 | 59.43 |
| A1 | UQnapus1186 | 59.80 |
| A1 | UQnapus1570 | 60.07 |
| A1 | UQnapus1240 | 61.55 |
| A1 | ZAAS98b | 62.77 |
| A1 | UQnapus5184 | 63.69 |
| A1 | UQnapus1245 | 63.89 |
| A1 | UQnapus1244 | 63.96 |
| A1 | UQnapus1250 | 66.19 |
| A1 | UQnapus1248 | 66.55 |
| A1 | UQnapus5513 | 67.99 |
| A1 | UQnapus4686 | 69.90 |
| A1 | ZAAS165 | 72.26 |
| A1 | UQnapus0776 | 73.11 |
| A1 | UQnapus1270 | 73.25 |
| A1 | UQnapus5656 | 73.76 |
| A1 | UQnapus1280 | 74.07 |
| A1 | UQnapus1279 | 75.66 |
| A1 | B044E18-1-3 | 77.34 |
| A1 | HBr043 | 79.23 |
| A1 | UQnapus1298 | 84.46 |
| A1 | H007M18-3 | 91.80 |
| A2 | UQnapus0835 | 0.00 |
| A2 | UQnapus3865 | 5.94 |
| A2 | UQnapus3867 | 6.49 |
| A2 | UQnapus4892 | 6.81 |
| A2 | UQnapus3382 | 16.67 |
| A2 | UQnapus3388 | 16.68 |
| A2 | UQnapus4963 | 16.68 |
| A2 | UQnapus3389 | 16.87 |
| A2 | UQnapus5942 | 18.01 |
| A2 | UQnapus3517 | 18.32 |
| A2 | UQnapus3519 | 18.34 |
| A2 | UQnapus3522 | 18.42 |
| A2 | UQnapus3523 | 18.46 |
| A2 | UQnapus4791 | 18.59 |
| A2 | UQnapus1335 | 34.64 |
| A2 | UQnapus0897 | 36.00 |
| A2 | UQnapus1332 | 36.45 |
| A2 | UQnapus1330 | 37.23 |
| A2 | UQnapus1334 | 37.75 |
| A2 | UQnapus5530 | 44.39 |
| A2 | UQnapus5531 | 46.09 |
| A2 | UQnapus1971 | 46.52 |
| A2 | UQnapus1367 | 47.22 |
| A2 | UQnapus4637 | 47.70 |
| A2 | JICB0055 | 47.82 |
| A2 | sR6293a | 48.61 |
| A2 | UQnapus1369 | 48.94 |
| A2 | UQnapus1374 | 50.18 |
| A2 | UQnapus5275 | 50.45 |
| A2 | UQnapus3412 | 50.84 |
| A2 | UQnapus1380 | 51.09 |
| A2 | UQnapus5119 | 51.45 |
| A2 | UQnapus4833 | 51.83 |
| A2 | pW144 | 52.26 |
| A2 | UQnapus1399 | 53.23 |
| A2 | UQnapus3513 | 53.33 |
| A2 | UQnapus0125 | 53.38 |
| A2 | UQnapus1388 | 53.54 |
| A2 | UQnapus0097 | 54.20 |
| A2 | UQnapus1395 | 54.20 |
| A2 | UQnapus1394 | 54.79 |
| A2 | UQnapus1398 | 54.92 |
| A2 | UQnapus5578 | 55.46 |
| A2 | UQnapus4783 | 56.68 |
| A2 | UQnapus1442 | 57.55 |
| A2 | UQnapus4987 | 58.45 |
| A2 | UQnapus1451 | 58.82 |
| A2 | UQnapus1450 | 59.17 |
| A2 | UQnapus5166 | 60.29 |
| A2 | UQnapus1447 | 61.01 |
| A2 | UQnapus1417 | 63.55 |
| A2 | UQnapus5852 | 64.27 |
| A2 | UQnapus1432 | 65.20 |
| A2 | UQnapus1424 | 66.07 |
| A2 | UQnapus4885 | 66.31 |
| A2 | UQnapus1440 | 67.71 |
| A2 | UQnapus5545 | 68.52 |
| A2 | UQnapus1439 | 68.69 |
| A2 | UQnapus0309 | 68.83 |
| A2 | UQnapus2136 | 69.54 |
| A2 | UQnapus2135 | 70.15 |
| A2 | UQnapus4836 | 70.54 |
| A2 | UQnapus2268 | 71.08 |
| A2 | UQnapus5209 | 71.08 |
| A2 | UQnapus5627 | 71.24 |
| A2 | UQnapus1454 | 71.60 |
| A2 | UQnapus2769 | 80.05 |
| A2 | UQnapus1472 | 80.67 |
| A2 | UQnapus5552 | 80.80 |
| A2 | UQnapus5554 | 83.73 |
| A2 | UQnapus5556 | 86.40 |
| A2 | UQnapus1487 | 86.62 |
| A2 | UQnapus1490 | 87.01 |
| A2 | UQnapus5557 | 89.33 |
| A2 | UQnapus1492 | 90.88 |
| A2 | UQnapus1494 | 91.44 |
| A2 | UQnapus1497 | 92.52 |
| A2 | UQnapus1498 | 93.28 |
| A2 | UQnapus1499 | 93.58 |
| A2 | UQnapus1501 | 94.35 |
| A2 | UQnapus1503 | 95.71 |
| A2 | UQnapus1090 | 97.45 |
| A2 | UQnapus1510 | 97.46 |
| A2 | UQnapus1508 | 97.80 |
| A2 | UQnapus1509 | 97.83 |
| A2 | UQnapus4876 | 97.86 |
| A2 | UQnapus0055 | 97.86 |
| A2 | UQnapus1089 | 98.17 |
| A2 | UQnapus0094 | 98.77 |
| A2 | UQnapus5489 | 98.85 |
| A2 | UQnapus1088 | 98.95 |
| A2 | UQnapus0664 | 99.43 |
| A2 | BRMS-228 | 102.13 |
| A2 | UQnapus0558 | 103.44 |
| A2 | UQnapus5180 | 106.98 |
| A2 | UQnapus1081 | 107.35 |
| A2 | UQnapus1513 | 110.15 |
| A2 | CB10355 | 110.90 |
| A3 | BRMS-008 | 0.00 |
| A3 | niab115 | 6.09 |
| A3 | niab116 | 9.73 |
| A3 | B008I08-4 | 15.01 |
| A3 | znS13M26-360 | 16.76 |
| A3 | H003M07-4 | 18.83 |
| A3 | pw189 | 20.19 |
| A3 | bac-3 | 26.81 |
| A3 | HBr061 | 31.24 |
| A3 | B054N05-2 | 32.19 |
| A3 | B071P24-1 | 33.00 |
| A3 | bac-8 | 34.02 |
| A3 | RA2E11 | 34.78 |
| A3 | BRAS029 | 35.02 |
| A3 | HR-Tp2-105 | 36.34 |
| A3 | CB10271 | 36.50 |
| A3 | MR123 | 36.60 |
| A3 | BRMS-303 | 36.69 |
| A3 | HBr073 | 37.22 |
| A3 | IGF2544b | 38.21 |
| A3 | IGF2134f2 | 38.39 |
| A3 | HBr082 | 38.74 |
| A3 | CNU250 | 40.65 |
| A3 | IGF5385a | 41.28 |
| A3 | CNU253 | 41.94 |
| A3 | BRMS-043 | 42.50 |
| A3 | HBr044 | 42.86 |
| A3 | HS-Au14 | 43.46 |
| A3 | HR-Tp4-160 | 43.87 |
| A3 | CB10427 | 44.55 |
| A3 | CNU384 | 44.82 |
| A3 | CNU288 | 45.95 |
| A3 | B068E07-2 | 49.02 |
| A3 | H010E23-2 | 50.08 |
| A3 | H007D13-3-2 | 55.39 |
| A3 | SA29 | 56.49 |
| A3 | HBr049 | 56.71 |
| A3 | HBr050 | 57.19 |
| A3 | H129I15-4-1 | 57.60 |
| A3 | HBr064 | 58.01 |
| A3 | CNU098 | 58.92 |
| A3 | KBrB001H24-13 | 59.06 |
| A3 | B037C07-C3-3 | 60.48 |
| A3 | B044E18-1-2 | 64.08 |
| A3 | Ol11G11a | 65.11 |
| A3 | H034E17-1 | 66.24 |
| A3 | CNU270 | 73.27 |
| A3 | HR-S2-130 | 74.48 |
| A3 | CNU321 | 75.65 |
| A3 | UQnapus0276 | 75.68 |
| A3 | UQnapus4650 | 76.08 |
| A3 | UQnapus1536 | 76.47 |
| A3 | CNU002 | 76.67 |
| A3 | UQnapus1533 | 76.81 |
| A3 | UQnapus1534 | 76.96 |
| A3 | UQnapus1537 | 77.34 |
| A3 | UQnapus1538 | 78.79 |
| A3 | UQnapus1546 | 78.98 |
| A3 | UQnapus1545 | 79.07 |
| A3 | UQnapus1549 | 79.57 |
| A3 | UQnapus5278 | 79.75 |
| A3 | UQnapus1547 | 79.95 |
| A3 | UQnapus1548 | 80.31 |
| A3 | UQnapus1553 | 81.47 |
| A3 | B072K21-4 | 82.73 |
| A3 | CNU215 | 83.10 |
| A3 | UQnapus1554 | 83.40 |
| A3 | HR-Tp4-165 | 83.89 |
| A3 | UQnapus0021 | 85.09 |
| A3 | UQnapus1563 | 85.73 |
| A3 | UQnapus1566 | 85.88 |
| A3 | HBr081 | 86.24 |
| A3 | UQnapus1564 | 86.36 |
| A3 | UQnapus1571 | 86.47 |
| A3 | pW188b | 87.87 |
| A3 | UQnapus1572 | 88.39 |
| A3 | UQnapus5121 | 88.39 |
| A3 | UQnapus1574 | 88.55 |
| A3 | UQnapus5563 | 88.74 |
| A3 | UQnapus4838 | 88.85 |
| A3 | UQnapus5097 | 88.90 |
| A3 | UQnapus5564 | 89.31 |
| A3 | UQnapus1580 | 89.38 |
| A3 | UQnapus1578 | 89.58 |
| A3 | UQnapus1575 | 89.71 |
| A3 | UQnapus1433 | 90.69 |
| A3 | UQnapus5542 | 90.69 |
| A3 | CNU370 | 91.37 |
| A3 | UQnapus1584 | 91.54 |
| A3 | UQnapus4840 | 93.28 |
| A3 | UQnapus0038 | 94.65 |
| A3 | UQnapus4642 | 94.84 |
| A3 | CNU371 | 95.08 |
| A3 | UQnapus1589 | 95.17 |
| A3 | UQnapus5911 | 95.62 |
| A3 | UQnapus0037 | 96.05 |
| A3 | UQnapus2841 | 96.30 |
| A3 | UQnapus2843 | 96.73 |
| A3 | CNU316 | 98.09 |
| A3 | CNU306 | 98.36 |
| A3 | HS-Au39b | 99.76 |
| A3 | pW105 | 102.98 |
| A3 | B085J21-2 | 104.78 |
| A3 | UQnapus1600 | 105.64 |
| A3 | H069E01-1 | 106.86 |
| A3 | UQnapus1601 | 107.33 |
| A3 | B080F13-2 | 108.11 |
| A3 | HBr069 | 109.02 |
| A3 | HBr033 | 111.12 |
| A3 | UQnapus1609 | 111.37 |
| A3 | UQnapus1610 | 111.60 |
| A3 | HBr046 | 111.85 |
| A3 | UQnapus1611 | 112.40 |
| A3 | H035D15-1 | 113.03 |
| A3 | UQnapus1615 | 116.12 |
| A3 | UQnapus1616 | 116.28 |
| A3 | HBr048 | 119.55 |
| A3 | UQnapus1623 | 120.93 |
| A3 | UQnapus1627 | 125.59 |
| A3 | HR-C009-A3 | 126.48 |
| A3 | UQnapus0237 | 127.80 |
| A3 | UQnapus0234 | 128.93 |
| A3 | UQnapus1634 | 129.77 |
| A3 | UQnapus1642 | 132.19 |
| A3 | UQnapus1643 | 132.97 |
| A3 | UQnapus1644 | 133.39 |
| A3 | UQnapus1647 | 137.20 |
| A3 | UQnapus1651 | 137.45 |
| A3 | UQnapus1653 | 138.47 |
| A3 | UQnapus1652 | 138.62 |
| A3 | UQnapus1665 | 139.89 |
| A3 | UQnapus1664 | 140.33 |
| A3 | UQnapus1667 | 140.62 |
| A3 | UQnapus1672 | 142.89 |
| A3 | UQnapus0238 | 144.84 |
| A3 | UQnapus1676 | 146.09 |
| A3 | UQnapus1681 | 146.80 |
| A3 | UQnapus1680 | 147.01 |
| A3 | UQnapus1677 | 147.13 |
| A3 | UQnapus0057 | 149.71 |
| A3 | UQnapus1693 | 151.17 |
| A3 | UQnapus0092 | 157.43 |
| A3 | UQnapus1704 | 157.50 |
| A3 | UQnapus1702 | 157.51 |
| A3 | UQnapus1700 | 157.54 |
| A3 | UQnapus0098 | 157.83 |
| A3 | UQnapus1705 | 157.90 |
| A3 | UQnapus1710 | 159.86 |
| A3 | UQnapus1709 | 160.09 |
| A3 | UQnapus1714 | 161.32 |
| A3 | UQnapus1718 | 162.36 |
| A3 | UQnapus1722 | 162.74 |
| A3 | UQnapus1725 | 163.11 |
| A3 | UQnapus1726 | 163.13 |
| A3 | UQnapus1738 | 163.93 |
| A3 | UQnapus1730 | 164.23 |
| A3 | UQnapus1729 | 164.77 |
| A3 | UQnapus1737 | 165.40 |
| A3 | UQnapus1744 | 166.85 |
| A3 | UQnapus1749 | 173.22 |
| A4 | UQnapus0801 | 0.00 |
| A4 | UQnapus5284 | 1.84 |
| A4 | UQnapus0938 | 2.54 |
| A4 | UQnapus0695 | 2.55 |
| A4 | UQnapus0939 | 3.53 |
| A4 | UQnapus0774 | 4.12 |
| A4 | UQnapus0670 | 4.30 |
| A4 | UQnapus0661 | 4.52 |
| A4 | UQnapus0716 | 4.52 |
| A4 | UQnapus0449 | 4.52 |
| A4 | UQnapus0607 | 4.55 |
| A4 | UQnapus0308 | 4.69 |
| A4 | UQnapus0683 | 8.13 |
| A4 | UQnapus0929 | 10.71 |
| A4 | UQnapus0843 | 15.81 |
| A4 | UQnapus0927 | 32.04 |
| A4 | IGF3395d | 33.93 |
| A4 | IGF3391a | 34.38 |
| A4 | UQnapus0689 | 36.84 |
| A4 | UQnapus1807 | 38.67 |
| A4 | UQnapus1808 | 39.43 |
| A4 | UQnapus1810 | 40.20 |
| A4 | UQnapus1809 | 40.65 |
| A4 | UQnapus1819 | 44.07 |
| A4 | znS13M26-220 | 44.30 |
| A4 | UQnapus1822 | 46.37 |
| A4 | UQnapus1827 | 46.99 |
| A4 | UQnapus1831 | 47.47 |
| A4 | UQnapus1833 | 48.29 |
| A4 | UQnapus1830 | 48.49 |
| A4 | UQnapus1826 | 48.65 |
| A4 | UQnapus1858 | 49.41 |
| A4 | UQnapus1860 | 50.40 |
| A4 | UQnapus0390 | 50.69 |
| A4 | UQnapus1866 | 51.23 |
| A4 | UQnapus1869 | 52.43 |
| A4 | UQnapus1868 | 52.50 |
| A4 | UQnapus1871 | 52.76 |
| A4 | UQnapus0332 | 54.09 |
| A4 | UQnapus0330 | 54.11 |
| A4 | UQnapus1878 | 54.58 |
| A4 | UQnapus0333 | 54.85 |
| A4 | UQnapus1876 | 55.88 |
| A4 | UQnapus1884 | 56.04 |
| A4 | UQnapus1888 | 56.33 |
| A4 | UQnapus1909 | 59.68 |
| A4 | UQnapus1916 | 60.89 |
| A4 | UQnapus1914 | 61.01 |
| A4 | UQnapus1918 | 61.77 |
| A4 | UQnapus1922 | 62.12 |
| A4 | UQnapus1923 | 62.60 |
| A4 | UQnapus1927 | 64.11 |
| A4 | UQnapus1929 | 65.94 |
| A4 | UQnapus1958 | 69.53 |
| A4 | UQnapus1960 | 70.19 |
| A4 | UQnapus1959 | 70.49 |
| A4 | UQnapus1956 | 71.03 |
| A4 | UQnapus1984 | 72.65 |
| A4 | UQnapus1965 | 73.01 |
| A4 | UQnapus1966 | 73.34 |
| A4 | IGF2021e | 74.11 |
| A4 | UQnapus1970 | 74.79 |
| A4 | UQnapus1972 | 80.11 |
| A4 | BRMS-105 | 82.70 |
| A4 | BRMS-054 | 82.90 |
| A4 | HBr089 | 83.60 |
| A4 | UQnapus1979 | 84.13 |
| A4 | HBr091 | 84.74 |
| A4 | UQnapus1975 | 85.23 |
| A4 | UQnapus1986 | 86.16 |
| A4 | UQnapus1988 | 87.92 |
| A4 | UQnapus1994 | 92.12 |
| A4 | UQnapus1996 | 93.17 |
| A4 | UQnapus1995 | 93.96 |
| A4 | UQnapus1999 | 98.23 |
| A4 | UQnapus2001 | 98.34 |
| A4 | UQnapus2002 | 98.69 |
| A4 | UQnapus2008 | 99.74 |
| A4 | UQnapus2010 | 101.88 |
| A4 | UQnapus2015 | 106.63 |
| A4 | UQnapus2016 | 107.12 |
| A4 | UQnapus1518 | 109.21 |
| A4 | UQnapus0039 | 113.48 |
| A4 | JICB0584 | 115.00 |
| A4 | UQnapus2020 | 123.75 |
| A5 | CB10487 | 0.00 |
| A5 | IGF9007a | 2.51 |
| A5 | JICB0384 | 3.22 |
| A5 | BRMS-007 | 4.59 |
| A5 | UQnapus2023 | 6.79 |
| A5 | UQnapus1511 | 7.24 |
| A5 | UQnapus1515 | 8.22 |
| A5 | UQnapus0321 | 9.18 |
| A5 | UQnapus1516 | 9.43 |
| A5 | UQnapus5371 | 9.63 |
| A5 | UQnapus0286 | 10.58 |
| A5 | UQnapus0306 | 11.11 |
| A5 | UQnapus2025 | 12.61 |
| A5 | UQnapus5615 | 12.85 |
| A5 | UQnapus5090 | 14.76 |
| A5 | UQnapus1476 | 15.89 |
| A5 | UQnapus5288 | 16.51 |
| A5 | UQnapus5091 | 16.61 |
| A5 | UQnapus2028 | 17.53 |
| A5 | UQnapus2029 | 17.76 |
| A5 | UQnapus2030 | 19.00 |
| A5 | UQnapus5092 | 21.64 |
| A5 | IGF3134a | 25.08 |
| A5 | sR9477 | 25.64 |
| A5 | UQnapus5616 | 26.78 |
| A5 | pW247 | 29.34 |
| A5 | HR-Tp4-200 | 30.04 |
| A5 | CNU268 | 33.23 |
| A5 | UQnapus2042 | 35.23 |
| A5 | UQnapus2043 | 35.80 |
| A5 | UQnapus2045 | 37.80 |
| A5 | UQnapus2046 | 43.22 |
| A5 | IGF3165a | 43.63 |
| A5 | UQnapus0492 | 44.33 |
| A5 | UQnapus0179 | 44.88 |
| A5 | UQnapus0177 | 45.24 |
| A5 | UQnapus0139 | 45.38 |
| A5 | UQnapus0181 | 46.23 |
| A5 | UQnapus0184 | 46.83 |
| A5 | UQnapus0183 | 46.93 |
| A5 | UQnapus0188 | 49.12 |
| A5 | UQnapus0189 | 49.65 |
| A5 | UQnapus2118 | 54.85 |
| A5 | UQnapus0868 | 55.58 |
| A5 | HBr101 | 55.76 |
| A5 | UQnapus5742 | 55.89 |
| A5 | UQnapus2125 | 56.16 |
| A5 | UQnapus2068 | 56.28 |
| A5 | UQnapus1163 | 56.40 |
| A5 | UQnapus2115 | 56.66 |
| A5 | UQnapus2088 | 56.68 |
| A5 | UQnapus2114 | 56.73 |
| A5 | UQnapus5621 | 56.73 |
| A5 | UQnapus2072 | 56.87 |
| A5 | UQnapus5622 | 56.87 |
| A5 | UQnapus2070 | 56.88 |
| A5 | UQnapus5170 | 56.88 |
| A5 | UQnapus2074 | 56.89 |
| A5 | UQnapus5247 | 56.90 |
| A5 | UQnapus2084 | 56.93 |
| A5 | UQnapus2087 | 56.94 |
| A5 | UQnapus2081 | 56.95 |
| A5 | UQnapus4849 | 56.96 |
| A5 | UQnapus5012 | 56.98 |
| A5 | UQnapus2111 | 57.21 |
| A5 | UQnapus2108 | 57.30 |
| A5 | UQnapus2057 | 57.57 |
| A5 | HR-C016-A5 | 58.04 |
| A5 | H012G24 | 58.12 |
| A5 | H080L24 | 58.21 |
| A5 | UQnapus2092 | 58.63 |
| A5 | UQnapus0129 | 58.83 |
| A5 | UQnapus2060 | 58.95 |
| A5 | UQnapus2751 | 59.43 |
| A5 | UQnapus4813 | 59.47 |
| A5 | UQnapus2121 | 59.70 |
| A5 | UQnapus2150 | 64.03 |
| A5 | UQnapus2157 | 65.88 |
| A5 | UQnapus2161 | 68.07 |
| A5 | UQnapus2164 | 68.18 |
| A5 | UQnapus2165 | 68.28 |
| A5 | UQnapus2166 | 68.38 |
| A5 | UQnapus5637 | 68.90 |
| A5 | UQnapus2167 | 69.15 |
| A5 | UQnapus2169 | 70.55 |
| A5 | UQnapus5205 | 71.35 |
| A5 | UQnapus2175 | 73.70 |
| A5 | UQnapus2179 | 75.35 |
| A5 | UQnapus2176 | 75.78 |
| A5 | UQnapus0371 | 79.72 |
| A5 | BRAS063 | 80.93 |
| A5 | em06me07-250 | 81.71 |
| A5 | UQnapus5030 | 85.70 |
| A5 | UQnapus2189 | 86.19 |
| A5 | CNU286 | 87.28 |
| A5 | BRMS-061 | 87.56 |
| A5 | niab017 | 89.68 |
| A5 | UQnapus2197 | 93.71 |
| A5 | BRMS-034 | 95.19 |
| A5 | JICB0153 | 96.76 |
| A5 | JICB0094 | 98.03 |
| A5 | UQnapus2200 | 99.78 |
| A5 | UQnapus0305 | 103.98 |
| A5 | UQnapus2207 | 107.17 |
| A5 | sNRD03 | 114.53 |
| A6 | JICB0537 | 0.00 |
| A6 | UQnapus0629 | 4.39 |
| A6 | UQnapus5211 | 4.97 |
| A6 | UQnapus5210 | 6.06 |
| A6 | UQnapus2356 | 6.06 |
| A6 | UQnapus5172 | 14.16 |
| A6 | UQnapus2348 | 14.93 |
| A6 | UQnapus2347 | 18.36 |
| A6 | UQnapus2346 | 20.16 |
| A6 | UQnapus2342 | 24.60 |
| A6 | UQnapus5666 | 25.48 |
| A6 | UQnapus5922 | 26.87 |
| A6 | HBr029 | 27.88 |
| A6 | CNU219 | 28.08 |
| A6 | IGF3239d | 29.87 |
| A6 | UQnapus2312 | 34.33 |
| A6 | UQnapus2336 | 34.75 |
| A6 | UQnapus5877 | 36.32 |
| A6 | UQnapus0451 | 38.60 |
| A6 | UQnapus2310 | 40.06 |
| A6 | UQnapus5131 | 40.99 |
| A6 | UQnapus2308 | 40.99 |
| A6 | UQnapus2307 | 41.52 |
| A6 | UQnapus4990 | 42.52 |
| A6 | UQnapus2305 | 42.56 |
| A6 | UQnapus2301 | 42.97 |
| A6 | UQnapus5294 | 44.36 |
| A6 | UQnapus2297 | 46.06 |
| A6 | UQnapus2296 | 46.34 |
| A6 | JICB2036_550 | 47.29 |
| A6 | JICB2011_176 | 47.93 |
| A6 | UQnapus0281 | 48.08 |
| A6 | UQnapus2328 | 48.42 |
| A6 | UQnapus2327 | 48.55 |
| A6 | UQnapus2323 | 49.06 |
| A6 | UQnapus5457 | 49.06 |
| A6 | UQnapus2318 | 49.14 |
| A6 | UQnapus2941 | 49.58 |
| A6 | UQnapus2317 | 49.91 |
| A6 | UQnapus5253 | 49.95 |
| A6 | HR-C006-A6 | 50.15 |
| A6 | UQnapus0255 | 50.41 |
| A6 | UQnapus3796 | 50.64 |
| A6 | UQnapus2942 | 50.79 |
| A6 | UQnapus0279 | 51.08 |
| A6 | UQnapus2289 | 51.87 |
| A6 | UQnapus2292 | 52.04 |
| A6 | pW127b | 52.33 |
| A6 | UQnapus5344 | 52.42 |
| A6 | HBr013 | 52.80 |
| A6 | UQnapus2287 | 53.13 |
| A6 | UQnapus5660 | 53.34 |
| A6 | UQnapus2286 | 53.48 |
| A6 | UQnapus2285 | 53.73 |
| A6 | UQnapus5658 | 56.43 |
| A6 | UQnapus2281 | 57.08 |
| A6 | UQnapus5343 | 57.11 |
| A6 | UQnapus2279 | 57.93 |
| A6 | UQnapus2277 | 59.73 |
| A6 | UQnapus2244 | 63.53 |
| A6 | UQnapus5255 | 63.68 |
| A6 | UQnapus2245 | 64.82 |
| A6 | pW123aE | 65.72 |
| A6 | KBrB086M23-6_580 | 66.04 |
| A6 | BRMS-030 | 66.48 |
| A6 | pW123bH | 66.81 |
| A6 | UQnapus2250 | 68.06 |
| A6 | Ol10D01 | 68.77 |
| A6 | UQnapus2254 | 71.19 |
| A6 | UQnapus5040 | 71.19 |
| A6 | S004C14-2 | 73.31 |
| A6 | UQnapus5654 | 73.55 |
| A6 | UQnapus2261 | 73.55 |
| A6 | UQnapus2262 | 74.13 |
| A6 | UQnapus5393 | 74.14 |
| A6 | IGF1139e | 75.18 |
| A6 | UQnapus2264 | 75.59 |
| A6 | UQnapus5394 | 75.59 |
| A6 | IGF5298c | 76.97 |
| A6 | UQnapus5252 | 77.10 |
| A6 | pW199 | 77.64 |
| A6 | UQnapus2267 | 78.39 |
| A6 | UQnapus0130 | 78.39 |
| A6 | UQnapus0325 | 79.18 |
| A6 | UQnapus5651 | 79.49 |
| A6 | UQnapus5649 | 80.59 |
| A6 | UQnapus5650 | 80.71 |
| A6 | IGF1027z | 81.49 |
| A6 | IGF1027b | 81.81 |
| A6 | UQnapus2234 | 83.21 |
| A6 | UQnapus2233 | 84.76 |
| A6 | UQnapus5391 | 84.76 |
| A6 | UQnapus2231 | 86.32 |
| A6 | HS-b09-1 | 87.13 |
| A6 | HS-q11-1 | 88.07 |
| A6 | UQnapus2229 | 88.57 |
| A6 | CB10143 | 90.35 |
| A6 | BRMS-027 | 92.16 |
| A6 | BRMS-101 | 92.76 |
| A6 | UQnapus2225 | 93.88 |
| A6 | UQnapus5645 | 96.20 |
| A6 | UQnapus2222 | 96.21 |
| A6 | UQnapus2221 | 96.96 |
| A6 | UQnapus2218 | 97.36 |
| A6 | B065N20 | 97.72 |
| A6 | B030D08 | 98.14 |
| A6 | HS-WA7 | 98.80 |
| A6 | UQnapus2219 | 99.35 |
| A6 | UQnapus5093 | 99.35 |
| A6 | H071I03 | 100.13 |
| A6 | ZAAS92b | 101.05 |
| A6 | pW217 | 102.01 |
| A6 | UQnapus2211 | 102.97 |
| A6 | UQnapus5207 | 104.92 |
| A6 | HR-Tp3-320 | 108.93 |
| A6 | Ol11F12a | 117.04 |
| A7 | JICB0454 | 0.00 |
| A7 | UQnapus5003 | 4.86 |
| A7 | UQnapus3040 | 5.17 |
| A7 | UQnapus3037 | 5.47 |
| A7 | UQnapus5056 | 5.52 |
| A7 | UQnapus5421 | 5.92 |
| A7 | UQnapus3032 | 6.28 |
| A7 | UQnapus2378 | 6.99 |
| A7 | UQnapus1898 | 7.33 |
| A7 | UQnapus2376 | 7.60 |
| A7 | UQnapus5285 | 7.70 |
| A7 | UQnapus5669 | 7.90 |
| A7 | UQnapus2377 | 7.94 |
| A7 | pX116a | 8.80 |
| A7 | UQnapus2394 | 9.14 |
| A7 | UQnapus2396 | 9.34 |
| A7 | HR-C011-A7b | 9.60 |
| A7 | UQnapus2418 | 10.20 |
| A7 | UQnapus2413 | 10.53 |
| A7 | UQnapus2400 | 10.71 |
| A7 | UQnapus2401 | 11.07 |
| A7 | UQnapus2403 | 11.26 |
| A7 | UQnapus2422 | 11.78 |
| A7 | UQnapus2423 | 12.27 |
| A7 | em19me20-700 | 14.47 |
| A7 | UQnapus2431 | 15.00 |
| A7 | IGF1092c | 16.05 |
| A7 | UQnapus2441 | 17.49 |
| A7 | UQnapus2449 | 17.96 |
| A7 | BRAS023 | 18.17 |
| A7 | BRMS-018 | 18.28 |
| A7 | UQnapus2451 | 19.78 |
| A7 | UQnapus2453 | 20.46 |
| A7 | UQnapus4660 | 20.61 |
| A7 | UQnapus2452 | 20.80 |
| A7 | UQnapus4661 | 25.50 |
| A7 | UQnapus2467 | 26.55 |
| A7 | UQnapus2462 | 26.76 |
| A7 | UQnapus5094 | 26.92 |
| A7 | IGF1178b | 28.20 |
| A7 | UQnapus2466 | 28.57 |
| A7 | HR-Sp3-255 | 31.39 |
| A7 | UQnapus1307 | 31.71 |
| A7 | UQnapus1323 | 33.53 |
| A7 | UQnapus1309 | 34.76 |
| A7 | UQnapus1311 | 35.04 |
| A7 | UQnapus1312 | 35.10 |
| A7 | UQnapus5368 | 35.10 |
| A7 | UQnapus1317 | 35.40 |
| A7 | UQnapus1319 | 35.55 |
| A7 | UQnapus1316 | 35.78 |
| A7 | UQnapus4832 | 35.81 |
| A7 | UQnapus1318 | 36.09 |
| A7 | UQnapus5524 | 36.35 |
| A7 | UQnapus5525 | 38.19 |
| A7 | UQnapus2476 | 42.39 |
| A7 | UQnapus5095 | 42.66 |
| A7 | KBrB084H08-24 | 44.40 |
| A7 | UQnapus2540 | 53.17 |
| A7 | UQnapus2522 | 60.41 |
| A7 | RPSaA | 61.06 |
| A7 | UQnapus2534 | 61.94 |
| A7 | znS06M34-50 | 62.63 |
| A7 | UQnapus4952 | 63.65 |
| A7 | UQnapus4951 | 63.78 |
| A7 | UQnapus0073 | 63.86 |
| A7 | UQnapus0174 | 64.02 |
| A7 | UQnapus0089 | 64.78 |
| A7 | UQnapus0175 | 64.82 |
| A7 | UQnapus4782 | 64.95 |
| A7 | UQnapus0171 | 64.95 |
| A7 | UQnapus0015 | 64.96 |
| A7 | UQnapus0068 | 64.97 |
| A7 | UQnapus2539 | 64.99 |
| A7 | UQnapus0016 | 64.99 |
| A7 | UQnapus0033 | 65.02 |
| A7 | UQnapus4781 | 65.04 |
| A7 | UQnapus4780 | 65.06 |
| A7 | UQnapus0113 | 65.17 |
| A7 | UQnapus0024 | 65.20 |
| A7 | UQnapus2538 | 65.26 |
| A7 | UQnapus0017 | 65.61 |
| A7 | UQnapus2553 | 67.70 |
| A7 | UQnapus5400 | 68.27 |
| A7 | UQnapus5401 | 68.53 |
| A7 | UQnapus2551 | 68.82 |
| A7 | UQnapus0018 | 70.69 |
| A7 | UQnapus0047 | 71.09 |
| A7 | UQnapus4955 | 71.24 |
| A7 | IGF3309b | 74.20 |
| A7 | UQnapus2572 | 79.66 |
| A7 | CNU331 | 81.83 |
| A7 | HG-WRI1-A7 | 82.43 |
| A7 | CNU168 | 85.48 |
| A7 | JICB0553 | 86.65 |
| A7 | UQnapus0134 | 87.78 |
| A7 | UQnapus2592 | 87.90 |
| A7 | UQnapus2585 | 89.41 |
| A7 | UQnapus2595 | 90.50 |
| A7 | UQnapus2591 | 90.70 |
| A7 | JICB0274 | 91.23 |
| A7 | HG-FT-A7a | 91.44 |
| A7 | HBr009 | 91.67 |
| A7 | IGF1226f | 92.27 |
| A7 | JICB0011 | 92.55 |
| A7 | IGF1226l | 92.77 |
| A7 | JICB0416 | 94.01 |
| A7 | CNU053b | 96.35 |
| A7 | IGF1226a | 101.91 |
| A7 | CB10211 | 104.18 |
| A8 | HR-Tp3-390 | 0.00 |
| A8 | HBr026 | 3.31 |
| A8 | UQnapus2766 | 7.10 |
| A8 | UQnapus5715 | 7.10 |
| A8 | JICB0018 | 8.36 |
| A8 | UQnapus4994 | 9.99 |
| A8 | UQnapus0819 | 12.97 |
| A8 | UQnapus5259 | 13.97 |
| A8 | HR-C002-A8 | 14.78 |
| A8 | HR-S4-295 | 16.11 |
| A8 | CNU090 | 17.07 |
| A8 | HBr074 | 18.62 |
| A8 | sR7178 | 18.81 |
| A8 | UQnapus5499 | 19.45 |
| A8 | HBr016 | 19.70 |
| A8 | UQnapus2701 | 20.06 |
| A8 | UQnapus2692 | 20.40 |
| A8 | HBr015 | 20.66 |
| A8 | UQnapus2695 | 20.88 |
| A8 | sS1702 | 21.25 |
| A8 | UQnapus2699 | 21.58 |
| A8 | HG-FAE1-A8 | 22.47 |
| A8 | RA2E12 | 22.97 |
| A8 | UQnapus2093 | 25.37 |
| A8 | UQnapus2094 | 25.80 |
| A8 | UQnapus2302 | 25.82 |
| A8 | UQnapus1438 | 25.90 |
| A8 | UQnapus1436 | 26.04 |
| A8 | UQnapus2688 | 26.29 |
| A8 | UQnapus2680 | 29.35 |
| A8 | UQnapus5699 | 30.72 |
| A8 | UQnapus2677 | 30.72 |
| A8 | UQnapus2673 | 31.60 |
| A8 | UQnapus2672 | 33.99 |
| A8 | UQnapus2668 | 35.99 |
| A8 | UQnapus4993 | 36.16 |
| A8 | UQnapus5138 | 37.66 |
| A8 | UQnapus2666 | 38.65 |
| A8 | UQnapus4861 | 39.04 |
| A8 | UQnapus2663 | 41.78 |
| A8 | UQnapus2662 | 42.10 |
| A8 | UQnapus2650 | 46.33 |
| A8 | UQnapus5404 | 46.33 |
| A8 | UQnapus2654 | 47.02 |
| A8 | UQnapus2653 | 47.29 |
| A8 | HBr010 | 48.38 |
| A8 | UQnapus2649 | 49.06 |
| A8 | UQnapus2643 | 50.58 |
| A8 | HAU327 | 52.76 |
| A8 | UQnapus2634 | 57.43 |
| A8 | HBr017 | 58.01 |
| A8 | HAU345 | 58.01 |
| A8 | UQnapus5696 | 58.91 |
| A8 | HAU342 | 59.86 |
| A8 | B034P14-1-1 | 59.99 |
| A8 | HAU348 | 61.53 |
| A8 | UQnapus2631 | 62.35 |
| A8 | HAU346 | 63.69 |
| A8 | HG4-SC9TN | 74.40 |
| A8 | UQnapus5216 | 77.21 |
| A8 | HG4-SCD2 | 81.81 |
| A9 | UQnapus2776 | 0.00 |
| A9 | UQnapus2779 | 4.23 |
| A9 | UQnapus2787 | 9.86 |
| A9 | UQnapus2791 | 11.10 |
| A9 | UQnapus5099 | 11.12 |
| A9 | UQnapus2792 | 12.34 |
| A9 | UQnapus2795 | 18.70 |
| A9 | UQnapus5467 | 20.76 |
| A9 | UQnapus2796 | 20.96 |
| A9 | UQnapus2797 | 21.37 |
| A9 | UQnapus2798 | 24.61 |
| A9 | UQnapus4491 | 28.98 |
| A9 | UQnapus0277 | 35.04 |
| A9 | UQnapus5178 | 37.18 |
| A9 | UQnapus3035 | 37.83 |
| A9 | UQnapus4521 | 39.12 |
| A9 | UQnapus0137 | 40.58 |
| A9 | UQnapus5306 | 43.31 |
| A9 | UQnapus5468 | 43.63 |
| A9 | UQnapus2809 | 43.71 |
| A9 | UQnapus2810 | 44.46 |
| A9 | UQnapus5414 | 45.03 |
| A9 | UQnapus4867 | 46.98 |
| A9 | UQnapus2822 | 47.44 |
| A9 | UQnapus2823 | 47.57 |
| A9 | ZAAS246b | 48.11 |
| A9 | KBrB073D09-12 | 49.41 |
| A9 | UQnapus2825 | 49.70 |
| A9 | UQnapus5141 | 49.70 |
| A9 | UQnapus2826 | 49.77 |
| A9 | UQnapus2829 | 49.81 |
| A9 | UQnapus5415 | 50.52 |
| A9 | UQnapus4977 | 50.82 |
| A9 | UQnapus2830 | 51.08 |
| A9 | UQnapus2828 | 51.79 |
| A9 | UQnapus4429 | 52.33 |
| A9 | UQnapus5727 | 52.93 |
| A9 | UQnapus2340 | 53.14 |
| A9 | UQnapus2831 | 53.58 |
| A9 | UQnapus2836 | 55.75 |
| A9 | UQnapus0191 | 55.93 |
| A9 | UQnapus0192 | 55.95 |
| A9 | UQnapus0196 | 56.06 |
| A9 | UQnapus4869 | 56.46 |
| A9 | UQnapus4996 | 56.46 |
| A9 | UQnapus5905 | 56.59 |
| A9 | UQnapus2845 | 56.82 |
| A9 | UQnapus5730 | 56.98 |
| A9 | UQnapus0327 | 57.47 |
| A9 | UQnapus2850 | 59.75 |
| A9 | UQnapus2851 | 60.53 |
| A9 | UQnapus5469 | 65.98 |
| A9 | HBr036 | 66.29 |
| A9 | UQnapus4674 | 66.89 |
| A9 | UQnapus2883 | 66.89 |
| A9 | UQnapus2884 | 66.91 |
| A9 | UQnapus0250 | 67.21 |
| A9 | UQnapus2873 | 67.38 |
| A9 | UQnapus2479 | 67.52 |
| A9 | H032N11-2 | 67.70 |
| A9 | UQnapus4874 | 68.09 |
| A9 | UQnapus2877 | 68.32 |
| A9 | UQnapus5736 | 68.54 |
| A9 | UQnapus5930 | 68.55 |
| A9 | UQnapus0337 | 68.56 |
| A9 | UQnapus2880 | 68.57 |
| A9 | UQnapus2876 | 68.58 |
| A9 | UQnapus2480 | 68.60 |
| A9 | UQnapus2874 | 68.60 |
| A9 | HR-C013-A9b | 68.68 |
| A9 | UQnapus1475 | 68.70 |
| A9 | UQnapus5553 | 68.71 |
| A9 | UQnapus2881 | 68.73 |
| A9 | UQnapus2869 | 68.74 |
| A9 | UQnapus2909 | 68.80 |
| A9 | UQnapus2481 | 68.83 |
| A9 | UQnapus2867 | 69.06 |
| A9 | UQnapus0304 | 69.26 |
| A9 | UQnapus2906 | 69.31 |
| A9 | UQnapus2855 | 69.37 |
| A9 | H143H14-2 | 69.68 |
| A9 | UQnapus2870 | 70.19 |
| A9 | HBr072 | 71.62 |
| A9 | em18me23-380 | 72.09 |
| A9 | Na10A08 | 72.23 |
| A9 | CB10022 | 73.40 |
| A9 | UQnapus2905 | 77.59 |
| A9 | UQnapus5737 | 77.83 |
| A9 | UQnapus2919 | 81.12 |
| A9 | UQnapus5873 | 82.33 |
| A9 | UQnapus2920 | 82.34 |
| A9 | HBr034 | 84.00 |
| A9 | UQnapus5149 | 84.50 |
| A9 | UQnapus2922 | 85.63 |
| A9 | B039K15-5-2 | 86.09 |
| A9 | HBr063 | 87.07 |
| A9 | UQnapus0274 | 87.37 |
| A9 | UQnapus5965 | 92.06 |
| A9 | UQnapus5143 | 92.16 |
| A9 | UQnapus2952 | 92.48 |
| A9 | HBr053 | 92.55 |
| A9 | UQnapus5176 | 94.82 |
| A9 | UQnapus5309 | 95.09 |
| A9 | IGF2029a | 95.55 |
| A9 | UQnapus2954 | 98.36 |
| A9 | UQnapus2953 | 98.62 |
| A9 | UQnapus2955 | 104.11 |
| A9 | UQnapus5850 | 109.83 |
| A9 | UQnapus1185 | 114.66 |
| A9 | UQnapus1184 | 116.65 |
| A9 | UQnapus5504 | 116.68 |
| A9 | UQnapus4829 | 118.42 |
| A10 | UQnapus3242 | 0.00 |
| A10 | IGF5108e | 5.77 |
| A10 | UQnapus1805 | 8.64 |
| A10 | UQnapus1806 | 8.77 |
| A10 | UQnapus5123 | 8.77 |
| A10 | UQnapus1804 | 8.87 |
| A10 | UQnapus2968 | 9.08 |
| A10 | UQnapus2970 | 11.43 |
| A10 | BRMS-019 | 11.95 |
| A10 | UQnapus0874 | 16.14 |
| A10 | UQnapus2985 | 18.12 |
| A10 | UQnapus5471 | 19.58 |
| A10 | UQnapus2983 | 20.96 |
| A10 | UQnapus5144 | 21.53 |
| A10 | UQnapus2982 | 22.76 |
| A10 | UQnapus5751 | 23.98 |
| A10 | UQnapus2980 | 23.98 |
| A10 | BRMS-062 | 25.93 |
| A10 | BRMS-244 | 28.20 |
| A10 | UQnapus0224 | 29.51 |
| A10 | JICB0272 | 30.08 |
| A10 | UQnapus2977 | 34.24 |
| A10 | UQnapus5750 | 34.24 |
| A10 | UQnapus2973 | 35.16 |
| A10 | UQnapus5749 | 35.16 |
| A10 | UQnapus2989 | 37.35 |
| A10 | UQnapus2987 | 37.43 |
| A10 | niab009 | 38.53 |
| A10 | HG-FLC-A10 | 39.25 |
| A10 | sN8474 | 39.82 |
| A10 | UQnapus3007 | 42.13 |
| A10 | UQnapus3003 | 42.35 |
| A10 | UQnapus5758 | 42.37 |
| A10 | UQnapus3004 | 42.41 |
| A10 | sN8502 | 45.77 |
| A10 | pw155b | 49.87 |
| A10 | UQnapus5009 | 50.89 |
| A10 | UQnapus1779 | 51.41 |
| A10 | UQnapus1778 | 52.66 |
| A10 | HG4-BSR7 | 53.12 |
| A10 | HG4-BSR4 | 53.35 |
| A10 | HG4-BSR5 | 53.89 |
| A10 | UQnapus5583 | 54.72 |
| A10 | niab123 | 56.08 |
| A10 | UQnapus5336 | 56.39 |
| A10 | UQnapus1761 | 57.51 |
| A10 | UQnapus5844 | 57.86 |
| A10 | UQnapus4618 | 58.00 |
| A10 | UQnapus1763 | 58.24 |
| A10 | UQnapus1764 | 58.33 |
| A10 | UQnapus1756 | 59.73 |
| A10 | UQnapus5375 | 60.06 |
| A10 | UQnapus1757 | 60.32 |
| A10 | UQnapus5335 | 62.22 |
| A10 | Ss2066 | 62.83 |
| A10 | UQnapus1750 | 63.29 |
| A10 | HS-b14-1 | 64.27 |
| A10 | Na10D07a | 65.19 |
| A10 | UQnapus5262 | 65.33 |
| A10 | RA2E03 | 66.36 |
| A10 | UQnapus3057 | 66.70 |
| A10 | UQnapus3056 | 67.40 |
| A10 | UQnapus3059 | 67.54 |
| A10 | UQnapus5766 | 68.53 |
| A10 | UQnapus3062 | 68.66 |
| A10 | UQnapus5704 | 68.83 |
| A10 | UQnapus3069 | 69.38 |
| A10 | UQnapus1597 | 69.63 |
| A10 | UQnapus3081 | 69.89 |
| A10 | UQnapus3079 | 70.18 |
| A10 | UQnapus5314 | 70.36 |
| A10 | UQnapus3082 | 70.36 |
| A10 | UQnapus4972 | 70.58 |
| A10 | UQnapus4871 | 70.69 |
| A10 | em15me28-200 | 70.83 |
| A10 | UQnapus5770 | 71.19 |
| A10 | UQnapus3086 | 71.19 |
| A10 | UQnapus5767 | 71.38 |
| A10 | UQnapus3073 | 71.38 |
| A10 | pW240 | 71.82 |
| A10 | UQnapus5771 | 72.20 |
| A10 | UQnapus3096 | 72.20 |
| A10 | UQnapus3095 | 72.33 |
| A10 | UQnapus3087 | 72.66 |
| A10 | UQnapus3089 | 73.49 |
| A10 | IGF1073a | 73.84 |
| A10 | HR-C003-A10 | 74.08 |
| A10 | UQnapus2697 | 74.26 |
| A10 | Na12D11 | 74.51 |
| A10 | niab015 | 76.22 |
| A10 | UQnapus4679 | 77.77 |
| A10 | UQnapus3125 | 77.79 |
| A10 | UQnapus5779 | 77.80 |
| A10 | UQnapus3124 | 77.98 |
| A10 | UQnapus5263 | 78.69 |
| A10 | UQnapus3123 | 78.99 |
| A10 | niab034 | 81.33 |
| A10 | UQnapus3117 | 81.83 |
| A10 | pW117 | 82.64 |
| A10 | UQnapus4997 | 84.15 |
| A10 | UQnapus4872 | 85.38 |
| C1 | UQnapus4537 | 0.00 |
| C1 | UQnapus3837 | 5.27 |
| C1 | UQnapus3833 | 8.00 |
| C1 | UQnapus4890 | 9.59 |
| C1 | UQnapus3186 | 12.20 |
| C1 | UQnapus0518 | 16.87 |
| C1 | UQnapus3169 | 28.23 |
| C1 | IGF2041E | 29.06 |
| C1 | UQnapus3166 | 32.36 |
| C1 | HR-Sp2-190 | 34.95 |
| C1 | HR-S10-135 | 35.87 |
| C1 | CB10208 | 36.51 |
| C1 | BRMS-056 | 40.07 |
| C1 | IGF2026c | 41.07 |
| C1 | UQnapus5325 | 41.52 |
| C1 | CB10369 | 43.50 |
| C1 | UQnapus3177 | 43.95 |
| C1 | UQnapus0002 | 45.43 |
| C1 | UQnapus3181 | 50.42 |
| C1 | UQnapus5821 | 51.81 |
| C1 | UQnapus0457 | 52.57 |
| C1 | UQnapus3341 | 52.94 |
| C1 | UQnapus5109 | 53.07 |
| C1 | UQnapus3212 | 53.28 |
| C1 | UQnapus3211 | 53.31 |
| C1 | UQnapus3206 | 53.42 |
| C1 | UQnapus3210 | 53.43 |
| C1 | UQnapus3209 | 53.54 |
| C1 | UQnapus3207 | 53.91 |
| C1 | UQnapus5826 | 53.95 |
| C1 | UQnapus5825 | 54.08 |
| C1 | UQnapus5025 | 54.34 |
| C1 | UQnapus3203 | 54.35 |
| C1 | UQnapus4878 | 54.37 |
| C1 | UQnapus5433 | 55.53 |
| C1 | UQnapus3198 | 55.59 |
| C1 | UQnapus3253 | 60.22 |
| C1 | UQnapus0969 | 60.36 |
| C1 | UQnapus3760 | 60.80 |
| C1 | UQnapus0792 | 61.30 |
| C1 | UQnapus3407 | 61.40 |
| C1 | UQnapus0920 | 62.47 |
| C1 | UQnapus4934 | 63.44 |
| C1 | UQnapus3320 | 63.51 |
| C1 | UQnapus3313 | 64.11 |
| C1 | UQnapus3335 | 64.19 |
| C1 | UQnapus3330 | 64.24 |
| C1 | UQnapus0665 | 64.25 |
| C1 | UQnapus3324 | 64.26 |
| C1 | UQnapus5841 | 64.41 |
| C1 | UQnapus5437 | 64.47 |
| C1 | UQnapus3319 | 64.48 |
| C1 | UQnapus3312 | 64.55 |
| C1 | UQnapus0701 | 64.95 |
| C1 | UQnapus3325 | 65.03 |
| C1 | UQnapus3316 | 65.07 |
| C1 | UQnapus3327 | 65.14 |
| C1 | UQnapus0832 | 65.16 |
| C1 | UQnapus3314 | 65.23 |
| C1 | UQnapus3328 | 65.26 |
| C1 | UQnapus3309 | 65.35 |
| C1 | UQnapus3326 | 65.35 |
| C1 | znS06M30-310 | 66.17 |
| C1 | znS08M15-80 | 66.41 |
| C1 | UQnapus0574 | 66.78 |
| C1 | UQnapus5879 | 68.33 |
| C1 | UQnapus3342 | 68.53 |
| C1 | IGF0504b | 69.41 |
| C1 | UQnapus3343 | 69.66 |
| C1 | UQnapus0553 | 71.84 |
| C1 | UQnapus0988 | 72.35 |
| C1 | UQnapus0751 | 77.12 |
| C1 | HR-Tp3-280 | 84.26 |
| C1 | pW190b | 85.29 |
| C1 | HR-Sp2-170 | 85.98 |
| C1 | UQnapus0878 | 87.11 |
| C1 | UQnapus0426 | 87.57 |
| C1 | UQnapus0411 | 88.37 |
| C1 | UQnapus3944 | 88.78 |
| C1 | HR-S10-140 | 90.09 |
| C1 | BRMS-175 | 91.53 |
| C1 | IGF3141e | 93.75 |
| C1 | UQnapus0963 | 97.72 |
| C2 | Na12C03 | 0.00 |
| C2 | em18me6-220 | 13.99 |
| C2 | sNRE30 | 17.51 |
| C2 | Na12C12 | 21.75 |
| C2 | Ol12B03 | 28.76 |
| C2 | Ol10H02 | 28.95 |
| C2 | sORA43 | 29.62 |
| C2 | em11me5-800 | 33.33 |
| C2 | IGF3246z | 36.23 |
| C2 | em05me08-250 | 44.93 |
| C2 | em17me11-100 | 46.42 |
| C2 | SA12a | 47.86 |
| C2 | HR-Tp2-120 | 52.83 |
| C2 | HR-Sp3-450 | 54.92 |
| C2 | HR-Tp2-110 | 57.02 |
| C2 | HG-FT-C2 | 63.26 |
| C2 | Ol13G05 | 67.73 |
| C2 | sR6293b | 70.21 |
| C2 | CB10530b | 71.58 |
| C2 | sN3761b | 72.96 |
| C2 | em11me5-600 | 75.62 |
| C2 | IGF5190b | 77.03 |
| C2 | znS03M33-300 | 82.27 |
| C2 | sR12095 | 97.10 |
| C2 | pW119 | 100.33 |
| C2 | Ol11H09 | 106.90 |
| C2 | HS-b51-2 | 123.90 |
| C3 | UQnapus0119 | 0.00 |
| C3 | BRMS-093 | 1.87 |
| C3 | UQnapus2098 | 2.24 |
| C3 | UQnapus0765 | 3.40 |
| C3 | UQnapus3859 | 4.77 |
| C3 | UQnapus3858 | 5.53 |
| C3 | UQnapus0830 | 6.30 |
| C3 | UQnapus3855 | 6.49 |
| C3 | UQnapus4745 | 12.58 |
| C3 | UQnapus0620 | 19.82 |
| C3 | UQnapus3809 | 23.21 |
| C3 | UQnapus5847 | 23.38 |
| C3 | UQnapus3810 | 23.53 |
| C3 | UQnapus0443 | 27.56 |
| C3 | UQnapus0999 | 27.82 |
| C3 | UQnapus0583 | 28.26 |
| C3 | UQnapus3429 | 28.98 |
| C3 | UQnapus3785 | 31.71 |
| C3 | UQnapus3784 | 31.86 |
| C3 | UQnapus3787 | 31.94 |
| C3 | UQnapus3780 | 33.03 |
| C3 | UQnapus3779 | 33.07 |
| C3 | UQnapus3775 | 33.12 |
| C3 | UQnapus3776 | 33.12 |
| C3 | UQnapus3778 | 33.27 |
| C3 | UQnapus3777 | 33.59 |
| C3 | UQnapus0384 | 36.18 |
| C3 | UQnapus0646 | 36.52 |
| C3 | UQnapus0834 | 37.34 |
| C3 | UQnapus0573 | 38.14 |
| C3 | UQnapus4797 | 38.31 |
| C3 | znS11M22-90 | 39.27 |
| C3 | UQnapus0915 | 49.81 |
| C3 | UQnapus0106 | 51.09 |
| C3 | UQnapus0445 | 51.86 |
| C3 | UQnapus0446 | 52.55 |
| C3 | HBr062 | 52.78 |
| C3 | B044L16-5 | 54.72 |
| C3 | UQnapus3731 | 66.44 |
| C3 | UQnapus3707 | 68.53 |
| C3 | HBr083 | 68.92 |
| C3 | UQnapus4359 | 69.19 |
| C3 | UQnapus3709 | 70.05 |
| C3 | UQnapus3725 | 71.21 |
| C3 | UQnapus3720 | 72.34 |
| C3 | UQnapus4588 | 72.34 |
| C3 | UQnapus3719 | 72.47 |
| C3 | UQnapus3718 | 72.55 |
| C3 | UQnapus3722 | 85.11 |
| C4 | UQnapus5558 | 0.00 |
| C4 | UQnapus5246 | 3.11 |
| C4 | UQnapus5611 | 10.73 |
| C4 | Ol11H02a | 11.12 |
| C4 | UQnapus5612 | 11.96 |
| C4 | UQnapus5610 | 16.55 |
| C4 | MR155 | 16.58 |
| C4 | UQnapus4031 | 19.58 |
| C4 | UQnapus4784 | 22.58 |
| C4 | CB10493 | 23.98 |
| C4 | JICB0111 | 26.26 |
| C4 | JICB0149 | 29.02 |
| C4 | em18me29-100 | 33.96 |
| C4 | UQnapus5384 | 36.59 |
| C4 | IGF1002e | 37.08 |
| C4 | UQnapus5383 | 37.24 |
| C4 | UQnapus5385 | 37.83 |
| C4 | UQnapus5915 | 44.13 |
| C4 | UQnapus4573 | 47.02 |
| C4 | UQnapus5029 | 48.44 |
| C4 | UQnapus5598 | 49.78 |
| C4 | UQnapus4042 | 51.06 |
| C4 | UQnapus4044 | 51.11 |
| C4 | UQnapus4045 | 51.28 |
| C4 | UQnapus4033 | 52.80 |
| C4 | UQnapus4032 | 52.90 |
| C4 | UQnapus4039 | 53.09 |
| C4 | UQnapus5380 | 53.45 |
| C4 | UQnapus5853 | 56.26 |
| C4 | UQnapus5378 | 57.32 |
| C4 | UQnapus5196 | 57.89 |
| C4 | UQnapus5593 | 58.80 |
| C4 | UQnapus4845 | 60.53 |
| C4 | UQnapus5588 | 60.77 |
| C4 | UQnapus5913 | 60.99 |
| C4 | UQnapus5586 | 70.41 |
| C4 | MR140 | 79.38 |
| C4 | IGF3395c | 80.58 |
| C4 | pW259 | 81.59 |
| C4 | Ol12D02 | 82.23 |
| C4 | Na10C01a | 82.56 |
| C4 | BRAS061 | 83.13 |
| C4 | em18me15-561bp | 83.77 |
| C4 | em14me20-219bp | 84.19 |
| C4 | CB10530a | 84.29 |
| C4 | znS06M36-60 | 85.97 |
| C4 | znS06M32-200 | 86.26 |
| C4 | HR-C015-C4b | 86.49 |
| C4 | znS11M22-400 | 86.78 |
| C4 | UQnapus3953 | 93.72 |
| C4 | UQnapus3952 | 93.87 |
| C4 | UQnapus3931 | 94.17 |
| C4 | UQnapus3932 | 94.46 |
| C4 | UQnapus3929 | 94.47 |
| C4 | UQnapus3922 | 95.30 |
| C4 | UQnapus3917 | 95.30 |
| C4 | UQnapus3919 | 95.30 |
| C4 | UQnapus3918 | 95.30 |
| C4 | UQnapus4238 | 95.31 |
| C4 | UQnapus3921 | 95.62 |
| C4 | UQnapus3943 | 95.75 |
| C4 | UQnapus3947 | 95.82 |
| C4 | UQnapus3946 | 95.91 |
| C4 | UQnapus4938 | 96.01 |
| C4 | UQnapus3908 | 106.06 |
| C4 | UQnapus3907 | 106.40 |
| C4 | UQnapus3902 | 107.19 |
| C4 | UQnapus3905 | 107.26 |
| C4 | UQnapus3904 | 107.50 |
| C4 | UQnapus3903 | 107.60 |
| C4 | UQnapus3900 | 107.93 |
| C4 | UQnapus3901 | 108.05 |
| C4 | UQnapus3884 | 111.93 |
| C4 | UQnapus3886 | 112.19 |
| C4 | UQnapus3890 | 112.34 |
| C4 | UQnapus3883 | 112.36 |
| C4 | UQnapus3889 | 112.39 |
| C4 | UQnapus3893 | 112.66 |
| C4 | UQnapus3882 | 113.09 |
| C4 | UQnapus3895 | 113.47 |
| C4 | UQnapus3896 | 114.09 |
| C4 | UQnapus4766 | 114.50 |
| C4 | JICB0088 | 135.98 |
| C4 | BRMS-166 | 138.05 |
| C4 | JICB2056_150 | 143.09 |
| C4 | HR-Sp2-140 | 152.17 |
| C4 | HR-S4-340 | 161.57 |
| C4 | HR-Sp3-145 | 162.15 |
| C5 | UQnapus5973 | 0.00 |
| C5 | UQnapus0772 | 7.52 |
| C5 | UQnapus3300 | 12.34 |
| C5 | IGF0193C | 16.22 |
| C5 | em12me21-150 | 17.80 |
| C5 | Ol10A10 | 20.15 |
| C5 | Na10D11 | 20.49 |
| C5 | sS2131 | 20.49 |
| C5 | sS2129 | 21.10 |
| C5 | Na12E06B | 22.34 |
| C5 | RA2F11 | 25.48 |
| C5 | UQnapus0416 | 26.23 |
| C5 | UQnapus0624 | 29.41 |
| C5 | UQnapus4099 | 34.34 |
| C5 | UQnapus4100 | 35.97 |
| C5 | KBrB086M23-6_575 | 38.56 |
| C5 | IGF3112a | 40.19 |
| C5 | UQnapus4065 | 45.94 |
| C5 | UQnapus0406 | 47.84 |
| C5 | UQnapus4075 | 49.26 |
| C5 | UQnapus4081 | 54.42 |
| C5 | UQnapus4082 | 56.38 |
| C5 | JICB0509 | 63.11 |
| C5 | Ol10B02 | 65.34 |
| C5 | sORH13 | 74.33 |
| C5 | Na12G12 | 74.93 |
| C5 | UQnapus4096 | 76.91 |
| C5 | UQnapus4807 | 82.67 |
| C5 | HR-S4-230 | 88.05 |
| C5 | CB10124 | 89.08 |
| C5 | UQnapus4056 | 90.16 |
| C5 | UQnapus4057 | 93.01 |
| C5 | UQnapus0924 | 95.59 |
| C6 | IGF1226b | 0.00 |
| C6 | CNU053a | 10.76 |
| C6 | UQnapus0394 | 16.11 |
| C6 | UQnapus1012 | 19.86 |
| C6 | PA28 | 23.19 |
| C6 | em18me23-350 | 24.09 |
| C6 | JBnB061J08 | 27.59 |
| C6 | HS-028L01_AT3 | 39.39 |
| C6 | HS-Au4 | 39.58 |
| C6 | HG-AP1-C6a | 39.76 |
| C6 | pW134 | 40.03 |
| C6 | Na12A02 | 40.21 |
| C6 | Na12A05 | 40.75 |
| C6 | UQnapus4115 | 41.70 |
| C6 | UQnapus0669 | 42.28 |
| C6 | UQnapus0973 | 42.42 |
| C6 | UQnapus0821 | 43.58 |
| C6 | UQnapus0816 | 43.65 |
| C6 | UQnapus0052 | 43.76 |
| C6 | UQnapus4105 | 43.79 |
| C6 | UQnapus4108 | 43.79 |
| C6 | UQnapus4901 | 43.80 |
| C6 | UQnapus0104 | 43.88 |
| C6 | BOG44-1 | 46.60 |
| C6 | UQnapus4278 | 47.88 |
| C6 | UQnapus4760 | 50.27 |
| C6 | BRMS-015 | 51.69 |
| C6 | UQnapus0907 | 52.97 |
| C6 | em15me28-300 | 56.21 |
| C6 | CB10010 | 60.92 |
| C6 | HS-WA3 | 61.73 |
| C6 | IGF3380b | 62.00 |
| C6 | UQnapus4153 | 62.73 |
| C6 | HBr025 | 65.66 |
| C6 | MR133.1 | 67.14 |
| C6 | UQnapus0911 | 69.67 |
| C6 | UQnapus0604 | 69.67 |
| C6 | UQnapus0896 | 70.05 |
| C6 | UQnapus3149 | 70.27 |
| C6 | UQnapus0594 | 70.46 |
| C6 | UQnapus0806 | 70.57 |
| C6 | UQnapus3878 | 72.79 |
| C6 | UQnapus3875 | 72.90 |
| C6 | UQnapus4817 | 72.93 |
| C6 | UQnapus3876 | 72.93 |
| C6 | UQnapus3877 | 73.14 |
| C6 | UQnapus0351 | 73.43 |
| C6 | UQnapus0974 | 73.77 |
| C6 | HR-C005-C6 | 79.26 |
| C6 | HBr047 | 80.79 |
| C6 | HBr057 | 81.66 |
| C6 | UQnapus0771 | 85.48 |
| C6 | UQnapus3842 | 89.15 |
| C6 | UQnapus4502 | 89.33 |
| C6 | UQnapus5067 | 89.39 |
| C6 | UQnapus3853 | 90.79 |
| C6 | UQnapus0888 | 90.79 |
| C6 | HG-TAG1-C6 | 93.84 |
| C6 | UQnapus1040 | 96.09 |
| C7 | HR-Sp1-300 | 0.00 |
| C7 | UQnapus3147 | 2.61 |
| C7 | UQnapus3145 | 3.58 |
| C7 | HR-S1-275 | 8.04 |
| C7 | HBr041 | 10.12 |
| C7 | H111N19-5 | 11.71 |
| C7 | UQnapus3393 | 16.31 |
| C7 | UQnapus3396 | 18.60 |
| C7 | UQnapus3399 | 20.87 |
| C7 | UQnapus3400 | 21.31 |
| C7 | UQnapus3404 | 21.79 |
| C7 | UQnapus3405 | 22.31 |
| C7 | HBr058 | 24.82 |
| C7 | UQnapus4386 | 25.99 |
| C7 | UQnapus4390 | 27.32 |
| C7 | UQnapus4397 | 28.15 |
| C7 | UQnapus4810 | 31.90 |
| C7 | UQnapus4377 | 33.17 |
| C7 | UQnapus4366 | 33.38 |
| C7 | UQnapus4367 | 33.44 |
| C7 | UQnapus4372 | 33.49 |
| C7 | UQnapus4371 | 33.49 |
| C7 | UQnapus4373 | 33.61 |
| C7 | UQnapus4375 | 34.11 |
| C7 | UQnapus0010 | 34.17 |
| C7 | UQnapus4376 | 34.46 |
| C7 | UQnapus4380 | 34.57 |
| C7 | UQnapus4379 | 34.63 |
| C7 | HS-Au39a | 36.01 |
| C7 | HBr080 | 37.73 |
| C7 | UQnapus4818 | 38.22 |
| C7 | UQnapus4349 | 40.57 |
| C7 | znS06M36-220 | 41.41 |
| C7 | B068N22-1 | 44.51 |
| C7 | IGF5415a | 45.52 |
| C7 | IGF5707b | 48.22 |
| C7 | UQnapus4945 | 48.45 |
| C7 | IGF5706y | 48.86 |
| C7 | UQnapus4317 | 50.33 |
| C7 | UQnapus4327 | 51.23 |
| C7 | UQnapus4324 | 51.93 |
| C7 | UQnapus4313 | 52.22 |
| C7 | IGF5702e | 53.23 |
| C7 | IGF3138z | 53.90 |
| C7 | UQnapus4297 | 59.47 |
| C7 | UQnapus4303 | 59.49 |
| C7 | UQnapus4304 | 59.61 |
| C7 | UQnapus4908 | 59.66 |
| C7 | UQnapus4296 | 59.78 |
| C7 | UQnapus4299 | 59.92 |
| C7 | UQnapus4298 | 59.92 |
| C7 | UQnapus4301 | 60.45 |
| C7 | CNU400 | 61.66 |
| C7 | IGF2562d | 62.80 |
| C7 | UQnapus0731 | 75.39 |
| C7 | UQnapus2433 | 87.01 |
| C7 | sNRH63 | 94.09 |
| C7 | BRAS019 | 94.39 |
| C7 | Na10C01b | 95.81 |
| C7 | sN0706 | 101.93 |
| C8 | IGF5193c | 0.00 |
| C8 | UQnapus4531 | 3.95 |
| C8 | UQnapus5806 | 3.95 |
| C8 | UQnapus4532 | 6.27 |
| C8 | UQnapus4536 | 8.43 |
| C8 | UQnapus2785 | 13.87 |
| C8 | CB10028 | 16.02 |
| C8 | IGF1048c | 17.84 |
| C8 | UQnapus4550 | 21.53 |
| C8 | UQnapus0059 | 22.18 |
| C8 | UQnapus4554 | 22.18 |
| C8 | UQnapus4547 | 22.66 |
| C8 | UQnapus4542 | 25.22 |
| C8 | UQnapus5429 | 25.61 |
| C8 | UQnapus5722 | 26.43 |
| C8 | UQnapus0480 | 33.84 |
| C8 | UQnapus5427 | 37.43 |
| C8 | UQnapus4512 | 37.73 |
| C8 | UQnapus4510 | 37.80 |
| C8 | UQnapus4511 | 37.96 |
| C8 | UQnapus4508 | 38.74 |
| C8 | UQnapus4507 | 42.42 |
| C8 | UQnapus4503 | 44.03 |
| C8 | UQnapus4504 | 44.14 |
| C8 | UQnapus5151 | 44.20 |
| C8 | UQnapus4505 | 44.20 |
| C8 | UQnapus4497 | 46.61 |
| C8 | UQnapus4496 | 46.77 |
| C8 | UQnapus4486 | 47.74 |
| C8 | UQnapus4482 | 48.47 |
| C8 | UQnapus4483 | 48.56 |
| C8 | UQnapus5776 | 49.96 |
| C8 | UQnapus5775 | 50.03 |
| C8 | UQnapus4472 | 50.15 |
| C8 | UQnapus4517 | 52.54 |
| C8 | UQnapus4468 | 52.91 |
| C8 | UQnapus4523 | 53.29 |
| C8 | UQnapus4518 | 53.84 |
| C8 | UQnapus4519 | 53.94 |
| C8 | UQnapus4462 | 56.40 |
| C8 | UQnapus4461 | 57.20 |
| C8 | UQnapus4458 | 57.74 |
| C8 | UQnapus4459 | 57.79 |
| C8 | UQnapus4448 | 60.29 |
| C8 | UQnapus4449 | 60.34 |
| C8 | UQnapus5317 | 60.53 |
| C8 | UQnapus4440 | 60.83 |
| C8 | UQnapus4915 | 60.83 |
| C8 | UQnapus4442 | 60.93 |
| C8 | UQnapus4441 | 61.03 |
| C8 | UQnapus4436 | 62.82 |
| C8 | UQnapus5957 | 63.07 |
| C8 | UQnapus5057 | 64.56 |
| C8 | UQnapus2833 | 64.64 |
| C8 | UQnapus4417 | 64.91 |
| C8 | UQnapus4733 | 65.27 |
| C8 | UQnapus4420 | 65.36 |
| C8 | em18me6-100 | 69.61 |
| C8 | UQnapus2853 | 71.58 |
| C8 | KBrB088I08-12 | 75.96 |
| C8 | Ol12G04 | 81.09 |
| C8 | Na12B05b | 88.98 |
| C9 | pw155a | 0.00 |
| C9 | UQnapus4625 | 7.99 |
| C9 | HG4-BSR8 | 8.94 |
| C9 | UQnapus0502 | 24.66 |
| C9 | HR-C017-C9 | 28.64 |
| C9 | UQnapus0439 | 30.40 |
| C9 | UQnapus4607 | 30.61 |
| C9 | UQnapus4606 | 30.65 |
| C9 | UQnapus1026 | 30.85 |
| C9 | UQnapus0535 | 31.51 |
| C9 | UQnapus0797 | 31.82 |
| C9 | UQnapus0976 | 32.61 |
| C9 | UQnapus4610 | 35.45 |
| C9 | UQnapus1000 | 39.41 |
| C9 | UQnapus4293 | 41.01 |
| C9 | H099I08-130 | 44.83 |
| C9 | pW233b | 46.19 |
| C9 | UQnapus2936 | 50.36 |
| C9 | em17me21-400 | 52.26 |
| C9 | B068A23-1 | 53.06 |
| C9 | B039K15-5-1 | 53.39 |
| C9 | HBr071 | 55.79 |
| C9 | HBr087 | 56.53 |
| C9 | HBr088 | 58.87 |
| C9 | IGF1072c | 59.21 |
| C9 | BRMS-154 | 61.34 |
| C9 | UQnapus4591 | 64.91 |
| C9 | UQnapus4592 | 66.57 |
| C9 | UQnapus4594 | 67.55 |
| C9 | CB10064 | 84.56 |
| C9 | UQnapus3635 | 87.51 |
| C9 | UQnapus4586 | 89.74 |
| C9 | UQnapus4966 | 89.99 |
| C9 | UQnapus3634 | 97.63 |
| C9 | HBr068 | 102.83 |
