# Supplementary material for: Genome Wide Analysis of Flowering Time Trait in Multiple Environments via High-Throughput Genotyping Technique in Brassica napus L
Source: PLoS One. 2015 Mar 19;10(3):e0119425. doi: 10.1371/journal.pone.0119425 (PMC4366152; doi:10.1371/journal.pone.0119425)
Supplement: S4 Table — (DOCX) [file pone.0119425.s006.docx]

**S4 Table.** Functional clusters of genes tagged by associated SNPs with enrichment score > 1.3 (corresponding to p value of 0.05)

| Function clusters | Category | Term | Count | % | PValue | Genes | List Total | Pop Hits | Pop Total | Fold Enrichment |
| --- | --- | --- | --- | --- | --- | --- | --- | --- | --- | --- |
| Cluster 1  (Enrichment Score: 1.52) | GOTERM_BP_5 | GO:0009755~hormone-mediated signaling | 12 | 4.26 | 0.02 | AT4G39780, AT2G36910, AT4G23750, AT2G35700, AT5G61850, AT4G18710, AT5G10510, AT5G52300, AT5G53290, AT3G20770, AT5G65430, AT4G16750 | 146 | 432 | 11911 | 2.27 |
|  | GOTERM_BP_5 | GO:0009873~ethylene mediated signaling pathway | 7 | 2.48 | 0.02 | AT4G39780, AT4G23750, AT2G35700, AT5G10510, AT5G53290, AT3G20770, AT4G16750 | 146 | 170 | 11911 | 3.36 |
|  | GOTERM_BP_5 | GO:0000160~two-component signal transduction system (phosphorelay) | 7 | 2.48 | 0.05 | AT4G39780, AT4G23750, AT2G35700, AT5G10510, AT5G53290, AT3G20770, AT4G16750 | 146 | 218 | 11911 | 2.62 |
|  | GOTERM_BP_5 | GO:0007242~intracellular signaling cascade | 16 | 5.67 | 0.06 | AT4G39780, AT2G35700, AT5G61850, AT5G53290, AT3G54840, AT3G22950, AT4G23750, AT2G36910, AT4G18130, AT5G10510, AT4G18710, AT5G52300, AT3G20770, AT5G21274, AT5G65430, AT4G16750 | 146 | 790 | 11911 | 1.65 |
| Cluster 2  (Enrichment Score: 1.38) | GOTERM_BP_5 | GO:0009908~flower development | 9 | 3.19 | 0.01 | AT2G36910, AT5G61850, AT4G29010, AT5G10510, AT4G24540, AT2G45660, AT4G36380, AT1G69120, AT3G28470 | 146 | 232 | 11911 | 3.16 |
|  | GOTERM_BP_5 | GO:0010074~maintenance of meristem identity | 3 | 1.06 | 0.02 | AT5G61850, AT4G24540, AT1G69120 | 146 | 19 | 11911 | 12.88 |
|  | GOTERM_BP_5 | GO:0010073~meristem maintenance | 4 | 1.42 | 0.03 | AT5G61850, AT5G10510, AT4G24540, AT1G69120 | 146 | 54 | 11911 | 6.04 |
|  | GOTERM_BP_5 | GO:0019827~stem cell maintenance | 3 | 1.06 | 0.03 | AT5G61850, AT4G24540, AT1G69120 | 146 | 24 | 11911 | 10.20 |
|  | GOTERM_BP_5 | GO:0048864~stem cell development | 3 | 1.06 | 0.04 | AT5G61850, AT4G24540, AT1G69120 | 146 | 25 | 11911 | 9.79 |
|  | GOTERM_BP_5 | GO:0045596~negative regulation of cell differentiation | 3 | 1.06 | 0.04 | AT5G61850, AT4G24540, AT1G69120 | 146 | 27 | 11911 | 9.06 |
|  | GOTERM_BP_5 | GO:0048507~meristem development | 4 | 1.42 | 0.10 | AT5G61850, AT5G10510, AT4G24540, AT1G69120 | 146 | 92 | 11911 | 3.55 |
|  | GOTERM_BP_5 | GO:0009888~tissue development | 5 | 1.77 | 0.35 | AT5G61850, AT5G10510, AT4G24540, AT3G12280, AT1G69120 | 146 | 245 | 11911 | 1.66 |
| Cluster 3  (Enrichment Score: 1.37) | GOTERM_CC_5 | GO:0005634~nucleus | 57 | 20.21 | 0.01 | AT4G39780, AT4G29940, AT3G57060, AT2G02230, AT5G09850, AT4G26600, AT4G36870, AT3G02150, AT4G23750, AT1G60900, AT1G68920, AT3G20770, AT2G45660, AT5G65430, AT4G33400, AT2G34440, AT5G53290, AT1G80070, AT3G14420, AT4G24540, AT4G17890, AT2G30800, AT4G29100, AT3G58660, AT4G34530, AT1G68520, AT5G57900, AT5G10510, AT4G40030, AT2G36010, AT4G05410, AT4G19990, AT4G11740, AT3G28470, AT4G20280, AT2G35700, AT5G61850, AT3G12280, AT1G69120, AT2G40030, AT3G05690, AT5G51660, AT2G41630, AT5G02500, AT2G34140, AT4G27640, AT5G04990, AT2G30250, AT1G48950, AT4G02460, AT4G35800, AT1G70000, AT5G55230, AT3G54620, AT4G16750, AT1G05805, AT5G39660 | 150 | 3353 | 11729 | 1.33 |
|  | GOTERM_BP_5 | GO:0045449~regulation of transcription | 35 | 12.41 | 0.03 | AT4G39780, AT4G29940, AT2G35700, AT5G61850, AT3G56520, AT4G36870, AT1G69120, AT3G12280, AT3G02150, AT4G23750, AT3G18400, AT3G05690, AT1G68920, AT3G20770, AT2G45660, AT2G41630, AT2G34440, AT4G29000, AT2G34140, AT5G53290, AT2G30250, AT4G24540, AT4G29100, AT1G76900, AT1G70000, AT4G34530, AT1G68520, AT4G18130, AT5G10510, AT3G54620, AT2G36010, AT4G16750, AT1G05805, AT5G39660, AT3G28470 | 146 | 2024 | 11911 | 1.41 |
|  | GOTERM_BP_5 | GO:0019219~regulation of nucleobase, nucleoside, nucleotide and nucleic acid metabolic process | 35 | 12.41 | 0.03 | AT4G39780, AT4G29940, AT2G35700, AT5G61850, AT3G56520, AT4G36870, AT1G69120, AT3G12280, AT3G02150, AT4G23750, AT3G18400, AT3G05690, AT1G68920, AT3G20770, AT2G45660, AT2G41630, AT2G34440, AT4G29000, AT2G34140, AT5G53290, AT2G30250, AT4G24540, AT4G29100, AT1G76900, AT1G70000, AT4G34530, AT1G68520, AT4G18130, AT5G10510, AT3G54620, AT2G36010, AT4G16750, AT1G05805, AT5G39660, AT3G28470 | 146 | 2051 | 11911 | 1.39 |
|  | GOTERM_BP_5 | GO:0010556~regulation of macromolecule biosynthetic process | 35 | 12.41 | 0.04 | AT4G39780, AT4G29940, AT2G35700, AT5G61850, AT3G56520, AT4G36870, AT1G69120, AT3G12280, AT3G02150, AT4G23750, AT3G18400, AT3G05690, AT1G68920, AT3G20770, AT2G45660, AT2G41630, AT2G34440, AT4G29000, AT2G34140, AT5G53290, AT2G30250, AT4G24540, AT4G29100, AT1G76900, AT1G70000, AT4G34530, AT1G68520, AT4G18130, AT5G10510, AT3G54620, AT2G36010, AT4G16750, AT1G05805, AT5G39660, AT3G28470 | 146 | 2070 | 11911 | 1.38 |
|  | GOTERM_BP_5 | GO:0031326~regulation of cellular biosynthetic process | 35 | 12.41 | 0.04 | AT4G39780, AT4G29940, AT2G35700, AT5G61850, AT3G56520, AT4G36870, AT1G69120, AT3G12280, AT3G02150, AT4G23750, AT3G18400, AT3G05690, AT1G68920, AT3G20770, AT2G45660, AT2G41630, AT2G34440, AT4G29000, AT2G34140, AT5G53290, AT2G30250, AT4G24540, AT4G29100, AT1G76900, AT1G70000, AT4G34530, AT1G68520, AT4G18130, AT5G10510, AT3G54620, AT2G36010, AT4G16750, AT1G05805, AT5G39660, AT3G28470 | 146 | 2101 | 11911 | 1.36 |
|  | GOTERM_BP_5 | GO:0010468~regulation of gene expression | 36 | 12.77 | 0.05 | AT4G39780, AT4G29940, AT2G35700, AT5G61850, AT3G56520, AT3G12280, AT4G36870, AT1G69120, AT2G40030, AT3G02150, AT4G23750, AT3G18400, AT3G05690, AT1G68920, AT3G20770, AT2G45660, AT2G41630, AT2G34440, AT4G29000, AT2G34140, AT5G53290, AT2G30250, AT4G24540, AT4G29100, AT1G76900, AT1G70000, AT4G34530, AT1G68520, AT4G18130, AT5G10510, AT3G54620, AT2G36010, AT4G16750, AT1G05805, AT5G39660, AT3G28470 | 146 | 2197 | 11911 | 1.34 |
|  | GOTERM_BP_5 | GO:0006355~regulation of transcription, DNA-dependent | 19 | 6.74 | 0.11 | AT4G39780, AT4G29940, AT2G35700, AT5G53290, AT2G30250, AT4G24540, AT1G69120, AT4G36870, AT4G23750, AT4G18130, AT5G10510, AT3G05690, AT3G54620, AT2G36010, AT2G45660, AT4G16750, AT3G28470, AT2G34440, AT2G41630 | 146 | 1089 | 11911 | 1.42 |
|  | GOTERM_BP_5 | GO:0051252~regulation of RNA metabolic process | 19 | 6.74 | 0.12 | AT4G39780, AT4G29940, AT2G35700, AT5G53290, AT2G30250, AT4G24540, AT1G69120, AT4G36870, AT4G23750, AT4G18130, AT5G10510, AT3G05690, AT3G54620, AT2G36010, AT2G45660, AT4G16750, AT3G28470, AT2G34440, AT2G41630 | 146 | 1095 | 11911 | 1.42 |
