# Supplementary material for: Genome Wide Analysis of Flowering Time Trait in Multiple Environments via High-Throughput Genotyping Technique in Brassica napus L
Source: PLoS One. 2015 Mar 19;10(3):e0119425. doi: 10.1371/journal.pone.0119425 (PMC4366152; doi:10.1371/journal.pone.0119425)
Supplement: S5 Table — (DOCX) [file pone.0119425.s007.docx]

**S5 Table.** Top three functional groups of genes represented by SNPs common to all three geographic sites and specific to one or two regions respectively.

| Gene Set | Functional Clusters | Category | Term | Count | % | Genes | Fold Enrichment |
| --- | --- | --- | --- | --- | --- | --- | --- |
| Common Genes | Cluster 1 (Enrichment Score: 0.90) | GOTERM_BP_5 | GO:0006397~mRNA processing | 3 | 3.49 | AT1G60900, AT1G80070, AT5G51660 | 5.75 |
|  |  | GOTERM_BP_5 | GO:0016070~RNA metabolic process | 6 | 6.98 | AT4G14790, AT1G60900, AT1G80070, AT4G26600, AT5G51660, AT4G20280 | 2.38 |
|  |  | GOTERM_BP_5 | GO:0016071~mRNA metabolic process | 3 | 3.49 | AT1G60900, AT1G80070, AT5G51660 | 5.01 |
|  |  | GOTERM_BP_5 | GO:0006396~RNA processing | 4 | 4.65 | AT1G60900, AT1G80070, AT4G26600, AT5G51660 | 2.38 |
|  | Cluster 2 (Enrichment Score: 0.84) | GOTERM_CC_5 | GO:0005634~nucleus | 20 | 23.26 | AT4G29940, AT2G34140, AT5G04990, AT1G80070, AT4G17890, AT4G29100, AT4G26600, AT1G70000, AT1G69120, AT1G68520, AT4G23750, AT5G10510, AT1G60900, AT5G55230, AT1G68920, AT5G65430, AT4G19990, AT5G51660, AT2G34440, AT4G20280 | 1.63 |
|  |  | GOTERM_BP_5 | GO:0045449~regulation of transcription | 12 | 13.95 | AT4G29000, AT4G29940, AT2G34140, AT4G23750, AT4G18130, AT5G10510, AT1G68920, AT4G29100, AT1G70000, AT1G69120, AT1G68520, AT2G34440 | 1.54 |
|  |  | GOTERM_BP_5 | GO:0019219~regulation of nucleobase, nucleoside, nucleotide and nucleic acid metabolic process | 12 | 13.95 | AT4G29000, AT4G29940, AT2G34140, AT4G23750, AT4G18130, AT5G10510, AT1G68920, AT4G29100, AT1G70000, AT1G69120, AT1G68520, AT2G34440 | 1.51 |
|  |  | GOTERM_BP_5 | GO:0010556~regulation of macromolecule biosynthetic process | 12 | 13.95 | AT4G29000, AT4G29940, AT2G34140, AT4G23750, AT4G18130, AT5G10510, AT1G68920, AT4G29100, AT1G70000, AT1G69120, AT1G68520, AT2G34440 | 1.50 |
|  |  | GOTERM_BP_5 | GO:0031326~regulation of cellular biosynthetic process | 12 | 13.95 | AT4G29000, AT4G29940, AT2G34140, AT4G23750, AT4G18130, AT5G10510, AT1G68920, AT4G29100, AT1G70000, AT1G69120, AT1G68520, AT2G34440 | 1.48 |
|  |  | GOTERM_BP_5 | GO:0010468~regulation of gene expression | 12 | 13.95 | AT4G29000, AT4G29940, AT2G34140, AT4G23750, AT4G18130, AT5G10510, AT1G68920, AT4G29100, AT1G70000, AT1G69120, AT1G68520, AT2G34440 | 1.41 |
|  |  | GOTERM_BP_5 | GO:0006355~regulation of transcription, DNA-dependent | 6 | 6.98 | AT4G29940, AT4G23750, AT4G18130, AT5G10510, AT1G69120, AT2G34440 | 1.43 |
|  |  | GOTERM_BP_5 | GO:0051252~regulation of RNA metabolic process | 6 | 6.98 | AT4G29940, AT4G23750, AT4G18130, AT5G10510, AT1G69120, AT2G34440 | 1.42 |
|  | Cluster 3 (Enrichment Score: 0.50) | GOTERM_CC_5 | GO:0043229~intracellular organelle | 38 | 44.19 | AT4G29940, AT4G25720, AT4G29010, AT5G05980, AT5G05670, AT4G18350, AT1G67360, AT4G26600, AT1G69120, AT4G28290, AT1G59820, AT4G23750, AT1G60900, AT4G39550, AT1G68920, AT5G23740, AT5G65430, AT5G51660, AT2G34440, AT5G05360, AT2G34140, AT5G04990, AT1G80070, AT4G17890, AT4G29100, AT1G70000, AT1G68520, AT4G14790, AT2G02080, AT1G61790, AT5G10510, AT5G04140, AT5G55230, AT3G51440, AT1G66970, AT4G28710, AT4G19990, AT4G20280 | 1.16 |
|  |  | GOTERM_CC_5 | GO:0043231~intracellular membrane-bounded organelle | 36 | 41.86 | AT4G29940, AT4G25720, AT4G29010, AT5G05980, AT5G05670, AT4G18350, AT1G67360, AT4G26600, AT1G69120, AT4G28290, AT1G59820, AT4G23750, AT1G60900, AT4G39550, AT1G68920, AT5G65430, AT5G51660, AT5G05360, AT2G34440, AT2G34140, AT5G04990, AT1G80070, AT4G17890, AT4G29100, AT1G70000, AT1G68520, AT4G14790, AT2G02080, AT1G61790, AT5G10510, AT5G04140, AT5G55230, AT3G51440, AT1G66970, AT4G19990, AT4G20280 | 1.15 |
|  |  | GOTERM_CC_5 | GO:0009507~chloroplast | 12 | 13.95 | AT2G02080, AT5G05980, AT4G29010, AT1G61790, AT4G39550, AT5G04140, AT1G80070, AT4G18350, AT5G65430, AT1G66970, AT4G28290, AT5G05360 | 1.03 |
|  |  | GOTERM_CC_5 | GO:0009536~plastid | 12 | 13.95 | AT2G02080, AT5G05980, AT4G29010, AT1G61790, AT4G39550, AT5G04140, AT1G80070, AT4G18350, AT5G65430, AT1G66970, AT4G28290, AT5G05360 | 1.00 |
|  |  | GOTERM_CC_5 | GO:0044444~cytoplasmic part | 21 | 24.42 | AT4G29010, AT5G05980, AT4G25720, AT5G05670, AT5G04990, AT1G80070, AT4G18350, AT1G67360, AT4G28290, AT4G14790, AT1G59820, AT2G02080, AT1G61790, AT4G39550, AT5G04140, AT5G55230, AT3G51440, AT5G23740, AT5G65430, AT1G66970, AT5G05360 | 0.90 |
|  |  | GOTERM_CC_5 | GO:0005737~cytoplasm | 22 | 25.58 | AT4G29010, AT5G05980, AT4G25720, AT5G05670, AT5G04990, AT1G80070, AT4G18350, AT1G67360, AT4G28290, AT4G14790, AT1G59820, AT4G23750, AT2G02080, AT1G61790, AT4G39550, AT5G04140, AT5G55230, AT3G51440, AT5G23740, AT5G65430, AT1G66970, AT5G05360 | 0.85 |
| Specific genes | Cluster 1 (Enrichment Score: 1.45) | GOTERM_BP_5 | GO:0009965~leaf morphogenesis | 5 | 2.55 | AT4G18710, AT4G36380, AT3G12280, AT4G36870, AT3G02150 | 5.46 |
|  |  | GOTERM_BP_5 | GO:0048827~phyllome development | 6 | 3.06 | AT4G18710, AT5G53290, AT4G36380, AT3G12280, AT4G36870, AT3G02150 | 3.50 |
|  |  | GOTERM_BP_5 | GO:0010016~shoot morphogenesis | 5 | 2.55 | AT4G18710, AT4G36380, AT3G12280, AT4G36870, AT3G02150 | 3.97 |
|  |  | GOTERM_BP_5 | GO:0009887~organ morphogenesis | 5 | 2.55 | AT4G18710, AT4G36380, AT3G12280, AT4G36870, AT3G02150 | 3.54 |
|  |  | GOTERM_BP_5 | GO:0048367~shoot development | 6 | 3.06 | AT4G18710, AT5G53290, AT4G36380, AT3G12280, AT4G36870, AT3G02150 | 2.53 |
|  | Cluster 2 (Enrichment Score: 1.30) | GOTERM_MF_5 | GO:0046527~glucosyltransferase activity | 6 | 3.06 | AT2G21770, AT1G23480, AT5G09870, AT2G43820, AT4G39350, AT5G64740 | 4.94 |
|  |  | GOTERM_MF_5 | GO:0035251~UDP-glucosyltransferase activity | 5 | 2.55 | AT2G21770, AT5G09870, AT2G43820, AT4G39350, AT5G64740 | 4.73 |
|  |  | GOTERM_BP_5 | GO:0044042~glucan metabolic process | 5 | 2.55 | AT2G21770, AT5G09870, AT1G48930, AT4G39350, AT5G64740 | 3.94 |
|  |  | GOTERM_BP_5 | GO:0033692~cellular polysaccharide biosynthetic process | 4 | 2.04 | AT2G21770, AT5G09870, AT4G39350, AT5G64740 | 4.63 |
|  |  | GOTERM_BP_5 | GO:0000271~polysaccharide biosynthetic process | 4 | 2.04 | AT2G21770, AT5G09870, AT4G39350, AT5G64740 | 4.45 |
|  |  | GOTERM_BP_5 | GO:0006073~cellular glucan metabolic process | 4 | 2.04 | AT2G21770, AT5G09870, AT4G39350, AT5G64740 | 3.84 |
|  |  | GOTERM_BP_5 | GO:0044264~cellular polysaccharide metabolic process | 4 | 2.04 | AT2G21770, AT5G09870, AT4G39350, AT5G64740 | 3.09 |
|  |  | GOTERM_BP_5 | GO:0034637~cellular carbohydrate biosynthetic process | 4 | 2.04 | AT2G21770, AT5G09870, AT4G39350, AT5G64740 | 2.52 |
|  | Cluster 3 (Enrichment Score: 1.02) | GOTERM_BP_5 | GO:0009908~flower development | 6 | 3.06 | AT2G36910, AT5G61850, AT4G24540, AT2G45660, AT4G36380, AT3G28470 | 3.08 |
|  |  | GOTERM_BP_5 | GO:0048466~androecium development | 3 | 1.53 | AT2G36910, AT4G36380, AT3G28470 | 8.31 |
|  |  | GOTERM_BP_5 | GO:0048443~stamen development | 3 | 1.53 | AT2G36910, AT4G36380, AT3G28470 | 8.31 |
|  |  | GOTERM_BP_5 | GO:0048438~floral whorl development | 4 | 2.04 | AT2G36910, AT4G24540, AT4G36380, AT3G28470 | 4.49 |
|  |  | GOTERM_BP_5 | GO:0048437~floral organ development | 3 | 1.53 | AT2G36910, AT4G36380, AT3G28470 | 2.88 |
|  |  | GOTERM_BP_5 | GO:0048569~post-embryonic organ development | 3 | 1.53 | AT2G36910, AT4G36380, AT3G28470 | 2.01 |
