# Supplementary material for: Genome Wide Analysis of Flowering Time Trait in Multiple Environments via High-Throughput Genotyping Technique in Brassica napus L
Source: PLoS One. 2015 Mar 19;10(3):e0119425. doi: 10.1371/journal.pone.0119425 (PMC4366152; doi:10.1371/journal.pone.0119425)
Supplement: S6 Table — (DOCX) [file pone.0119425.s008.docx]

**S6 Table.** All the pair of interacting SNPs detected in the epistasis analysis

| Geographic sites | SNP Partner1 | SNP Partner2 |
| --- | --- | --- |
| North | UQnapus1298 | UQnapus4622 |
|  | UQnapus0689 | UQnapus1026 |
|  | UQnapus4096 | UQnapus4622 |
|  | **UQnapus0669** | **UQnapus3878** |
|  | UQnapus3669 | **UQnapus3878** |
|  | UQnapus0907 | **UQnapus1450** |
|  | **UQnapus0669** | UQnapus1545 |
|  | UQnapus0689 | **UQnapus0743** |
|  | **UQnapus0669** | UQnapus1298 |
| South | UQnapus1166 | UQnapus5988 |
|  | UQnapus1545 | UQnapus1709 |
|  | UQnapus1709 | UQnapus5471 |
|  | UQnapus0907 | UQnapus1709 |
|  | UQnapus1166 | **UQnapus1450** |
|  | UQnapus1644 | UQnapus5471 |
|  | UQnapus1022 | **UQnapus1450** |
|  | UQnapus0689 | UQnapus1166 |
|  | **UQnapus0669** | **UQnapus5033** |
|  | **UQnapus1106** | UQnapus1166 |
|  | UQnapus1166 | UQnapus3842 |
|  | **UQnapus0669** | UQnapus4153 |
|  | UQnapus1166 | UQnapus5307 |
|  | UQnapus0743 | UQnapus0863 |
|  | **UQnapus0669** | UQnapus1545 |
|  | UQnapus4219 | UQnapus5988 |
|  | **UQnapus0669** | UQnapus3837 |
| East | UQnapus2989 | UQnapus3319 |
|  | UQnapus2989 | UQnapus3319 |
|  | UQnapus0907 | UQnapus1298 |
|  | **UQnapus1450** | **UQnapus5033** |
|  | UQnapus4075 | **UQnapus5033** |
|  | **UQnapus0669** | UQnapus3319 |
|  | **UQnapus0238** | UQnapus4075 |
|  | **UQnapus0669** | **UQnapus4810** |
|  | UQnapus5441 | UQnapus5471 |
|  | UQnapus2989 | UQnapus5471 |
|  | **UQnapus0238** | **UQnapus1789** |
|  | UQnapus1298 | **UQnapus2347** |
|  | **UQnapus0669** | UQnapus5441 |
|  | UQnapus0895 | UQnapus5471 |
|  | UQnapus0863 | UQnapus5471 |
|  | UQnapus0394 | **UQnapus1450** |

SNPs tagging known FT genes are in bold.
